# Cholesterol-responsive NFE2L1-INSIG1 interaction controls VLDL secretion and metabolic dysfunction–associated steatohepatitis pathogenesis in mice

**DOI:** 10.1172/JCI197094

**Published:** 2026-07-15

**Authors:** Shijun Deng, Jessica E. Freed, Grace Y. Lee, Gizel Askin, Zhe Cao, Özgür Cakici, Bo Yuan, Sheng Tony Hui, Karen E. Inouye, Isabel Graupera, Gökhan S. Hotamışlıgil

**Affiliations:** 1 Department of Molecular Metabolism and Center for Prevention and Treatment of Cardiovascular Disease, Harvard TH Chan School of Public Health, Boston, Massachusetts, USA.; 2 Liver Unit Hospital Clínic, Faculty of Medicine and Health Services, University of Barcelona, Barcelona, Spain.; 3 Fundació de Recerca Clínic Barcelona-Institut d’Investigacions Biomèdiques August Pi i Sunyer (FRCB-IDIBAPS), Centro de Investigación Biomédica en Red de Enfermedades Hepáticas y Digestivas (CiberEHD), Barcelona, Spain; 4 Broad Institute of Harvard and MIT, Cambridge, Massachusetts, USA.

**Keywords:** Hepatology, Inflammation, Metabolism, Cholesterol, Lipidomics, Lipoproteins

## Abstract

Cholesterol overload contributes to metabolic dysfunction–associated steatohepatitis (MASH) progression. One major pathway that limits hepatic cholesterol accumulation is export via VLDL secretion. While sterol regulatory element–binding protein (SREBP) activity is suppressed by insulin-induced gene 1 (INSIG1) under high sterol conditions, VLDL secretion nonetheless persists to prevent lipotoxicity and liver injury, presenting an unresolved paradox in cholesterol sensing and lipoprotein export. Here, we identified a cholesterol-responsive interaction between nuclear factor erythroid 2 related factor-1 (NFE2L1) and INSIG1 that preserved cholesterol homeostasis by sustaining VLDL secretion. Liver-specific NFE2L1 deletion elevated INSIG1 abundance, suppressed SREBP1 activation, and impaired VLDL secretion, leading to hepatic cholesterol accumulation and liver injury. Mechanistically, NFE2L1 bound to INSIG1 via its N-terminal homology box 2 (NHB2) domain; free cholesterol strengthened this interaction to promote INSIG1 degradation, thereby enabling SREBP1 activation and VLDL export. In NFE2L1-deficient mice, WT NFE2L1, but not a mutant NFE2L1 form unable to interact with INSIG1 (NHB2-deleted mutant, ΔNHB2), restored SREBP1 activity and VLDL secretion. Lipidomics analysis revealed that NFE2L1 deficiency reduced serum triglyceride composition, which was restored exclusively by WT NFE2L1. In a murine MASH model, NFE2L1 overexpression activated SREBP1/2, lowered hepatic cholesterol, and attenuated liver injury, inflammation, and fibrosis, without elevating atherogenic lipoproteins owing to compensatory LDL receptor upregulation. Together, these findings explain how VLDL secretion capacity was maintained under cholesterol excess and identify the NFE2L1/INSIG1 axis as a sterol-responsive safeguard for hepatic lipid homeostasis and a potential therapeutic target for MASH.

## Abstract 2

A liver protein partnership helps prevent cholesterol buildup and liver damage, revealing a new potential target for treating fatty liver disease and related metabolic disorders.

## Graphical abstract

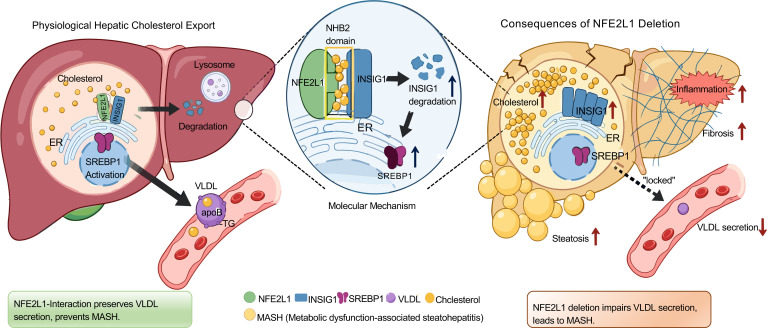

## Introduction

Metabolic dysfunction–associated steatohepatitis (MASH) affects over 5% of the global population, yet therapeutic strategies remain limited due to an incomplete understanding of hepatic cholesterol homeostasis, a central driver of disease progression (1). Cholesterol balance is critical for metabolic health, sustaining cellular functions, lipid metabolism, and physiological balance. Both cholesterol excess and deficiency disrupt cellular function and viability, necessitating adaptive mechanisms that coordinate hepatic cholesterol synthesis, storage, and secretion. The liver maintains systemic sterol balance in part by packaging triglycerides (TGs) and cholesterol into VLDL (2, 3). Impaired VLDL secretion contributes to MASH, hypercholesterolemia, and cardiovascular diseases by promoting hepatic lipid accumulation and systemic cholesterol imbalance (4–6).

The SREBP pathway orchestrates lipid biosynthesis and helps sustain VLDL secretion (7, 8). SREBPs are a family of basic helix-loop-helix leucine zipper (bHLH-LZ) transcription factors that includes SREBP1 and SREBP2: SREBP1 primarily controls genes for fatty acid and TG synthesis, whereas SREBP2 mainly regulates cholesterol biosynthetic genes. By providing lipid substrates and coordinating lipogenic programs required for apolipoprotein lipidation, SREBP1 supports the hepatic capacity to assemble and secrete TG-rich VLDL, even though cholesterol is not the dominant cargo (9). SREBP activation is governed posttranslationally by the interaction between insulin-induced gene 1 (INSIG1) and SREBP cleavage-activating protein (SCAP) at the ER, with sterol depletion permitting SCAP-SREBP trafficking to the Golgi for proteolytic activation, and sterol repletion enforcing ER retention (10). Within this framework, SREBP2 is directly responsive to cholesterol, whereas SREBP1c is regulated primarily at the transcriptional level by oxysterol-activated liver X receptors (LXRs) while still sharing the INSIG1-SCAP–mediated trafficking and processing control with SREBP2 (11, 12). Although this feedback loop restrains lipid overload, it does not fully explain how SREBP1 activity and VLDL secretion are maintained during cholesterol excess, when INSIG1 is stabilized and would be expected to chronically suppress SCAP-SREBP trafficking.

Nuclear factor erythroid-2 like 1 (NFE2L1, also known as NRF1) has emerged as a critical regulator of hepatic cholesterol homeostasis, particularly in responding to increased intracellular cholesterol levels (13). This Cap’N’Collar (CNC) transcription factor is anchored in the ER membrane and has been implicated in proteasome regulation, redox stress responses, and hepatic lipid metabolism (14, 15). Under specific stress conditions, NFE2L1 is proteolytically processed and translocates to the nucleus to drive transcriptional adaptive programs, while ER-resident forms can participate in nontranscriptional activities. Upon cholesterol excess, NFE2L1 directly binds to free cholesterol and is retained in the ER, resulting in the derepression of a program leading to cholesterol export, and its deficiency leads to hepatic cholesterol accumulation under high dietary cholesterol exposure (13, 16). However, if and how these 2 critical pathways, NFE2L1 and SREBP, responding to high and low cholesterol levels, respectively, intersect with each other to control fluctuations in cholesterol levels and coordinate proper cellular responses remain an important but unresolved question. Clarifying this connection could yield a more comprehensive understanding of cholesterol homeostasis under both deficiency and excess and its impact on systemic metabolic health.

Here, we identified a direct interaction between NFE2L1 and INSIG1 proteins that regulates SREBP1 activity and, consequently, VLDL secretion from the liver. By defining how NFE2L1 interacts with the INSIG1/SCAP/SREBP axis to preserve hepatic cholesterol balance, the present work addresses a key gap in understanding how the liver maintains VLDL export capacity while defending against cholesterol-induced lipotoxicity in MASH. Our findings bridge 2 pivotal cholesterol regulatory frameworks, positioning the NFE2L1/INSIG1 axis as a potential target for metabolic disorders.

## Results

### Hepatic NFE2L1 deficiency reduces SREBP1 activity and impairs lipid secretion.

**Figure 1:**
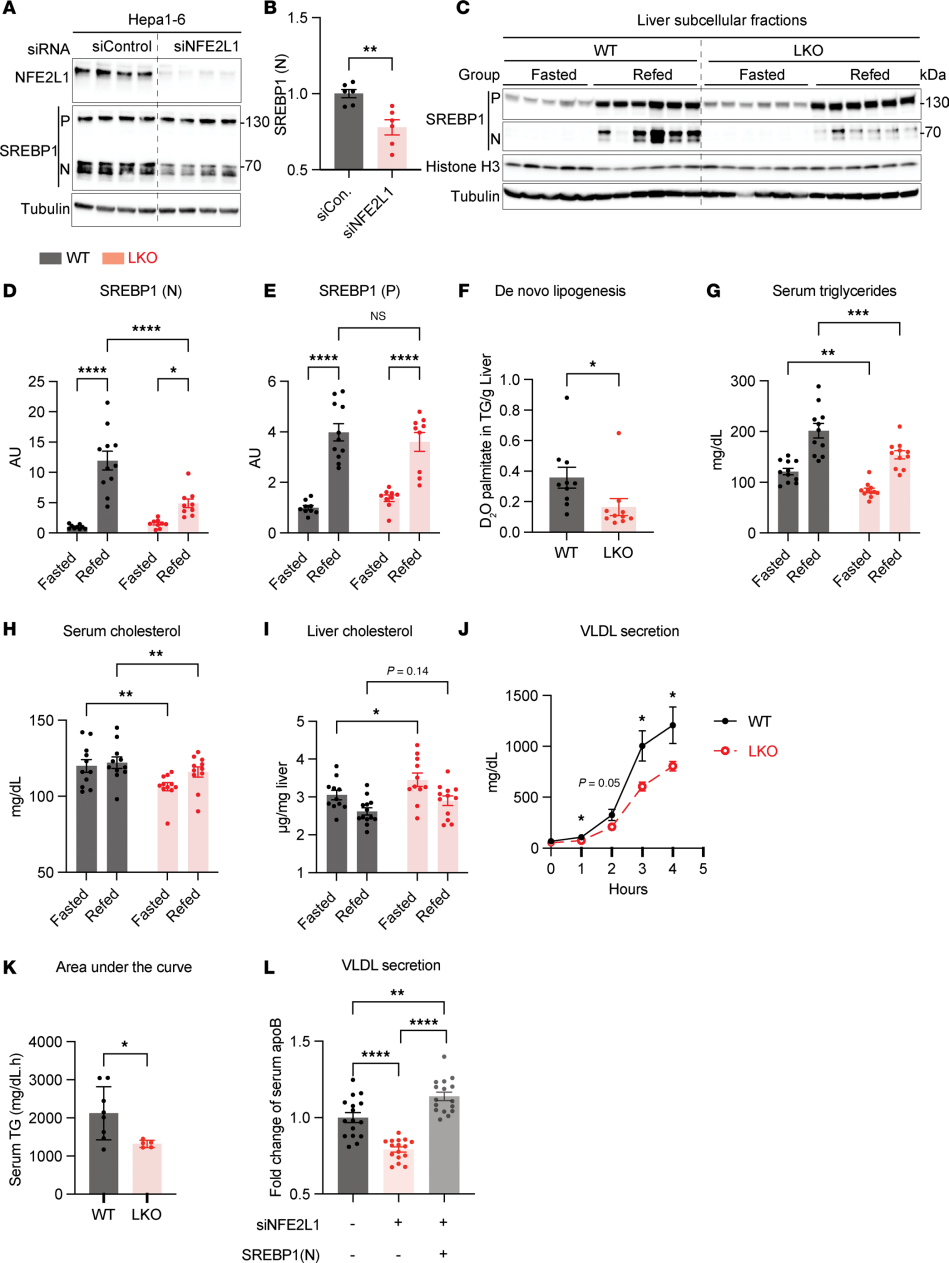
Hepatic NFE2L1 deficiency impairs SREBP1 activity, DNL, and VLDL secretion. (**A** and **B**) Immunoblot analysis and quantification of SREBP1 in Hepa1-6 hepatocytes after transient siRNA-mediated NFE2L1 knockdown (48 hours). siCon., control siRNA. (**C**) Subcellular fractionation of livers from 8-week-old liver-specific NFE2L-KO (LKO) mice and WT littermates under fasting or refed conditions (*n* = 9–11/group). Cytosolic and nuclear proteins were prepared from individual liver tissue sample fractions. Immunoblots show SREBP1(N) in the nuclear and SREBP1(P) in the cytosolic fractions. (**D** and **E**) Quantification of SREBP1(N) (normalized to histone H3) and SREBP1(P) (normalized to tubulin) from **C**. (**F**) DNL assay by D_2_O labeling in WT and LKO mice after overnight fasting followed by a 6-hour refeeding period. Hepatic TG–derived palmitate was analyzed for deuterium enrichment (*n* = 10/group). (**G** and **H**) Serum TG and total cholesterol levels in fasted and refed WT and LKO mice. (**I**) Liver total cholesterol in fasted and refed WT and LKO mice. (**J** and **K**) VLDL secretion: Plasma TG accumulation after tyloxapol injection in fasted WT and LKO mice (*n* = 5–8/group). (**L**) Quantification of apoB in media from Hepa1-6 cells after siRNA knockdown of NFE2L1 and transfection with either a control empty vector or nuclear SREBP1(N) expression constructs. Data represent the mean ± SEM. *P < 0.05, **P < 0.01, ***P < 0.001, and ****P < 0.0001, by 2-tailed, unpaired *t* test (**B**, **C**, **K**), 1-way ANOVA (**L**), or 2-way ANOVA (**D**, **E**, and **G**–**I**).

To investigate the potential link between NFE2L1 and SREBP1 activity, we transiently suppressed NFE2L1 in cultured mouse hepatoma cell line Hepa1-6 hepatocytes using an siRNA. Depletion of NFE2L1 selectively reduced the levels of the transcriptionally active form SREBP1(N) (65 kDa), while the precursor SREBP1(P) (130 kDa) remained unchanged (Figure 1, A and B). Endogenous SREBP2 protein levels could not be assessed because of the lack of a validated antibody for murine SREBP2. These results demonstrate that NFE2L1 may be required for normal SREBP1 activity in vitro.

We next tested whether hepatocyte-specific NFE2L1-knockout (LKO) mice exhibit defective SREBP1 processing in vivo. Since SREBP1 activation is dynamically regulated by nutritional status (17), WT and LKO mice were subjected to either overnight fasting (fasted) or fasting followed by a 6-hour refeeding period (refed). While LKO mice showed no differences in food intake, body weight, or serum levels of nonesterified fatty acids (NEFAs) (Supplemental Figure 1, A–C; supplemental material available online with this article; https://doi.org/10.1172/JCI197094DS1), subcellular fractionation of liver tissue revealed striking defects in SREBP1 activation. In the WT mice, refeeding robustly increased nuclear SREBP1(N) levels (65 kDa) compared with fasting, consistent with feeding-driven SREBP1 cleavage. In contrast, LKO exhibited more than a 50% reduction in nuclear SREBP1(N) under refed conditions (*P* < 0.0001 vs. refed WT; Figure 1, C and D), whereas the 130 kDa, ER membrane–bound precursor SREBP1(P) remained comparable between genotypes (Figure 1E). This indicates that NFE2L1 deficiency impaired proteolytic activation and nuclear translocation of SREBP1. Consistent with the reduced SREBP1(N), mRNA levels of the SREBP1/2 target genes *Ldlr*, *Hmgcr*, *Pcsk9*, *Fdps*, *Fasn*, *Acly*, and *Gck* were significantly downregulated in LKO mouse livers (Supplemental Figure 2, C–J). To functionally test the effect of these changes in vivo, deuterated water (D_2_O) labeling was performed after an overnight fast/refeed (6 hours; i.p. D_2_O, 27 μL/kg, with 4% D_2_O in drinking water), followed by gas chromatography–mass spectrometry (GC-MS) analysis of hepatic lipids. NFE2L1 LKO mice exhibited a significant reduction in deuterium incorporation into palmitate in hepatic TGs relative to WT controls (Figure 1F), indicating impaired de novo lipogenesis and functionally validating decreased SREBP1 activity in vivo. These results showed that NFE2L1 was essential for feeding-stimulated SREBP1 processing and subsequent transcriptional activation of the lipogenic program in the liver.

Given the central role of SREBP1 in lipid homeostasis, we next assessed systemic and hepatic lipids in the LKO mice. Liver-specific NFE2L1 deficiency reduced serum TG and total cholesterol levels in both the fasted and refed states (Figure 1, G and H), whereas liver TGs remained unchanged (Supplemental Figure 1D). However, hepatic cholesterol content was significantly elevated in the fasted LKO mice (Figure 1I), suggesting defective cholesterol export. To clarify whether altered LXR activity contributes to hepatic cholesterol accumulation in NFE2L1-deficient mice, we next analyzed this pathway. Examination of mRNA levels of the LXR target genes such as *Abca1*, *Cyp7a1*, and *Srebf1* showed no significant differences, *Abcg1* mRNA expression was increased (~140%), and *Abcg5* and *Abcg8* were modestly reduced (~30%) in LKO livers (Supplemental Figure 2, K–O). These modest and mixed effects indicate that LXR signaling remained largely unchanged, perhaps with only minor compensatory or secondary alterations. As noted, SREBP2 targets such as LDLR and HMGCR were downregulated in LKO livers (Supplemental Figure 2, C–E) despite elevated hepatic cholesterol levels, suggesting that, rather than increased synthesis or uptake, reduced VLDL secretion may be the primary cause of cholesterol retention. After fasting, most serum lipids are derived from hepatic VLDL secretion, hence, these results support the idea that VLDL biogenesis and secretion may be impaired in the livers of LKO mice. To directly test this, we blocked peripheral TG hydrolysis with tyloxapol (a lipoprotein lipase inhibitor) and quantified TG accumulation in the plasma over time, which reflects the rate of VLDL secretion (18). In WT mice, plasma TG rose linearly, reflecting robust VLDL secretion. In contrast, LKO exhibited a significant reduction in the TG accumulation rate (Figure 1, J and K), confirming defective VLDL secretion. To establish the requirement of SREBP1 for the effect of NFE2L1 on VLDL secretion, targeted loss- and gain-of-function studies were performed using apolipoprotein B (apoB) as a direct quantitative readout of VLDL export. Silencing NFE2L1 in Hepa1-6 cells caused reduced apoB release into conditioned media, which can be fully rescued by reexpression of the constitutively active nuclear SREBP1 in NFE2L1-depleted cells (Supplemental Figure 1E and Figure 1L), linking the secretory defect to reduced SREBP1 activity downstream of NFE2L1 deficiency. Together, these findings establish that NFE2L1 deficiency disrupted SREBP1-mediated VLDL secretion.

### NFE2L1 deficiency increases INSIG1 and suppresses SREBP1 activation.

**Figure 2:**
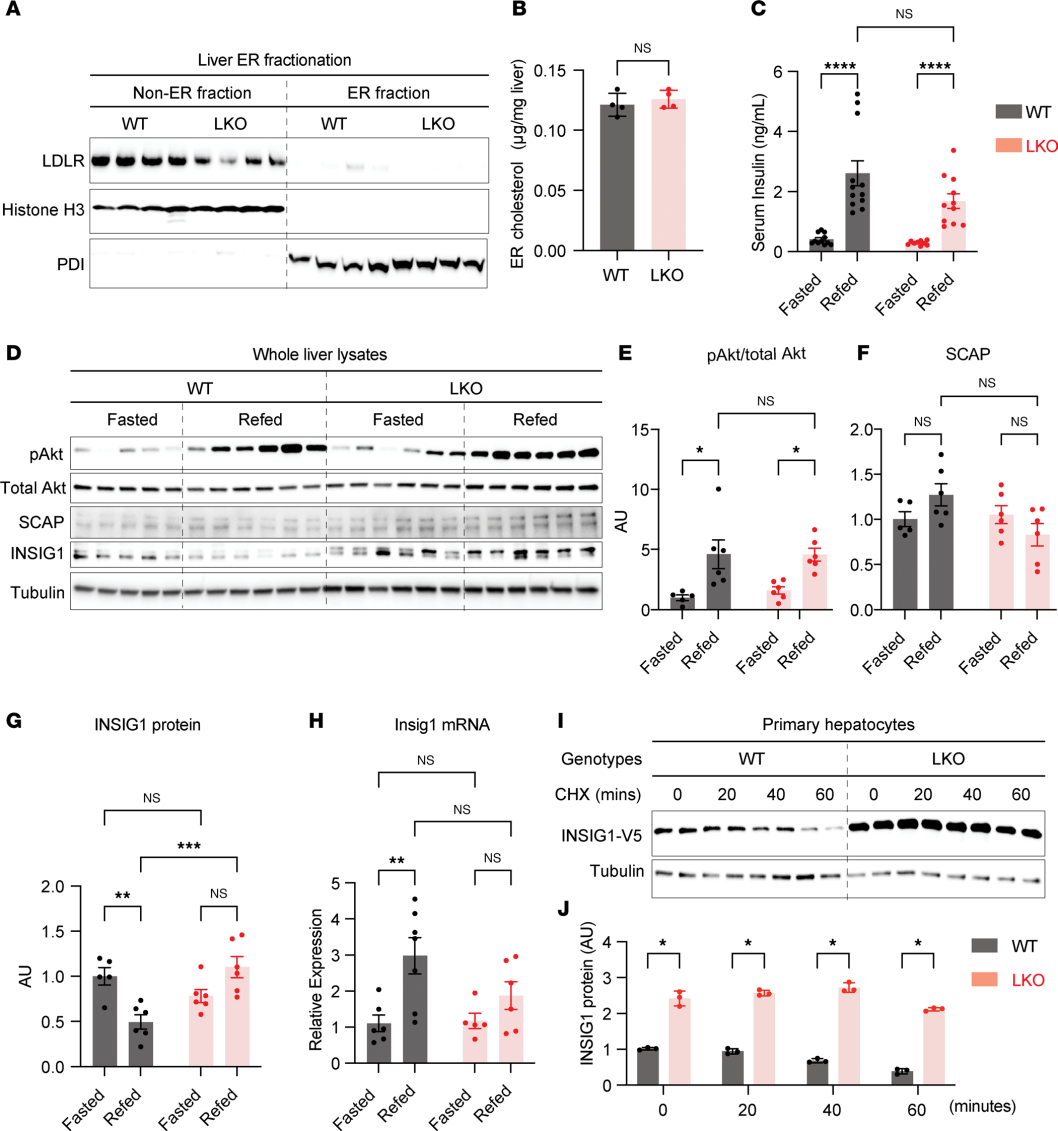
Hepatic NFE2L1 deficiency stabilizes INSIG1 protein. (**A**) Validation of the ER-enriched fraction in livers of refed WT and LKO mice (*n* = 4/group). Western blots were probed for subcellular markers: LDLR (plasma membrane), histone H3 (nuclear), and PDI (ER). (**B**) Cholesterol levels in the ER fractions isolated in **A**. (**C**) Serum insulin levels in fasted and refed WT and LKO mice (*n* = 9–12/group). (**D**) Immunoblot analysis of liver lysates from fasted and refed WT and LKO mice (*n* = 5–6/group). (**E**–**G**) Quantification of pAkt/total Akt, SCAP, and INSIG1 proteins normalized to tubulin in **D**. (**H**) qPCR analysis of *Insig1* mRNA in liver tissues. (**I** and **J**) CHX pulse-chase assay in primary hepatocytes isolated from WT and LKO mice expressing AAV-INSIG1-V5. Primary hepatocytes were treated with 10 μg/mL CHX for 0, 20, 40, or 60 minutes. Immunoblots are representative of 2 independent experiments (*n* = 3/condition). In graphs (**B**, **E**–**H**, and **J**), data for WT and LKO mice are shown with black and red bars, respectively. Data represent the mean ± SEM. *P < 0.05, **P < 0.01, ***P < 0.001, and ****P < 0.0001, by 2-tailed, unpaired *t* test (**B**) or 2-way ANOVA (**C** and **E**–**H**).

To elucidate how hepatic NFE2L1 regulates SREBP1, we next evaluated factors controlling SREBP1 nuclear translocation, including ER cholesterol content, insulin signaling, and SCAP and INSIG1 protein levels. We prepared ER membranes from liver tissue and validated the ER-enriched fraction, which revealed the presence of the ER-resident marker protein disulfide isomerase (PDI), whereas the plasma membrane marker LDL receptor (LDLR) and the nuclear marker histone H3 were undetectable, confirming the purity of the ER isolation (Figure 2A). The ER-enriched fractions from refed mice showed comparable cholesterol levels between WT and LKO mice (Figure 2B). Likewise, serum insulin levels, hepatic insulin signaling (as indicated by phosphorylated Akt [pAkt] levels), and SCAP protein levels were comparable between genotypes (Figure 2, C–F), making it unlikely that these factors were primary drivers of altered SREBP1 activity. Strikingly, fasting-refeeding regulation of INSIG1 protein was abolished in the LKO livers, with a 2-fold increase of endogenous INSIG1 protein in refed LKO livers (Figure 2G). Since INSIG1 is a known SREBP1 target, we assessed whether this upregulation was transcriptionally driven by measuring *Insig1* mRNA. Despite blunted induction of *Insig1* mRNA upon refeeding in LKO livers, *Insig1* mRNA levels did not differ significantly between refed WT and LKO livers (Figure 2H). This disconnection between mRNA and protein levels indicates that elevated INSIG1 protein in LKO livers is unlikely to be driven by transcriptional changes and that NFE2L1 may regulate INSIG1 protein stability.

To investigate how NFE2L1 affects INSIG1 stability, we expressed paramyxovirus SV5-tagged (V5-tagged) INSIG1 with a hepatocyte-specific thyroxine-binding globulin (TBG) promoter in the livers of NFE2L1-WT or -LKO mice using an adeno-associated virus (AAV) system. Three weeks after virus administration, primary hepatocytes were isolated, and the rate of INSIG1 degradation was monitored following cycloheximide (CHX) treatment. Notably, in the WT hepatocytes, a substantial fraction of INSIG1 disappeared within 60 minutes, reflecting a relatively short half-life of approximately 30 minutes (19). In contrast, in the NFE2L1-deficient hepatocytes, INSIG1 protein levels did not decrease and remained 2-fold higher than the WT levels after 60 minutes, indicating a markedly extended protein stability (Figure 2, I and J). To determine whether the effect of NFE2L1 on SREBP1 activity is dependent on INSIG1 in vivo, we next assessed exogenous INSIG1 levels in the liver. Consistently, we found that liver tissue from NFE2L1-LKO mice had significantly elevated INSIG1-V5 levels compared with WT controls under both fasting and refeeding conditions. Upon refeeding, the levels of SREBP1 were also reduced in the liver tissue from LKO mice (Supplemental Figure 3A). Taken together, these results suggest that liver NFE2L1 deficiency leads to increased hepatic INSIG1 levels, which in turn reduces SREBP1 activity, ultimately hindering VLDL biogenesis and secretion.

### NFE2L1 interacts with INSIG1 at the ER to facilitate INSIG1 degradation.

**Figure 3:**
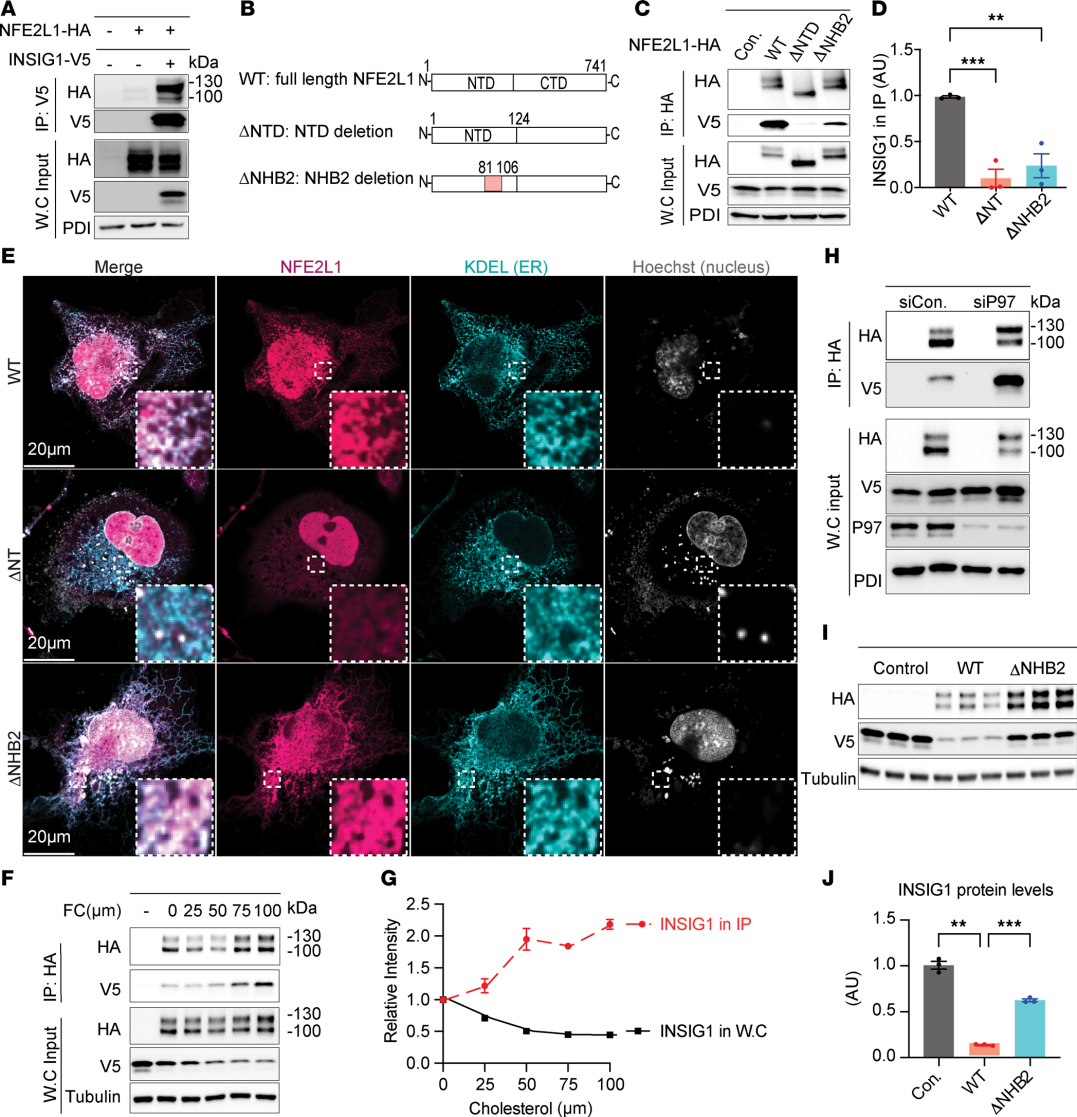
NFE2L1 interacts with INSIG1 in the ER through the NHB2 domain. (**A**) Immunoblot analysis of whole-cell (WC) lysates and anti-V5 immunoprecipitates from Hepa1-6 cells cotransfected with INSIG1-V5 and NFE2L1-HA constructs. (**B**) Schematic representation of NFE2L1 domain deletions (NTD and ΔNHB2, red) used for co-IP studies. (**C** and **D**) Co-IP of INSIG1-V5– and HA-tagged NFE2L1-WT or ΔNHB2 with anti-HA beads. Quantification of precipitated INSIG1-V5 signal normalized to IP NFE2L1-HA. In **D**, black, red, and blue bars represent NFE2L1-WT, ΔNTD, and ΔNHB2, respectively. (**E**) Confocal microscopy images of COS7 cells expressing HA-tagged NFE2L1-WT, ΔNTD, or ΔNHB2. Cells were stained for HA (NFE2L1, red), the ER marker KDEL (blue), and Hoechst nuclear dye. Magenta signals in the merged images indicate colocalization of NFE2L1 with the ER. Scale bars: 20 μm (main images); 2 μm (insets). (**F** and **G**) Co-IP of INSIG1 and NFE2L1 in Hepa1-6 cells treated with FC. Quantification of precipitated INSIG1-V5 signal normalized to IP NFE2L1-HA. (**H**) Co-IP of INSIG1-V5 and NFE2L1-HA in Hepa1-6 cells transfected with control or P97 siRNA. (**I** and **J**) Immunoblot analysis of INSIG1 in Hepa1-6 cells cotransfected with empty vector, HA-tagged NFE2L1-WT, or ΔNHB2 and INSIG1-V5. In **J**, black, red, and blue bars represent empty control, NFE2L1-WT, and ΔNHB2, respectively. All immunoblots are representative of 3 independent experiments. Data represent the mean ± SEM. **P < 0.01 and ***P < 0.001, by 1-way ANOVA (**D** and **J**).

We next explored how NFE2L1 regulates INSIG1 stability. NFE2L1, an ER membrane protein, cycles between the ER (as a 130 kDa glycosylated precursor) and the nucleus (as a proteolytically cleaved 100 kDa form) depending on the state of proteasome activity (15). Given their shared ER localization and sterol sensing ability, we hypothesized that NFE2L1 may regulate INSIG1 through a direct interaction. To address this possibility, we performed co-IP in Hepa1-6 cells coexpressing HA-tagged NFE2L1 and INSIG1-V5. We showed that V5-tagged INSIG1 specifically pulled down HA-tagged NFE2L1, with the 130 kDa ER-resident form as the predominating protein in the complex (Figure 3A). The amount of nuclear 100 kDa NFE2L1 was minimal in the immunoprecipitates, suggesting that the NFE2L1-INSIG1 interaction occurred predominantly in the ER.

To identify the NFE2L1 domain responsible for INSIG1 binding, we generated truncation mutants. Deleting the C-terminal domain (CTD) of NFE2L1 (residues 298–741) had no effect on INSIG1 binding, ruling out CTD involvement (Supplemental Figure 3, B and C). We then focused on the N-terminal domain (NTD) and engineered targeted deletions, including ΔNTD (lacking the entire N-terminal domain, residues 1–124, required for ER retention), ΔNHB1 (lacking the N-terminal homology box 1, residues 11–30, also required for ER retention), ΔSAS (lacking a signal peptide–associated sequence, residues 31–50), ΔCRAC1/2 (lacking the putative cholesterol recognition amino acid consensus sequences, residues 62–82), and ΔNHB2 (lacking the N-terminal homology box 2, residues 81–106) (20, 21) (Figure 3B and Supplemental Figure 3, D and E). Deletion of the NTD and the NHB2 within the NTD nearly abolished INSIG1 binding (Figure 3, C and D, and Supplemental Figure 3E). The mutant NFE2L1-ΔNTD showed a single band, whereas the ΔNHB2 protein, like NFE2L1-WT, exhibited 2 bands: 1 corresponding to the glycosylated precursor located in the ER and 1 corresponding to the cleaved form in the nucleus. As shown in previous studies, ΔNTD was predominantly located in the nucleus, whereas ΔNHB2 retained the same subcellular localization and transcriptional activity as NFE2L1-WT (22). Our confocal microscopy images further confirmed the subcellular location of NFE2L1-WT, -ΔNTD, and -ΔNHB2. Like NFE2L1-WT, ΔNHB2 was present in both the ER and the nucleus, whereas the majority of ΔNTD was mislocalized to the nucleus (Figure 3E). Despite its normal ER location, ΔNHB2 exhibited reduced binding to INSIG1, suggesting that the NHB2 domain within the NTD was required for their interaction.

Our previous study demonstrated that free cholesterol retains NFE2L1 in the ER (13). To test the effect of cholesterol on the regulation of the NFE2L1-INSIG1 interaction, we treated cells with various cholesterol derivatives: LDL, free cholesterol (FC), and 25-hydroxycholesterols (25-HC). Notably, only FC increased the amount of INSIG1 associated with NFE2L1 in the precipitates (Supplemental Figure 3F). To further validate this finding, we treated cells with increasing levels of cholesterol and observed that the ER-bound 130 kDa NFE2L1 increased progressively with increasing FC levels. Importantly, the amount of INSIG1 pulled down by NFE2L1 was also increased with rising levels of FC (Figure 3, F and G). To validate this regulation, we modulated NFE2L1 ER localization by silencing the valosin-containing protein (VCP) P97, an ATPase required for NFE2L1 retro-translocation to the cytosol. Knockdown of P97 increased amounts of the ER-localized NFE2L1, along with a corresponding increase in the INSIG1 co-immunoprecipitated by NFE2L1 (Figure 3H). These results further showed that NFE2L1 at the ER sensed free cholesterol levels and interacted with INSIG1 at this location.

Interestingly, the interaction inversely correlated with total INSIG1 levels: higher NFE2L1-INSIG1 binding (e.g., under FC treatment) coincided with lower INSIG1 in the whole cell (Figure 3, F and G, and Supplemental Figure 3F). To confirm causality, we coexpressed NFE2L1-WT, or the reduced INSIG1-binding mutant ΔNHB2, with INSIG1-V5. While NFE2L1-WT reduced INSIG1 protein levels by 90%, the INSIG1-binding–deficient mutant ΔNHB2 exhibited a significantly attenuated effect (Figure 3, I and J). This demonstrated that NHB2-mediated binding was essential for INSIG1 degradation. Altogether, these findings suggest that NFE2L1 interacts, via the NHB2 domain, with INSIG1 at the ER and, in response to excessive free cholesterol, to facilitate INSIG1 turnover.

To further explore the downstream turnover mechanism further, we mapped the INSIG1-NFE2L1 interaction interface using co-IP of transmembrane truncations of INSIG1: deletion of TM1-4 (ΔTM1-4) markedly reduced co-IP with NFE2L1, whereas deletion of TM4-6 had a lesser effect. These data suggest that TM1-4 harbors the primary binding region for NFE2L1, with potential auxiliary contacts in TM4-6 (Supplemental Figure 4, A–C). To define how NFE2L1 promotes INSIG1 turnover, we assessed ubiquitin dependence and proteasomal involvement. Proteasome inhibition (bortezomib) stabilized WT INSIG1, indicating partial reliance on the ubiquitin-proteasome system. An INSIG1 mutant replacing 3 lysine residues to arginine (INSIG1-3KR), designed to reduce ubiquitination, was also more stable than the WT, yet remained only partially sensitive to NFE2L1-mediated reduction, suggesting that a noncanonical mechanism may also contribute to INSIG1 degradation (Supplemental Figure 4D). In contrast, lysosomal blockade with bafilomycin had no effect on INSIG1 levels, excluding lysosomal degradation (Supplemental Figure 4E). Taken together, these data indicate that NFE2L1 may engage the TM1-4 region of INSIG1 and promotes its degradation primarily via the ubiquitin/proteasome pathway, with possible contributions from other noncanonical posttranslational mechanisms. The detailed biochemical coupling of NFE2L1 signaling to INSIG1 turnover will require additional future studies.

### The NFE2L1/INSIG1 axis drives SREBP1 activity and VLDL secretion.

To establish the physiological relevance of the NFE2L1-INSIG1 interaction, we performed in vivo rescue experiments in LKO mice using AAV vectors expressing NFE2L1-WT, or the ΔNHB2 mutant deficient in INSIG1 binding. NFE2L1 expression in the liver did not cause any significant alterations in body weight, food intake, liver weight, or serum levels of NEFA and insulin, as summarized in Supplemental Figure 5, A–E.

**Figure 4:**
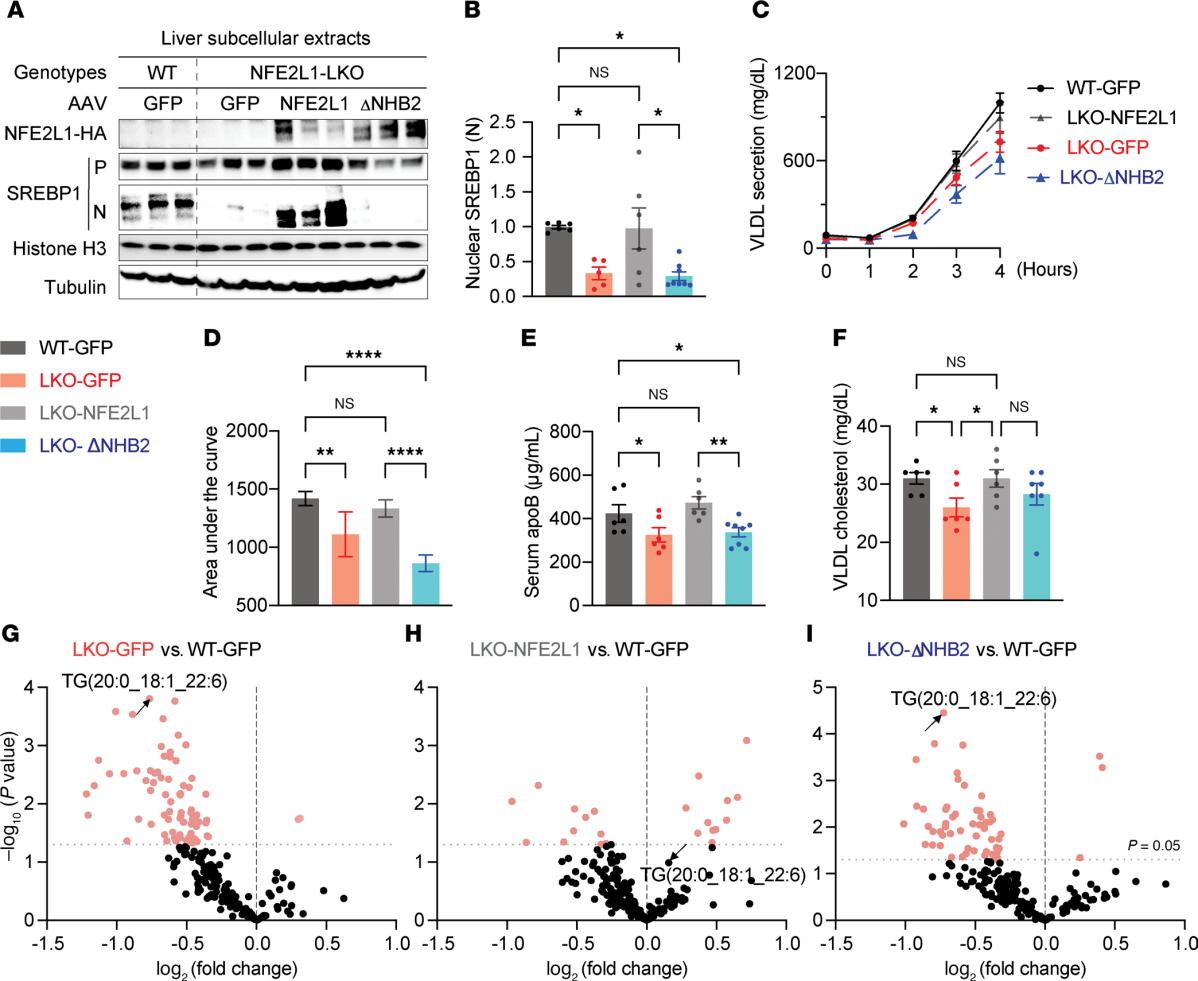
Hepatic NFE2L1/INSIG1 axis regulates SREBP1 and VLDL secretion. NFE2L1-LKO and WT littermates (8-week-old) were injected with GFP, NFE2L1-WT, or NFE2L1-ΔNHB2 containing AAV constructs. (**A**) Immunoblot analysis of SREBP1 protein in liver subcellular extracts. Mice were fasted overnight and refed for 6 hours prior to analysis (*n* = 5–8/group). Tubulin (cytosolic) and histone H3 (nuclear) served as loading controls. (**B**) Quantification of SREBP1(N) from **A**. (**C**) VLDL secretion assay: Mice were fasted overnight, and plasma TG levels were measured at 0, 1, 2, 3, and 4 hours after tyloxapol injection (*n* = 14/group). (**D**) AUC for TG secretion in **C**. (**E**) Fasted serum apoB lipoprotein levels (*n* = 6–8/group). (**F**) Fasted serum VLDL cholesterol (*n* = 6–8/group). (**G**–**I**) Lipidomics profiling for serum TG species (*n* = 6/group). Red denotes *P* < 0.05. Data represent the mean ± SEM. *P < 0.05, **P < 0.01, and ****P < 0.0001, by 1-way ANOVA (**B** and **D**–**F**).

We then fractionated the liver into cytoplasmic and nuclear fractions to compare the protein levels of precursor membrane-bound SREBP1(P) and the active nuclear form SREBP1(N). NFE2L1-LKO mice in the AAV-GFP–expressing (LKO-GFP–expressing) group exhibited significantly lower levels of nuclear SREBP1(N) compared with WT mice (WT-GFP) (Figure 4, A and B). Importantly, reintroducing NFE2L1-WT into LKO livers (LKO-NFE2L1) restored nuclear SREBP1(N) levels that were comparable to those in WT mice. Conversely, expression of the ΔNHB2 mutant deficient in INSIG1 binding (LKO-ΔNHB2) resulted in SREBP1(N) levels similar to those in the LKO-GFP group, which were significantly lower than those in the WT-GFP and LKO-NFE2L1 mice (Figure 4, A and B). Consistently, mRNA expression of the SREBP target genes *Hmgcs*, *Hmgcr*, *Mvd*, *Mvk*, and *Pcsk9* were markedly diminished in LKO-GFP mice compared with their WT counterparts. Restoration of NFE2L1-WT, but not ΔNHB2, expression in LKO mice led to the normalization of gene expression (Supplemental Figure 6, A–G). Importantly, mRNA levels of the established NFE2L1 target gene *Psmb5* were comparable between the NFE2L1-WT and ΔNHB2 mice (Supplemental Figure 6H), suggesting that the observed difference in SREBP1 activity is attributable to the interaction between NFE2L1 and INSIG1 rather than to differences in general transcriptional activity.

To test whether the NFE2L1/INSIG1 axis promotes VLDL secretion in vivo, we performed a secretion assay in the LKO mice expressing NFE2L1-WT and NFE2L1-ΔNHB2. Consistent with the reduction of SREBP1 (8), NFE2L1-LKO mice exhibited significantly reduced hepatic VLDL secretion compared with the WT controls (Figure 1, I and J and Figure 4, C and D). Notably, reintroduction of NFE2L1-WT into the LKO mice restored VLDL secretion, whereas expression of the mutant NFE2L1-ΔNHB2 form (which does not interact with INSIG1) failed to correct VLDL secretion in the NFE2L1-LKO mice (Figure 4, C and D). This observation was further supported by similar patterns detected in the regulation of serum apoB and VLDL cholesterol levels measured in the fasted state (Figure 4, E and F). These results underscore the importance of the direct interaction between NFE2L1 and INSIG1 in modulating VLDL biogenesis and secretion in vivo.

To gain further insights into the effect of the NFE2L1/INSIG1 axis on lipid metabolism, we performed lipidomics analysis to determine serum lipid composition in detail. Lipid profiles revealed that NFE2L1 deficiency significantly reduced total TGs and diminished most individual TG species, irrespective of their fatty acid composition (Figure 4G and Supplemental Table 1). Strikingly, while reexpression of NFE2L1-WT restored serum TG abundance and species in LKO mice (Figure 4H), the ΔNHB2 mutant deficient in INSIG1 binding failed to rescue this phenotype (Figure 4I). For instance, TGs (20:0_18:1_22:6), which were significantly reduced in LKO mice compared with WT controls, returned to WT levels upon NFE2L1-WT reexpression but remained suppressed in ΔNHB2-expressing mice (Figure 4, G–I). This regulatory effect extended to phosphatidylcholine (PC) species, which are essential for VLDL assembly and secretion. PC levels, which were significantly reduced in LKO mice, were partially rescued by NFE2L1-WT, but not by the ΔNHB2 mutant (Supplemental Figure 7, A–C). These results support the necessity of intact NFE2L1-INSIG1 interaction in maintaining hepatic and systemic lipid homeostasis, with the ΔNHB2 mutant’s inability to restore TG or PC profiles highlighting the critical role of this interaction in coordinating lipogenesis and VLDL secretion.

Parallel lipidomics profiling of the TG pool demonstrated that the NFE2L1/INSIG1 axis selectively modulated polyunsaturated fatty acid (PUFA) composition. Notably, docosahexaenoic acid (DHA, 22:6), which was depleted in LKO mice, was fully restored by NFE2L1-WT but not by the ΔNHB2 mutant (Supplemental Figure 7, D–F, and Supplemental Table 2). In addition to DHA, we also detected similar patterns of regulation for both docosapentaenoic acid (DPA, 22:5), and eicosapentaenoic acid (EPA, 20:5), indicating that antiinflammatory PUFAs may be preferentially regulated by the NFE2L1/INSIG1 axis (Supplemental Figure 7, G–I). Interestingly, clinical data also show that elevated PUFAs correlate with reduced MASH severity by enhancing VLDL secretion, suppressing liver inflammation and inhibiting hepatic stellate cell (HSC) fibrogenesis (23, 24). These results suggest that modulation of the NFE2L1/INSIG1 axis may counteract important pathways in MASH pathogenesis, a mechanism we directly tested in our in vivo model.

### The NFE2L1/INSIG1 axis enhances hepatic cholesterol secretion and attenuates MASH.

NFE2L1-deficient mice exhibited liver dysfunction even on a standard chow diet, which was characterized by 2.5-fold elevated serum alanine transaminase (ALT) and 1.2-fold higher aspartate transaminase (AST) (Supplemental Figure 9A), biomarkers indicative of hepatocellular injury. In addition, the LKO mice exhibited significant upregulation of genes associated with liver inflammation (e.g., *Cd68*, *Ccl2*, and *Ccl5*) and fibrosis (e.g., *Tgfb*, *Col1a1*, and *Timp1*) (Supplemental Figure 9B). These findings highlight the critical role of NFE2L1 in maintaining liver health and are consistent with earlier reports (13, 15, 16, 25, 26). To understand the involvement of NFE2L1 under high-fat diet–induced (HFD-induced) liver pathophysiology, we performed a preliminary 2-month HFD study comparing WT and NFE2L1-LKO mice. NFE2L1-deficient mice displayed no significant differences in liver weight, serum lipid levels, lipogenic or inflammatory/fibrotic gene expression, or serum ALT/AST levels (Supplemental Figure 8, A–D) compared with WT control mice. Histology showed comparable steatosis and inflammation and similar amounts of hepatic lipid content between genotypes (Supplemental Figure 8, E–G). These findings indicate that NFE2L1 deficiency did not markedly influence hepatic responses to a HFD, which was primarily enriched in fatty acids in this setting. Consistent with our previous work, the most pronounced effects of NFE2L1 loss occur under cholesterol stress, reinforcing the idea that NFE2L1 functions as a cholesterol-responsive regulator rather than a general mediator of TG accumulation.

**Figure 5:**
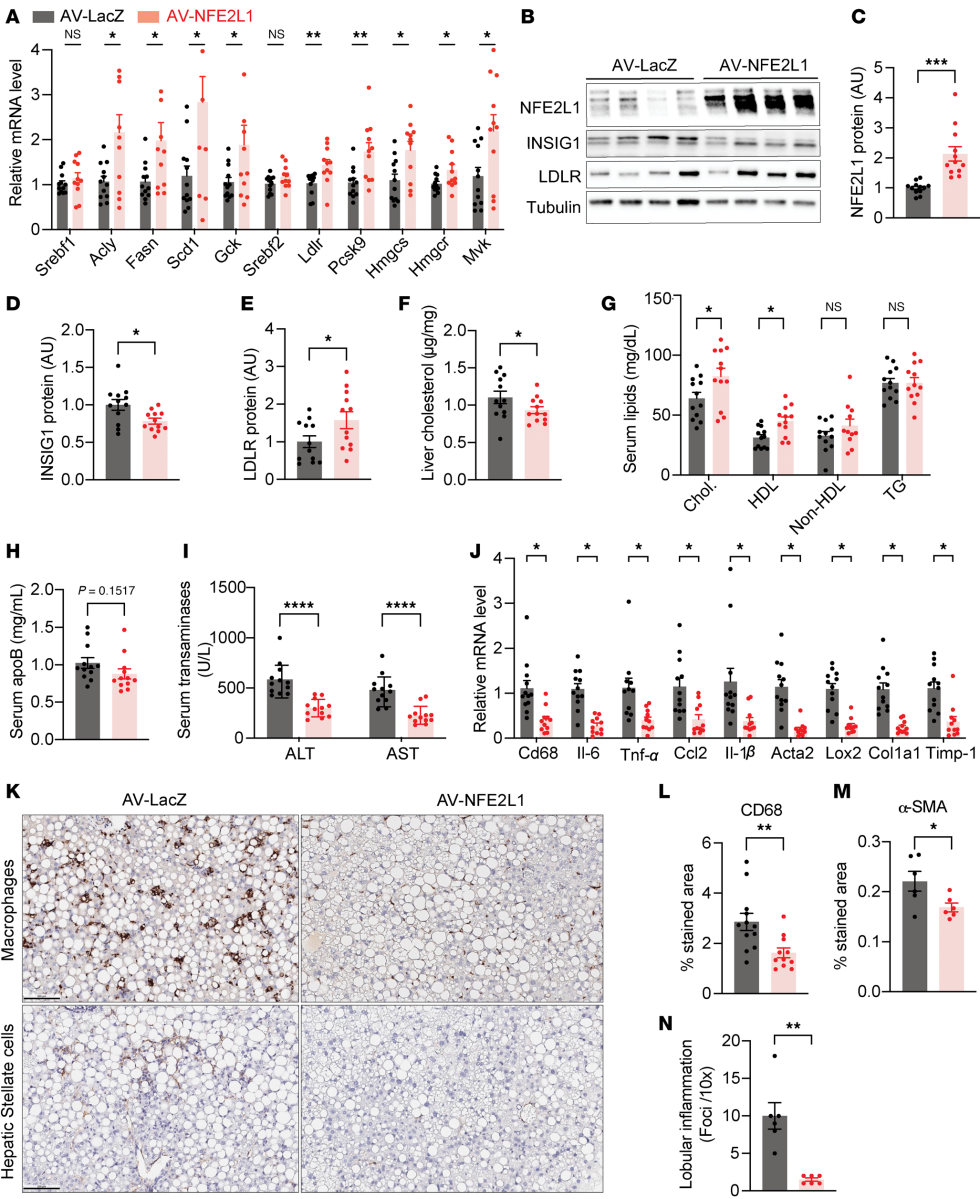
NFE2L1 ameliorates liver injury and inflammation in MASH. Eight-week-old *db/db* mice were injected with either AV-LacZ or AV-NFE2L1 and fed a MCD diet for 2 weeks. Mice were fed ad libitum before sample collection (*n* = 12/group). (**A**) Liver mRNA levels of SREBP1/2 target genes normalized to 18S ribosomal RNA (18S). (**B**–**E**) Immunoblot analysis and quantification of NFE2L1, INSIG1, and LDLR in liver lysates. (**F**) Liver cholesterol levels. (**G**) Serum lipid profiles measured via Piccolo Lipid Panel Plus. (**H**) Serum apoB levels by ELISA. (**I**) Serum ALT and AST levels (Piccolo Lipid Panel Plus). (**J**) mRNA levels of liver inflammation and fibrosis markers. (**K**–**N**) Representative histology images (scale bars: 200 μm) (**K**), quantification of liver section staining for the macrophage marker CD68 (**L**) and the hepatic stellate cell marker α-SMA (**M**), and lobular inflammation (**N**). Data represent the mean ± SEM. *P < 0.05, **P < 0.01, ***P < 0.00, and ****P < 0.0001, by 2-tailed, unpaired *t* test.

To directly evaluate NFE2L1’s therapeutic potential in MASH, we used *db/db* mice that were fed a methionine/choline-deficient (MCD) diet, a mouse model of severe MASH characterized by impaired VLDL secretion and progressive liver damage (27, 28). We first tested hepatocyte-specific NFE2L1 overexpression in the *db/db* MCD model using AAV8-TBG-NFE2L1. Despite a dose escalation, we were not able to achieve sufficient NFE2L1 expression within the 2-week treatment window. We also tested nonparenchymal contributions, including Kupffer cell–specific (Nfe2l1-Clec4f-Cre [KKO]) and myeloid-specific NFE2L1 (Nfe2l1-LysM-Cre [MKO]) deletion models in mice. Neither model showed significant changes in serum ALT or AST levels, serum TGs, cholesterol, or VLDL (Supplemental Figure 9, C–F). In addition, AAV-TBG-NFE2L1 delivery to chow-fed LKO mice restored hepatic NFE2L1 expression and normalized serum ALT and AST elevations (Supplemental Figure 9, G and H). These results confirm that the hepatocyte compartment was likely the major locus responsible for the metabolic and injury phenotypes associated with NFE2L1 deficiency. Therefore, we decided to administer adenoviral vectors (AVs) expressing either LacZ as a control or NFE2L1 to mice being fed a MCD diet for 2 weeks. Overexpression of NFE2L1 robustly activated the SREBP pathway, increasing the hepatic mRNA levels of lipogenic targets (Figure 5A). Immunoblot analysis confirmed the increase in NFE2L1 protein, accompanied by a significant reduction in INSIG1 levels (Figure 5, B–D), which facilitated an increase in SREBP1/2 activity. This was further supported by the upregulation of LDLR protein (Figure 5E), a key SREBP2 target critical for LDL cholesterol clearance.

Notably, in mice expressing NFE2L1, we observed reduced liver cholesterol but elevated serum total cholesterol levels compared with controls (Figure 5, F and G). To understand these changes in cholesterol metabolism, we examined *Abca1* gene expression and found no significant difference between control and NFE2L1-overexpressing groups. Similarly, mRNA levels of other LXR-responsive genes (*Abcg5*, *Abcg8*, and *Cyp7a1*) were unchanged, indicating that the LXR pathway remained inactive under these conditions (Supplemental Figure 10). This indicated enhanced VLDL-mediated export, suggesting that NFE2L1 overexpression shifted cholesterol flux from hepatic retention to secretion. Importantly, this serum cholesterol increase was redistributed into anti-atherogenic HDL, while atherogenic risk markers such as non-HDL cholesterol (LDL/chylomicron remnants) and apoB remained unchanged (Figure 5, G and H), probably due to compensatory LDLR-mediated clearance of atherogenic particles.

The metabolic benefits of NFE2L1 extended to ameliorating the MASH-associated pathology. Notably, these mice displayed a significant reduction in serum ALT and AST levels, suggesting that NFE2L1 expression prevented hepatic injury (Figure 5I). Hepatic expression of inflammation and fibrosis markers, including *Cd68*, *Il6*, *Tnfa*, *Ccl2*, *Il1b*, *Acta2*, *Lox2*, *Col1a1*, and *Timp1*, were significantly decreased in the livers of NFE2L1-expressing mice compared with the controls (Figure 5J), consistent with attenuation of inflammation and stellate cell activation. Histopathological analysis corroborated these findings: CD68^+^ macrophage infiltration was reduced by 45%, and α smooth muscle actin–activated (α-SMA–activated) stellate cells diminished by 24% in the liver tissue upon NFE2L1 expression (Figure 5, K–M). These histological observations, in addition to decreased lobular inflammation (Figure 5N), were independently confirmed in a blinded manner by 2 pathologists, further validating that NFE2L1 overexpression led to a significant reduction in inflammation and fibrosis in this model of MASH. Our results indicate that NFE2L1 reduced macrophage infiltration in the liver and decreased the activation of HSCs, which are the hallmarks of liver inflammation and fibrosis. These observations suggest potential pathways for targeting NFE2L1 to mitigate MASH pathogenesis.

## Discussion

Our findings reveal what we believe to be a previously unrecognized mechanism by which NFE2L1 maintains hepatic lipid and cholesterol homeostasis through its interaction with INSIG1, acting in parallel with the canonical SCAP/SREBP signaling and VLDL export. We demonstrate that NFE2L1 deficiency elevated INSIG1 levels, suppressing SREBP1 activation and impairing VLDL secretion, which drove hepatic cholesterol accumulation and spontaneous liver injury, whereas NFE2L1 overexpression in *db/db* mice fed a MCD diet increased SREBP1/2 activity and VLDL-mediated lipid export and attenuated MASH hallmarks: liver injury, inflammation, and fibrosis. Central to this regulatory interaction is the NHB2 domain of NFE2L1, which directly binds and destabilizes INSIG1 in the ER to enable SREBP1 processing and activation. Rescue experiments confirm the functional importance of this interaction: NFE2L1-WT restored lipid homeostasis in KO mice, whereas the ΔNHB2 mutant (deficient in INSIG1 binding) failed to rescue SREBP1 activation or VLDL secretion. Lipidomics profiling further illustrated the specificity of this axis, whereby NFE2L1-WT, but not ΔNHB2, restored serum TGs and fatty acid composition, notably restoring antiinflammatory PUFAs such as DHA and EPA.

The interaction between NFE2L1 and INSIG1 is dynamically modulated by FC levels, enabling NFE2L1 to calibrate its interaction with SREBP according to the ER lipid status. This creates a feedback loop, in which NFE2L1 licenses SREBP activation under cholesterol-replete conditions while simultaneously coupling it to VLDL-mediated cholesterol export, a “lipid release valve” mechanism that prevents lipotoxicity by balancing lipogenesis with secretion. Such coordination reconciles the dual roles of SREBP in lipid metabolism: while chronic overactivation is often linked to steatosis, its downregulation impairs VLDL secretion and exacerbates MASH (29, 30), highlighting the necessity of balanced SREBP activity. It is important to note that although SREBP activation is generally viewed as prosteatotic, its functional consequences are context dependent. In our study, NFE2L1-mediated INSIG1 degradation occurred specifically under conditions of cholesterol excess, where the primary consequence was to restore VLDL export capacity rather than to drive de novo lipogenesis. This was directly supported by our finding that NFE2L1 overexpression activated SREBP1/2 while simultaneously reducing hepatic cholesterol and attenuating liver injury, inflammation, and fibrosis (Figure 5, B–N). Thus, under conditions of sterol overload, the NFE2L1/INSIG1 axis appeared to redirect SREBP activity toward lipoprotein secretion, consistent with prior evidence that impaired VLDL secretion, rather than excess lipogenesis, can be the primary driver of hepatic cholesterol retention and MASH progression (29, 30). Our work aligns with evidence that context-specific SREBP activation can optimize lipid dynamics and that it partners with NFE2L1 in this response. For example, INSIG1 suppression in mice enhances SREBP activity, but reduces hepatic lipid retention by coupling lipogenesis to secretion, resulting in improving liver function and reducing inflammation (31). Similarly, statins, which indirectly activate SREBP2, improve MASH outcomes in humans (32, 33), mirroring our observation that NFE2L1-driven SREBP activation alleviated liver disease.

NFE2L1 overexpression in *db/db* mice reduced hepatic cholesterol while increasing serum cholesterol via enhanced VLDL secretion, attenuating inflammation and fibrosis. Importantly, this intervention did not elevate atherogenic apoB or non-HDL levels, likely due to compensatory LDLR upregulation, a safety feature reminiscent of statin effects. These findings may suggest potential translational opportunities to combat MASH, a disease affecting 5% of the global population with limited treatments (34, 35). However, broader applicability of the in vivo function of NFE2L1 must be validated in diverse models, such as a high-fat, high-cholesterol diet– or toxin-induced MASH, as well as additional models of NFE2L1 expression and/or activation.

While NFE2L1 is established as a transcription factor regulating proteasome expression and antioxidative responses (36, 37), our present work uncovers a role for its ER-resident form in sensing cholesterol to promote lipid secretion. By balancing lipogenesis with VLDL-driven secretion, NFE2L1 ensures that lipid demands are met without compromising cellular resilience. NFE2L1’s preferential elevation of HDL-associated cholesterol and antiinflammatory PUFAs may suggest a favorable cardiovascular safety profile associated with the mechanism described in this study. This aligns with prior studies showing that SREBP1-mediated lipogenesis supplies essential substrates essential for VLDL assembly (5, 9), yet underscores the need for rigorous cardiovascular risk assessment in future studies. Testing NFE2L1 activation in atherosclerosis-prone models would be an important path to further clarify its effect on vascular health while reinforcing its role in hepatic protection. Several limitations of this study should be acknowledged. Our in vivo therapeutic experiments were performed in *db/db* mice, a leptin receptor-deficient model that does not fully recapitulate the pathophysiology of most cases of MASH in humans. While this model is useful for studying hepatic cholesterol dysregulation in a metabolically stressed background, extending these findings to diet-induced MASH models in WT mice will be important to confirm the broader generalizability of NFE2L1/INSIG1 axis modulation. The relevance of these findings to female animals and human MASH in both sexes, as well as the effect of hormonal or dietary modulation of this axis, remains to be tested. The detailed mechanism underlying NFE2L1-mediated INSIG1 degradation, including the identification of the responsible E3 ligase and regulatory cofactors, is currently not known and warrants further investigation. It will also be informative to determine to what extent the activity of SREBP mediates NFE2L1’s effect in vivo.

Our work defines NFE2L1 as a guardian of hepatic metabolic flexibility, orchestrating SREBP activity through INSIG1 destabilization to balance lipid synthesis with secretion. By resolving the paradox of SREBP’s dual roles and the interactions with NFE2L1, our findings advance a mechanism whereby coordinated activation of lipid pathways mitigate MASH without exacerbating systemic risk. Future efforts to modulate the NFE2L1/INSIG1 axis may offer strategies for the treatment of metabolic liver disease, addressing a critical unmet need.

## Methods

### Sex as a biological variable.

All animal studies used male mice, as the *db/db* MASH model and the liver-specific Nfe2l1-KO model produce robust and reproducible metabolic and hepatic phenotypes in male mice under the dietary and housing conditions used in this study. Although the present study was conducted in 1 sex, we expect that the core mechanism linking NFE2L1, INSIG1, cholesterol handling, and VLDL secretion is likely relevant to both sexes; this will require direct investigation in female mice in future studies.

### Cell lines and cell culture conditions.

Murine Hepa1-6 cells were cultured in DMEM supplemented with 5% Cosmic Calf Serum (CCS) (Cytiva). Other cell culture media conditions are described below. All cells were cultured at 37°C in a humidified incubator maintained at a CO_2_ level of 10%. For gene-silencing experiments, Hepa1-6 cells were transfected with specific siRNAs targeting NFE2L1 using Lipofectamine RNAiMAX (Thermo Fisher Scientific) according to the manufacturer’s instructions. A control siRNA (nontargeting) was used as a negative control. Briefly, 1 × 10^5^ cells were seeded in 6-well plates 24 hours prior to transfection. The siRNA and Lipofectamine RNAiMAX reagent were incubated in Opti-MEM medium (Thermo Fisher Scientific) for 15 minutes before being added to the cells. After 48 hours of transfection, cells were harvested for further analyses including Western blotting, quantitative PCR (qPCR), and co-IP experiments. For primary mouse hepatocyte isolation, mice were anesthetized with 100 mg/kg ketamine and 10 mg/kg xylazine, and the liver was perfused with warm PBS containing 0.5 mM EGTA, followed by digestion with a collagenase solution (collagenase type IV, MilliporeSigma) for 15–20 minutes at 37°C. The digested liver was then minced and filtered through a 100 μm cell strainer to obtain a single-cell suspension. Hepatocytes were plated in collagen-coated culture plates at a density of 1 × 10^6^ cells per well in Williams’ E medium (Thermo Fisher Scientific) supplemented with 10% FBS and 1% penicillin-streptomycin. Cells were cultured at 37°C in a humidified incubator at 5% CO_2_. Hepatocytes were used within 24 hours of isolation.

### Cloning and mutagenesis to generate mutant forms of NFE2L1.

To generate the NFE2L1 mutants, site-directed mutagenesis was performed using the Q5 Site-Directed Mutagenesis Kit (New England BioLabs) according to the manufacturer’s instructions. Briefly, the full-length NFE2L1 cDNA was subcloned into the pcDNA5.0 vector using standard cloning methods. The NFE2L1-WT construct was used as the template for generating the mutants. For NFE2L1-ΔNHB2, a specific mutation was introduced to delete the NHB2 domain (amino acids 81–106) by introducing a stop codon. This was achieved using the forward primer (CGAGACCCGGAGGGGTCT) and the reverse primer (CCGGGCAGTGAAGTAATTGTCC). The mutated construct was verified by Sanger sequencing to confirm deletion of the NHB2 domain. The NFE2L1-WT and NFE2L1-ΔNHB2 plasmids were subsequently used for transfection into Hepa1-6 cells for functional analysis, including protein expression and co-IP.

### IP and immunoblotting experiments.

Tissues and cells used for immunoblotting experiments were lysed in buffer containing 150 mM NaCl, 50 mM Tris-HCl (pH 7.4), 1% v/v Nonidet P-40, 5 mM EDTA, and fresh protease inhibitors. Lysates were incubated on ice for 30 minutes and then centrifuged at 4°C for 15 minutes at 13,000*g*, and the supernatant was transferred to a fresh tube. Protein concentrations were determined using the Pierce BCA Protein Assay kit (Thermo Fisher Scientific), and samples were stored in Laemmli buffer at –20°C until analysis. Electrophoresis of protein was run at neutral pH in 4%–20% Criterion TGX Stain-Free Protein Gel (Bio-Rad). Proteins in gels were transferred to nitrocellulose membrane, blocked in TBS-T containing 1% milk, and then incubated overnight with primary antibodies in TBS-T containing 1% milk at 4°C with shaking. After washing, anti-rabbit or anti-mouse HRP-conjugated antibodies were applied for 30 minutes at room temperature. Membranes were incubated with Super Signal West Femto (Thermo Fisher Scientific) to generate a chemiluminescent signal, and images were captured using Image Lab software (Bio-Rad).

### Cholesterol treatments in cultured cells.

We first solubilized dry cholesterol powder in 100% ethanol at 65°C and then added this to methyl-β-cyclodextrin (MβCD) in water at a concentration of 5 mM cholesterol to 42 mg/mL MβCD, which was then cooled and sterile filtered. Cells were treated by adding the cholesterol complex to media at a concentration of 50 μM (or otherwise at the indicated doses) for 3 hours. This method causes cells to accumulate cholesterol because the stoichiometric capacity of MβCD to complex with cholesterol is near maximal. As such, in cell culture media, there is a high tendency for cholesterol to transfer into cellular membranes, at which point it circulates through the normal cellular cholesterol pools.

### SREBP1(N) rescue in NFE2L1-silenced hepatocytes.

Hepa1-6 cells were transfected with either Nfe2l1-targeting or nontargeting control siRNA and incubated for 24 hours to achieve knockdown, followed by transfection with a constitutively active nuclear SREBP1 expression construct, SREBP1(N), with an STII tag or an empty vector control; cells were harvested 24 hours later to verify SREBP1(N) expression by immunoblotting and, in parallel, switched to secretion medium for 24 hours to quantify apoB in conditioned media by ELISA as a readout of VLDL assembly and export.

### Immunofluorescence analysis.

COS-7 cells transiently transfected with WT or mutant variants of HA-tagged NFE2L1 were seeded on poly-l-lysine–coated coverslips. After a 2-hour treatment with the indicated chemicals, cells were fixed with 3% paraformaldehyde for 15 minutes, washed twice in PBS, permeabilized with 0.1% Triton X-100 in PBS (Triton X-100/PBS) for 15 minutes, and then washed twice in PBS. Antibodies were diluted in blocking buffer plus 0.05% Triton X-100. Primary antibody immunodetection was performed overnight at 4°C, followed by 2 Triton X-100/PBS washes. Secondary antibody incubation was performed at room temperature for 1 hour, followed by 2 Triton X-100/PBS washes and 2 PBS washes. Preparations were further incubated with DAPI in PBS (1/10,000) for 15 minutes, followed by 2 additional PBS washes. Images were taken in our imaging lab using a Leica SP8 X confocal microscope equipped with 405 nm and white light lasers and a 63× oil immersion objective.

### Subcellular fractionation of liver tissues.

Liver tissues from mice were processed for subcellular fractionation using Thermo Fisher Scientific’s NE-PER Nuclear and Cytoplasmic Extraction system following the manufacturer’s instructions. Briefly, livers were harvested and immediately placed in cold PBS. The tissue was then homogenized in the cytoplasmic extraction buffer, supplemented with protease inhibitors. The homogenates were centrifuged at 1,000*g* for 10 minutes at 4°C to remove debris, and the supernatant was collected as the cytoplasmic fraction. For nuclear extraction, the remaining pellet was resuspended in the nuclear extraction buffer, incubated on ice for 30 minutes, and then centrifuged at 14,000*g* for 10 minutes at 4°C to separate the nuclear fraction. The supernatant was discarded, and the nuclear pellet was resuspended for subsequent analysis. Both the nuclear and cytoplasmic fractions were stored at –80°C until used for protein quantification and Western blot analysis. Protein concentrations were determined using the Pierce BCA protein assay kit (Thermo Fisher Scientific) according to the manufacturer’s instructions, and equivalent amounts of protein from both fractions were used for analysis of target proteins by Western blotting.

### qPCR.

Cells or tissues were homogenized in TRIzol Reagent for total RNA isolation. cDNA was synthesized iScript cDNA Synthesis Kit. qPCR was performed on a ViiA7 system (Applied Biosystems) using SYBR green. Gene of interest cycle thresholds (Cts) were normalized to 18S levels by the DDCt method and displayed as expression levels relative to controls.

### Mouse studies.

All mice were bred and housed under a 12 hour light/12-hour dark cycle in the Harvard T.H. Chan School of Public Health pathogen-free barrier facility with ad libitum access to a standard chow diet (5053, PicoLab Mouse Diet from LabDiet), unless otherwise specified. Male mice were used for all experiments, which were performed at the using these mice at the age of 8–10 weeks. Liver-specific, Nfe2l1-deficient (NFE2L1-LKO) mice were generated by crossing Nfe2l1-floxed mice with hemizygous B6.Cg-Tg (Alb-Cre) 21Mgn/J-transgenic mice expressing albumin promoter driven Cre recombinase. Nfe2l1-floxed mice were generated as described previously from embryonic stem cells carrying the Nfe2l1^tm1a(EUCOMM)Hmgu^ allele. Genotyping for the Nfe2l1 flox and Cre recombinase was performed by real-time PCR with specific probes designed for each gene (Transnetyx). Mice were injected i.v. via the retro-orbital plexus with 1 × 10^11^ viral genomes of AAV-NFE2L1-WT, AAV-NFE2L1-ΔNHB2, or AAV-GFP. Three weeks after injection, mice were subjected to fasting (16 hours) followed by refeeding (6 hours). Mice were then euthanized, and tissues and serum were collected for further analysis. Eight-week-old male *db/db* mice were placed under isoflurane anesthesia (1%–3%) and dosed i.v. via the retro-orbital plexus with 1 × 10^9^ particles/mouse (AV-LacZ, AV-NFE2L1, Vector Biolabs) on day 0. On day 2 after virus injection, mice were started on a methionine and choline-deficient (MCD) diet (A02082002BR, Research Diets). Mice were euthanized on day 14. For high-fat diet studies, mice were placed on a diet of 60% kcal from fat (D12492, Research Diets) at 8 weeks of age and maintained until euthanasia at 16 weeks, when serum and tissues were collected for analysis.

### Serum NEFA, TG, and insulin measurements.

Blood samples were collected via cardiac puncture from euthanized mice or from the tail vein of live mice and were centrifuged at 1,500*g* for 10 minutes to obtain serum. Serum nonesterified fatty acid (NEFA) levels were measured by enzymatic assay using a commercially available NEFA measurement system (Wako Chemicals). Serum TG levels were quantified using the Triglyceride Quantification system (Thermo Fisher, catalog TR0100). Serum insulin levels were quantified using a Mouse Insulin ELISA kit according to the manufacturer’s instructions (Crystal Chem).

### Liver and serum cholesterol measurement.

Cholesterol levels in liver tissue and serum were measured using the Cholesterol Quantitation system (MilliporeSigma, catalog MAK043). To quantify cholesterol levels, 10 μL serum was diluted with 90 μL of the provided cholesterol assay buffer. The cholesterol levels were measured by adding the working reagent, which contains cholesterol esterase, cholesterol oxidase, and peroxidase, to each sample and incubating for 30 minutes at 37°C. The absorbance was measured at 570 nm, and cholesterol concentrations were calculated from a standard curve generated with known cholesterol standards. Liver tissue was homogenized in the provided cholesterol assay buffer. Briefly, 100 mg liver tissue was homogenized in 1 mL buffer, and the homogenate was centrifuged at 10,000*g* for 10 minutes at 4°C to remove debris. The supernatant was collected, and protein concentration was determined using the BCA protein assay. For cholesterol quantification, 20 μL liver supernatant was mixed with 180 μL cholesterol assay buffer, followed by the addition of the working reagent. The reaction was incubated at 37°C for 30 minutes, and absorbance was measured at 570 nm.

### Serum and liver lipidomics analysis.

Lipidomics was performed by the Harvard Chan Advanced Multi-Omics Platform. Lipids were extracted in butanol/methanol (1:1) with 5 mM ammonium formate. Extraction solvent (20 μL) was used per milligram tissue, or 10 μL per microliter serum. The mixture was vortexed vigorously and then centrifuged at 13,000*g* for 10 minutes at 4°C. The supernatant (30 μL) was transferred to an HPLC vial with a glass insert, and 5 μL of this sample was injected into the liquid chromatography-mass spectrometry (LC-MS) system for analysis. The instrument used was the Dionex Ultimate 3000 QExactive mass spectrometer (Thermo Fisher Scientific). Chromatographic separation was achieved on an Acquity UPLC CSH C18 column (130 Å, 1.7 μm, 2.1 mm × 100 mm) at 50°C. Mobile phase A was acetonitrile and water (6:4), and phase B was isopropanol and acetonitrile (9:1), with both phases containing 10 mM ammonium formate and 0.1% formic acid. The elution gradient was 0–3 minutes, 20% B; 3–7 minutes, 20%–55% B; 7–15 minutes, 55%–65% B; 15–21 minutes, 65%–70% B; 21–24 minutes, 70%–100% B; 24–26 minutes, 100% B; 26–28 minutes, 100%–20% B; and 28–30 minutes, 20% B, with a flow rate of 0.35 mL/min. The autosampler was at 4°C. The injection volume was 5 μL. MS analysis was performed in positive and negative ionization polarities using a combined full mass scan and data-dependent MS/MS (Top 10) (Full MS/dd-MS2) approach. The precursor ion scan had resolving power of 70,000, covering 100–1,200 *m/z*. For the product ion scan, the resolving power was 17,500, the isolation width was 1 *m/z*, and the stepped normalized collision energy was 10, 20, and 40 eV. The intensity threshold of the precursor ions for dd-MS2 analysis and the dynamic exclusion were set to 1.6 × 10^5^ and 10 seconds, respectively. Thermo Scientific LipidSearch software version 5.0 was used for lipid identification and quantitation. First, the product search mode was used to identify lipids according to the exact mass of the precursor ions and the MS2 mass spectra of the product ion scan. The precursor and product tolerance was 10 ppm. The absolute intensity threshold of the precursor ions and the relative intensity threshold of the product ions were set to 30,000 and 1%, respectively. Next, the search results from all samples were aligned within a retention time tolerance of 0.25 minutes.

### De novo lipogenesis flux by D_2_O labeling.

De novo lipogenesis (DNL) flux was quantified by deuterated water (D_2_O) labeling in mice fasted overnight (~16 hours), refed immediately after an i.p. D_2_O bolus (27 μL/kg), and maintained on 4% D_2_O drinking water for 6 hours prior to tissue harvesting under isoflurane anesthesia. Approximately 25 mg powdered liver was extracted with 0.6 mL 50% methanol containing 50 mM KOH (to remove free fatty acids) and 1.0 mL hexane (to extract TGs), homogenized with beads (CryoMill, 20 seconds, room temperature), centrifuged at 10,000*g* for 5 minutes, and 0.7 mL hexane supernatant was SpeedVac dried at 40°C for 30 minutes before saponification to free fatty acids with 0.3 M KOH in methanol (60°C, 90 minutes). The saponified solution was neutralized with 8 μL formic acid, diluted 20-fold with methanol, centrifuged at 17,000*g* for 10 minutes, and then the supernatant was transferred to LC-MS vials for analysis. Free fatty acids were resolved on a Vanquish UHPLC coupled to an Acquity CSH C18 column (1.7 μm, 2.1 × 100 mm) with a guard column (1.7 μm, 2.1 × 5 mm) using mobile phase A (water/acetonitrile at 95:5 with 10 mM ammonium acetate) and mobile phase B (methanol) under the following gradients: 20% B (0–0.3 minutes), 75% B (3 minutes), 95% B (9–14 minutes), and reequilibration at 20% B (14.1–15 minutes), with the autosampler at 4°C and a 2 μL injection volume. MS was performed on an Exploris 480 at 480,000 resolutions, acquiring palmitate in negative mode (scan 250–300 *m/z*, deprotonated anion) and cholesterol in positive mode (scan 300–400 *m/z*, protonated cation with neutral loss of water). Raw data were processed in El-MAVEN, and palmitate deuterium labeling was quantified as a weighted sum of isotopolog peak intensities, ∑(Mi × *i*)/22, where *i* is the number of incorporated deuterium atoms and 22 denotes the maximum number of exchangeable hydrogens in palmitate, with fractional enrichment reported as the mole percentage enrichment after natural abundance correction within the software workflow. Hepatic DNL was interpreted from fractional deuterium enrichment of palmitate within hepatic TGs.

### In vivo VLDL secretion.

Male were fasted for 16 hours (3 pm–7 am). A baseline (0 h) blood sample was then collected from the tail vein followed by an i.p. injection of 10% tyloxapol/saline solution (MilliporeSigma) (500 mg/kg body weight). Blood was then collected from the tail vein 1, 2, 3, and 4 hours after the injection, and plasma was separated for measurement of TG levels.

### Histology.

Livers were fixed in 10% zinc formalin overnight and then transferred to 70% ethanol for further preservation. Tissue processing, sectioning, and staining with H&E, CD68 and α-SMA were performed by HistoWiz, a fee-for-service histology service provider. The stained tissue sections were used for assessment. Histopathological evaluations were conducted by 2 independent pathologists blinded to the sample IDs, included quantification of lobular (foci) inflammation.

### Statistics.

Statistical significance was assessed using GraphPad Prism 10 (GraphPad Software). For comparisons between 2 groups, significance was determined using a 2-tailed, unpaired *t* test. Multiple group comparisons were analyzed by 1-way ANOVA. For comparisons between groups over multiple time points, 2-way ANOVAs were performed. A *P* value threshold of less than 0.05 was considered statistically significant. Unless otherwise stated, all data are presented as the mean ± SEM.

### Study approval.

All animal experiments were reviewed and approved by the Harvard Medical Area Standing Committee on Animals (HMA IACUC). All studies were conducted in accordance with institutional guidelines and applicable regulations.

### Data and materials availability.

Key resources including antibodies and commercial kits used in this study are listed in Supplemental Table 3. Values for all data points shown in graphs and all values underlying reported means for the main manuscript and the Supplemental figures are provided in the Supporting Data Values file, as are the microscopy data reported in this work. All materials used in the analysis are available to any researcher for purposes of reproducing the analysis.

## Author contributions

SD designed the study, performed experiments, analyzed data, interpreted results, and wrote the manuscript. GYL, GA, and OC performed mutagenesis, co-IP experiments, and protein purification. JEF and KEI performed the in vivo experiments, including VLDL secretion assays. ZC performed the microscopy. BY and STH performed the lipidomics and de novo lipogenesis assays. IG performed the MCD-induced MASH phenotype characterization. GSH designed and supervised the study, interpreted results, generated project resources, and wrote the manuscript together with SD. All authors reviewed and commented on the manuscript.

## Conflict of interest

GSH is a member of the Scientific Advisory Board and holds equity in and receives compensation from Crescenta Pharmaceuticals. GSH’s laboratory receives funding from Enlila through a sponsored research agreement.

## Funding support

This work is the result of NIH funding, in whole or in part, and is subject to the NIH Public Access Policy. Through acceptance of this federal funding, the NIH has been given a right to make the work publicly available in PubMed Central.

- National Heart, Lung, and Blood Institute (NHLBI) (R01 HL148137, to GSH).
- Hotamisligil Laboratory Funds (to GSH).
- Instituto de Salud Carlos III (PI22/00776 and BA20/00016, to IG).
- SD receives salary support from the Hotamisligil laboratory.

## Supplementary Material

Supplemental data

Unedited blot and gel images

Supporting data values

## Version 1 07/15/2026

Electronic publication
