# Supplementary material for: Cholesterol-responsive NFE2L1-INSIG1 interaction controls VLDL secretion and metabolic dysfunction–associated steatohepatitis pathogenesis in mice
Source: J Clin Invest. 2026 Jul 15;136(14):e197094. doi: 10.1172/JCI197094 (PMC13367976; doi:10.1172/JCI197094)
Supplement: Supplemental data [file jci-136-197094-s328.pdf]

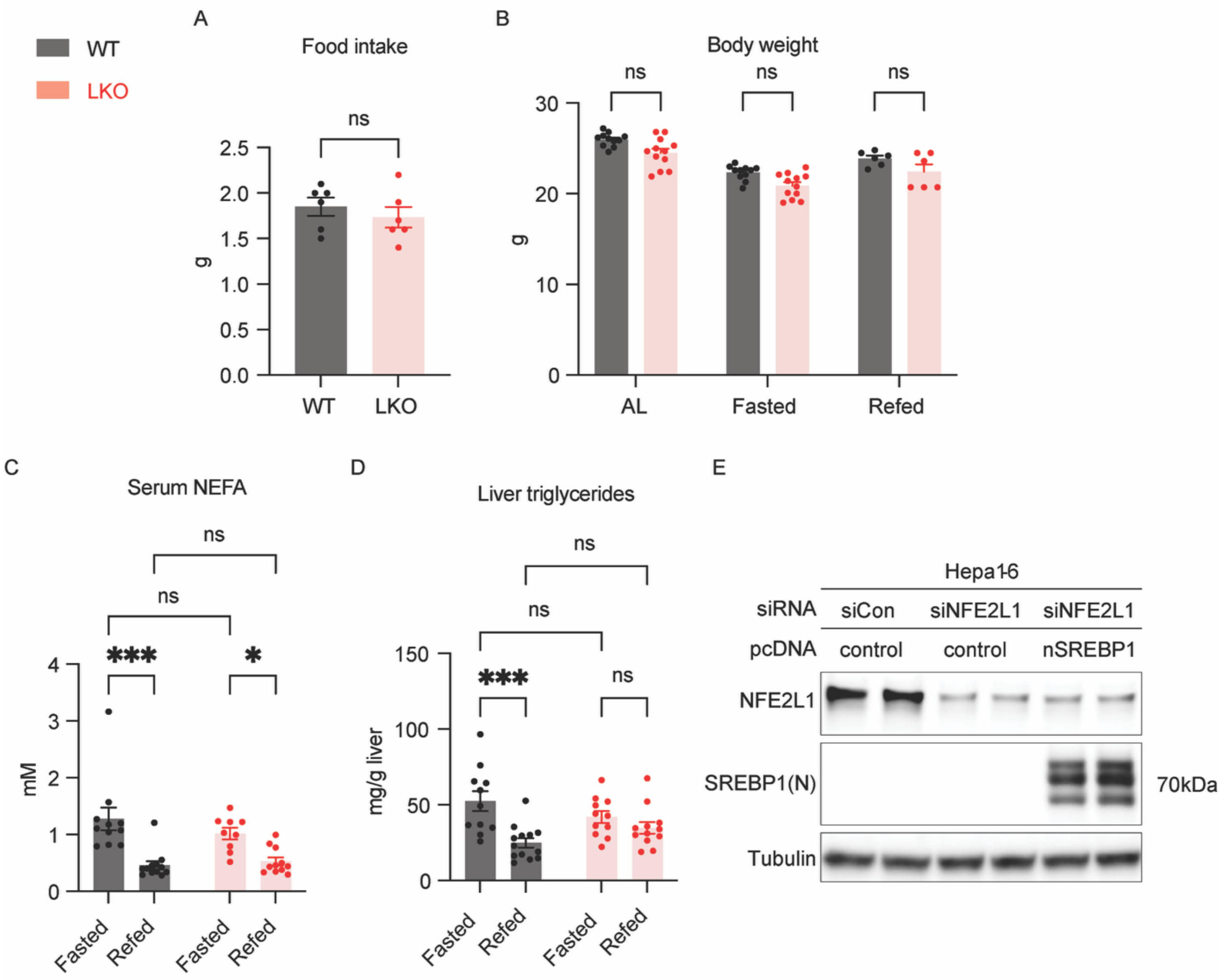

Figure S1. NFE2L1 deficiency does not alter food intake, body weight, or serum NEFA. (A) Food intake during a 6-hour refeeding period after 16-hour fasting in NFE2L1 WT and LKO mice (n=6/group). (B) Body weights of WT and LKO mice under ad libitum (AL), 16-hour fasted (Fasted), and 6-hour refed (Refed) conditions. (C) Serum NEFA levels measured by ELISA (n=11/group). (D) Liver TG levels. (E) Immunoblot analysis and quantification of SREBP1 in Hepa1-6 hepatocytes after transient siRNA-mediated NFE2L1 knockdown (24 hours) and re-expression of SREBP1 (N). Data are mean ± SEM. Statistical significance assessed by two-way ANOVA (C, D) or Student's t-test (A, B) (*P < 0.05, ***P < 0.001).

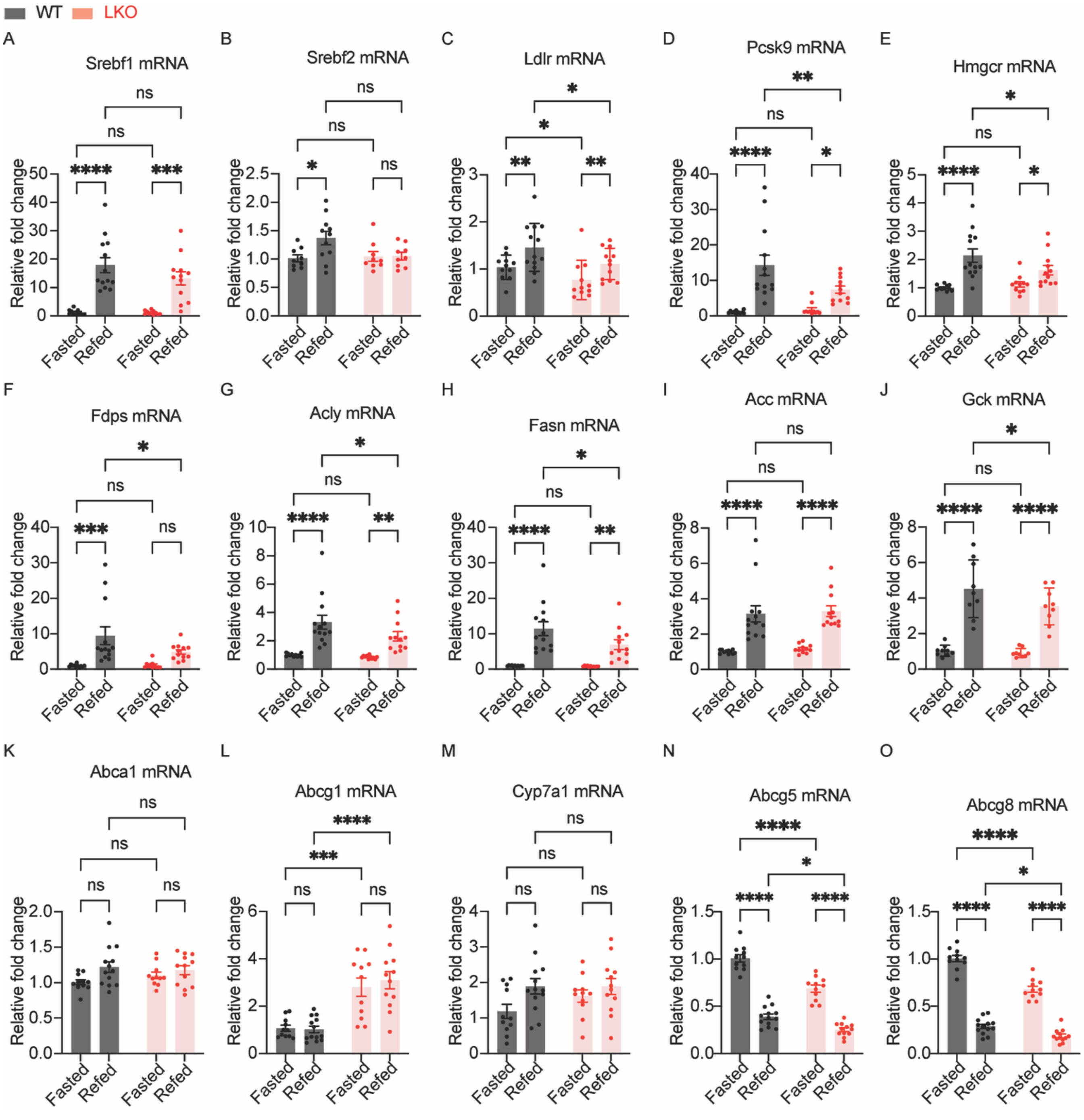

Figure S2. Hepatic mRNA levels of SREBP and LXR target genes in WT and LKO mice.

8-week-old NFE2L1 WT or LKO mice were fasted for 16 hours or fasted for 16 hours followed by 6-hour refeeding (n=11/group). Gene expression was normalized to 18S ribosomal RNA. Gray and red bars denote WT and LKO mice, respectively. Statistical significance was assessed by two-way ANOVA (*P < 0.05, **P < 0.0, ***P < 0.001, ****P < 0.0001).

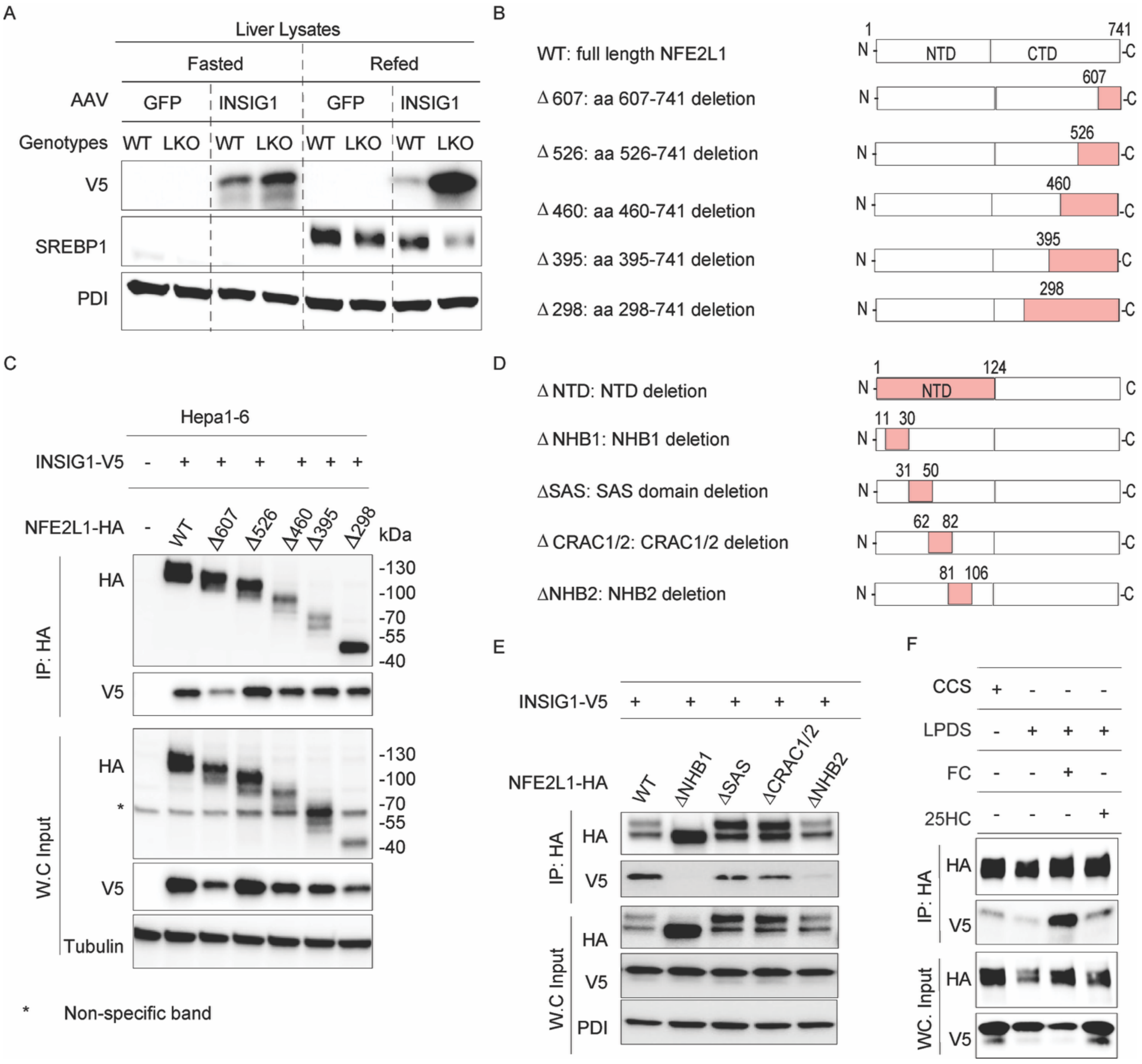

Figure S3. The N terminal domain (NTD) of NFE2L1 binds to INSIG1 in the ER.

(A) Western blot analysis of INSIG1-V5 in liver from WT and LKO mice injected with AAV-GFP or INSIG1-V5 under fasting or refed conditions. (B) Schematic representation of NFE2L1 truncation mutants lacking regions in the C-terminal domain (CTD). Red color indicates deleted regions. (C) Co-immunoprecipitation (Co-IP) of INSIG1-V5 with HA-tagged NFE2L1 CTD mutants in Hepa1-6 cells. Lysates were harvested 48 h post-transfection and immunoprecipitated with HA beads. (D) Schematic representation of NFE2L1 truncation mutants lacking regions in the N-terminal domain (NTD). (E) Co-IP of INSIG1-V5 with HA-

tagged NFE2L1 NTD mutants, performed as described in panel (B). (F) Sterol-dependent interaction between NFE2L1 and INSIG1. Cells transfected with NFE2L1-WT and INSIG1-V5 were treated for 3 h with 5% cosmic calf serum (CCS), 5% lipoprotein-deficient serum (LPDS), 50 μM free cholesterol (FC), or 1 μM 25-hydroxycholesterol (25HC). Lysates were subjected to Co-IP with HA beads. Western blots are representative of ≥3 independent experiments.

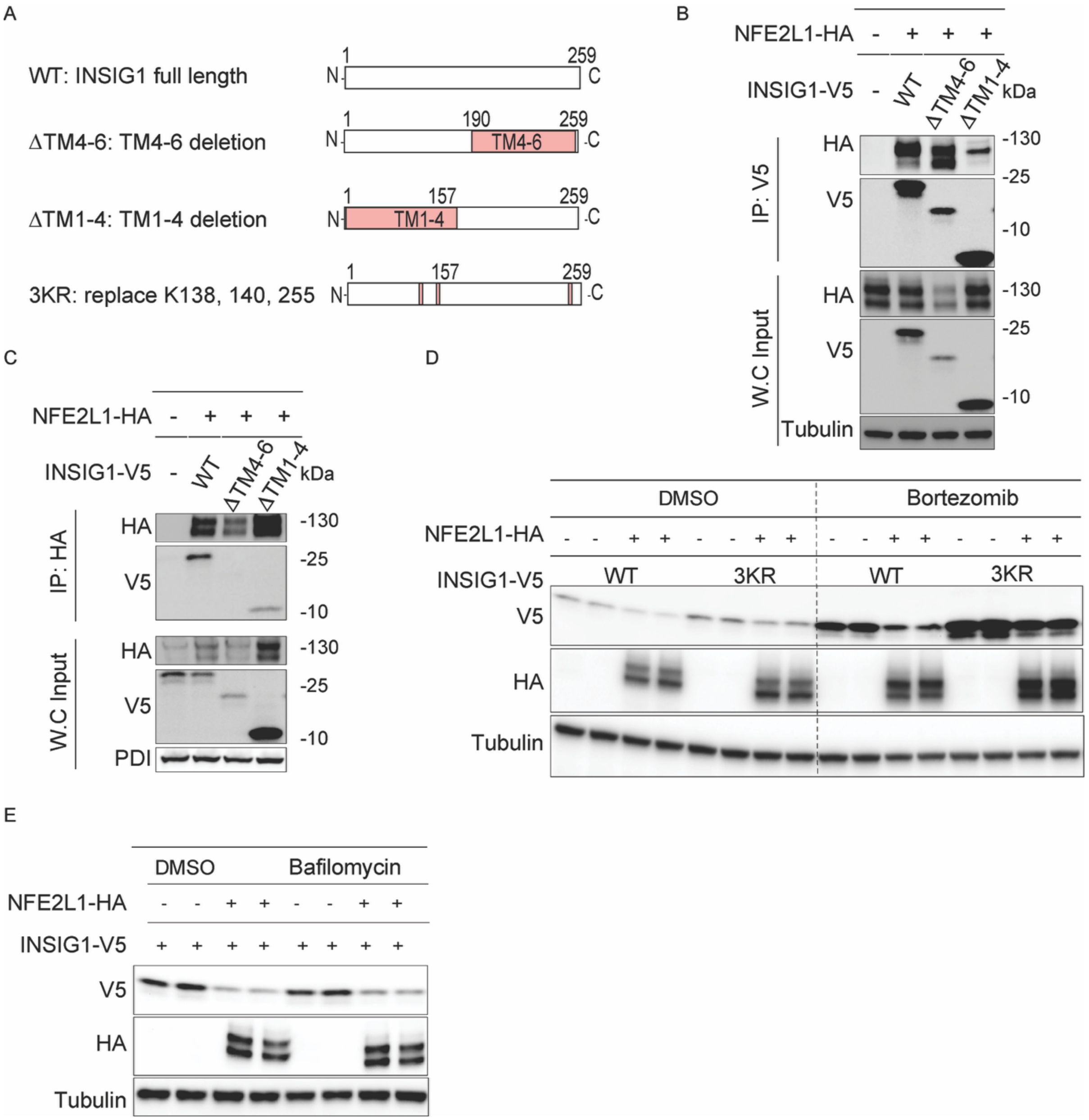

Figure S4. Mechanisms of INSIG1 degradation.

(A) Schematic representation of INSIG1 truncation mutants lacking regions in the transmembrane domain (TM). Red indicates deleted regions. (B) Co-immunoprecipitation (Co-IP) of INSIG1-V5 mutants with HA-tagged NFE2L1 in Hepa1-6 cells. Lysates were harvested 48 h post-transfection and immunoprecipitated with HA beads. (C) Co-IP of NFE2L1 with V5-tagged INSIG1 mutants, performed as in (B). (D) Immunoblotting of INSIG1 WT and mutant 3KR after proteasome inhibition. Cells transfected with NFE2L1-WT and INSIG1-V5 were treated for 3 h with proteasome inhibitor Bortezomib. (E) Immunoblotting of INSIG1 WT and

NFE2L1 after lysosome inhibition. Cells transfected with NFE2L1-WT and INSIG1-V5 were treated for 3 h with lysosome inhibitor bafilomycin. Immunoblots are representative of ≥3 independent experiments.

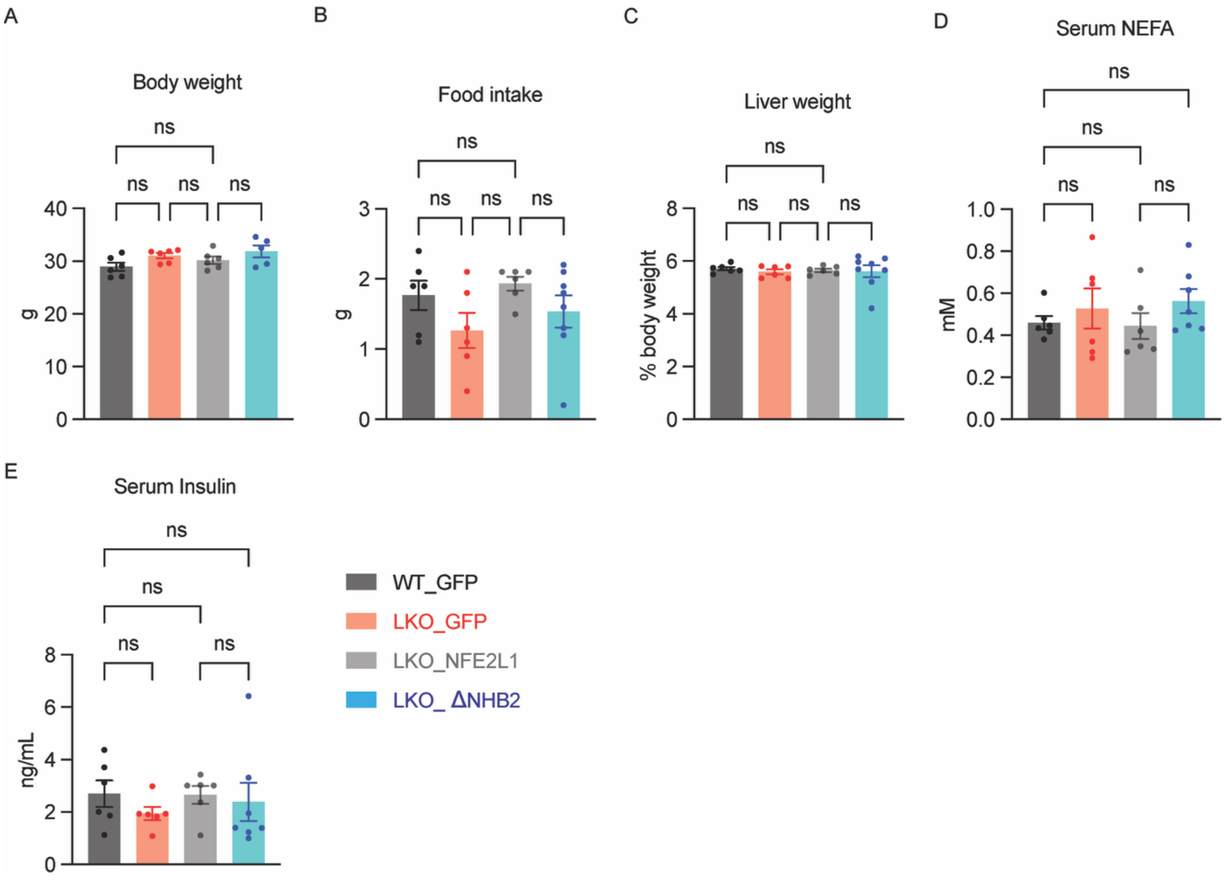

Figure S5. AAV-mediated NFE2L1 expression does not alter metabolic parameters in mice.

NFE2L1 wild-type (WT) or liver-specific knockout (LKO) mice (8-week-old) were injected with GFP, NFE2L1-WT, or NFE2L1-ΔNHB2 containing AAV-based expression constructs. Three weeks post-injection, mice were fasted overnight and refed for 6 hours (n=6/group). (A) Body weight. (B) Food intake during refeeding. (C) Liver weights. (D) Serum non-esterified fatty acid (NEFA) levels. (E) Serum insulin levels. Data are mean ± SEM. Statistical significance was assessed by one-way ANOVA.

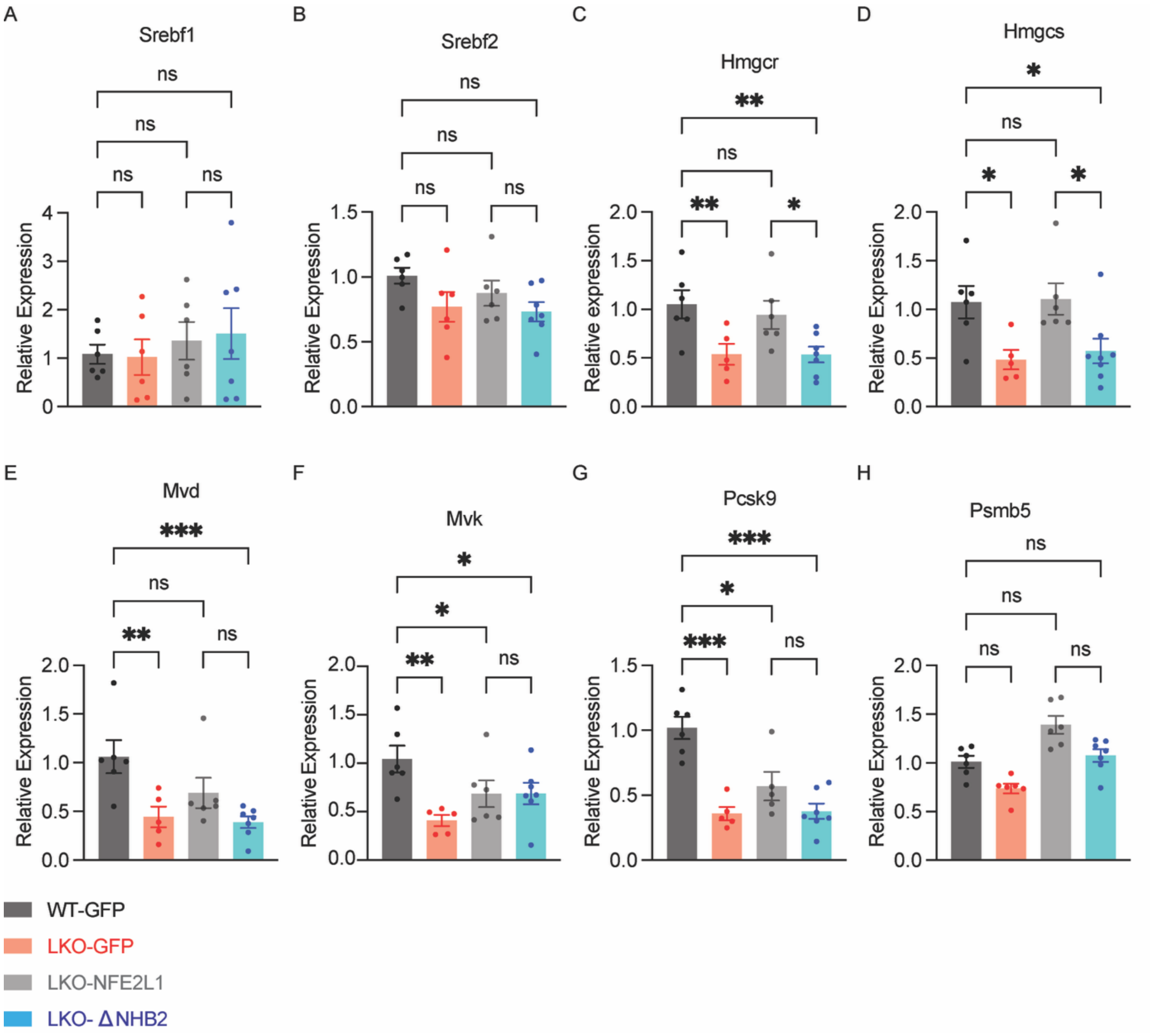

Figure S6. AAV-mediated restoration of NFE2L1 rescues SREBP target gene expression in livers of NFE2L1-LKO mice. NFE2L1 wild-type (WT) or liver-specific knockout (LKO) mice (8-week-old) were injected with GFP, NFE2L1-WT, or NFE2L1-ΔNHB2 containing AAV-based expression constructs. Three weeks after injection, mice were fasted overnight then refed for 6 hours (n=6/group). (A-H) Relative mRNA levels of SREBP target genes in refed livers. Expression of the genes were normalized to 18S. Data are the means ± SEM. Statistical significance was assessed by one-way ANOVA.

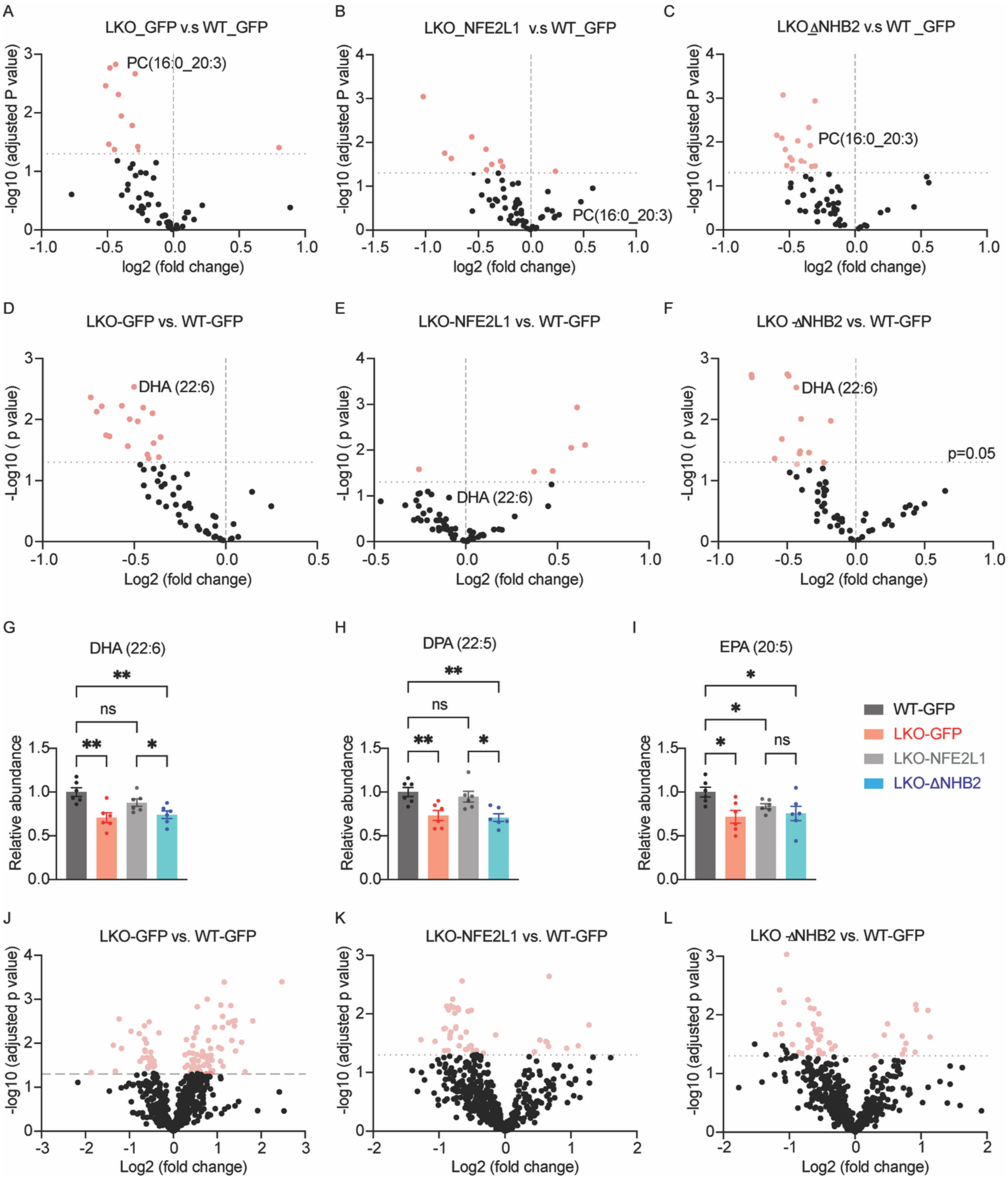

Figure S7. Effect of NFE2L1 -INSIG1 axis on serum phosphatidylcholine (PC) and PUFAs.

(A-C) Serum lipidomic analysis for phospholipid species with different fatty acid compositions (n=6 per group). (D-F) Fatty acid composition analysis within serum TG pool (n=6/group). (G-I)

Serum DHA, DPA and EPA within TG pool. (J-L) Liver lipidomic analysis for TG species with different fatty acid compositions (n=6 per group). Red indicates $p<0.05$. Statistical significance was assessed by one-way ANOVA (G, H, I), *$p<0.05$.

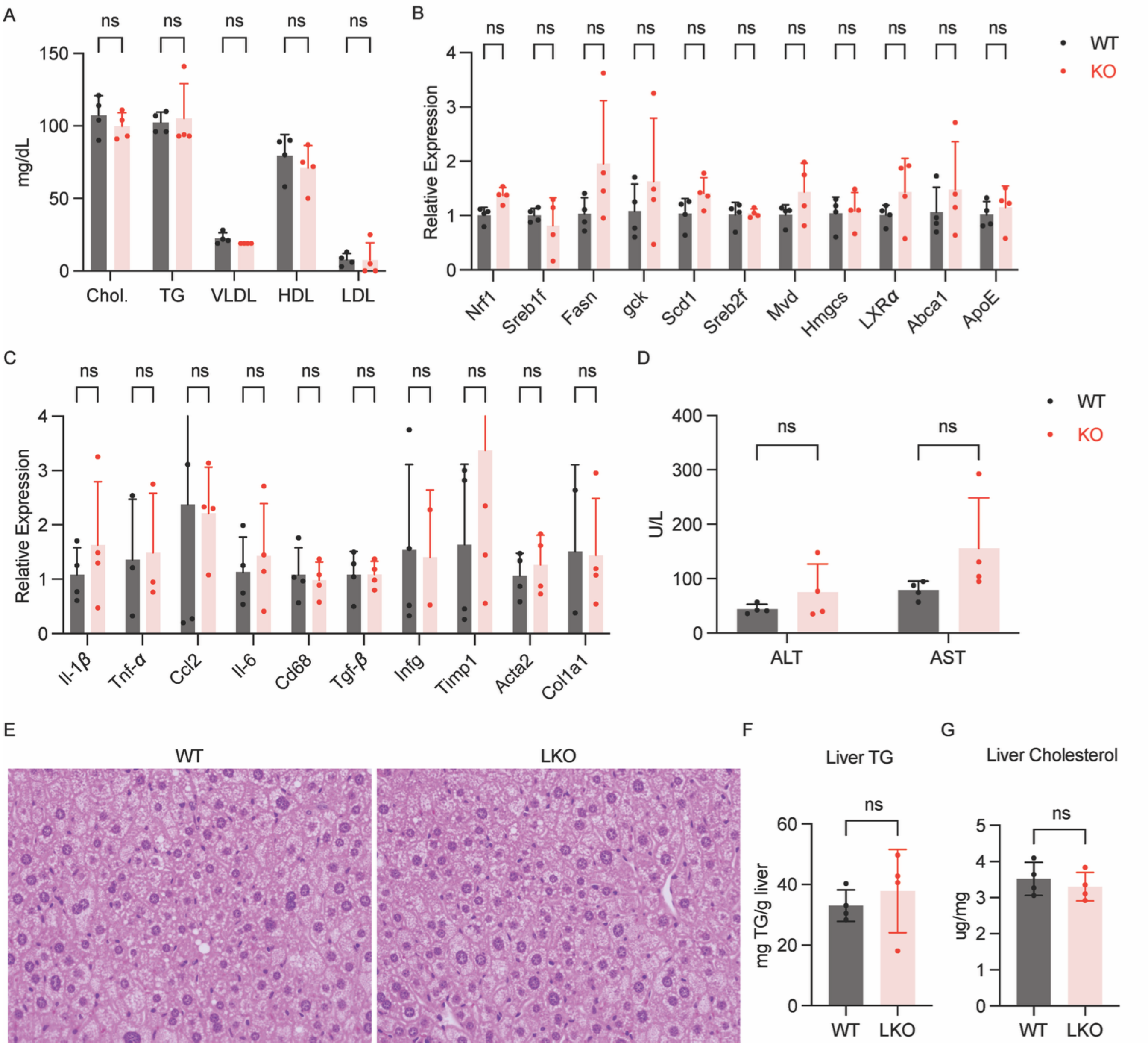

Figure S8. Role of NFE2L1 in high fat diet induced fatty liver.

Liver-specific knockout mice and their littermates were fed with fed with high fat diet for 2 months. Mice were fed ad libitum before samples collection (n=4-5/group). (A) Serum lipids profiles measured via Piccolo Lipid Panel Plus. (B) Liver mRNA levels of SREBP1/2 and LXR target genes normalized to 18S ribosomal RNA (18S). (C) mRNA levels of liver inflammation and fibrosis markers. (D) Serum ALT and AST levels (Piccolo Lipid Panel Plus). (E) Representative histology images of liver section staining H&E. (F) Quantification of liver TG and cholesterol. Data: Mean ± SEM. Statistics: t-test (*P < 0.05).

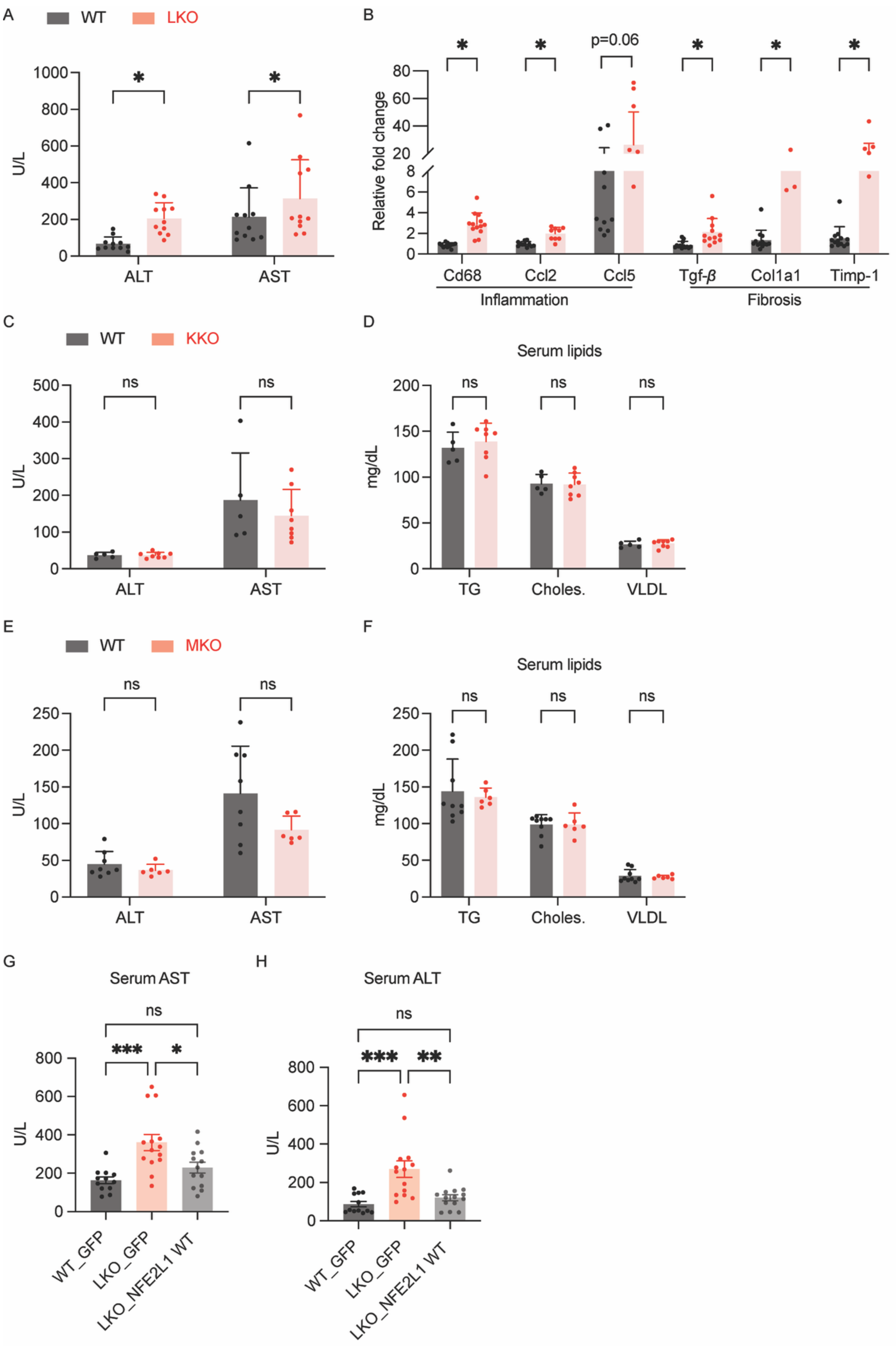

A
WT
LKO
U/L
ALT
AST
B
p=0.06
Relative fold change
Cd68
Ccl2
Ccl5
Tgf-β
Col1a1
Timp-1
Inflammation
Fibrosis
C
WT
KKO
ns
D
Serum lipids
mg/dL
TG
Choles.
VLDL
E
WT
MKO
F
G
Serum AST
H
Serum ALT
WT_GFP
LKO_GFP
LKO_NFE2L1 WT

Figure S9. NFE2L1 deficiency exacerbates liver injury, inflammation, and fibrosis.

NFE2L1-WT or -LKO mice (8-week-old) mice were either fasted for 16 hours or fasted for 16 hours then refed for 6 hours (n=11/group). (A) Serum ALT and AST. (B) Relative mRNA levels of genes involved in inflammation and fibrogenesis. Total RNA was extracted from livers and subjected to quantitative real-time PCR analysis. Expression of the genes was normalized to 18S. (C-D) 8-week-old NFE2L1-WT or Kupffer cell-specific deletion (Nfe2l1-Clec4f-Cre, KKO) mice were fasted overnight (n=6-8/group). Serum ALT and AST (C) and lipid profiles (D) were measured. (E-F) 8-week-old NFE2L1-WT or myeloid-specific NFE2L1 deletion (Nfe2l1-LysM-Cre, MKO) mice were fasted overnight (n=6-8/group). Serum ALT and AST (E) and lipid profiles (F) were measured. (G-H) 8-week-old NFE2L1-WT or -LKO mice were injected with AAV-GFP, AAV-NFE2L1-WT. Three weeks after injection, mice were fasted overnight then refed for 6 hours (n=6/group). Serum ALT (G) and AST (H) were measured. Data are the means ± SEM. Statistical significance was assessed by t-test (*$P < 0.05$).

A

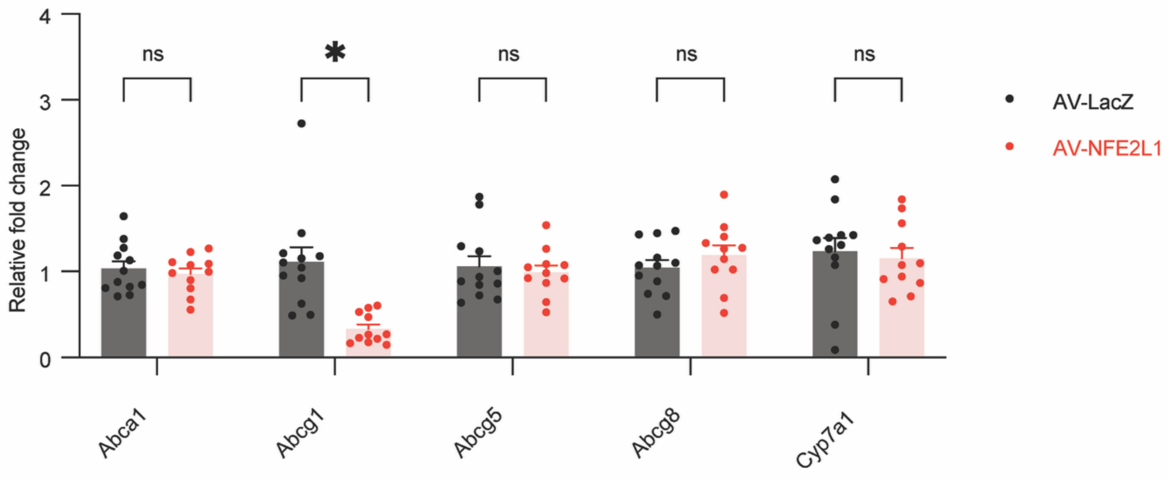

Figure S10. The role of NFE2L1 in LXR activity.

8-week-old db/db mice were injected with either AV-LacZ or AV-NFE2L1 and fed with MCD diet for 2 weeks. Mice were fed ad libitum before sample collection (n=12/group). (A) Liver mRNA levels of LXR target genes normalized to 18S ribosomal RNA (18S). Data: Mean ± SEM. Statistics: t-test (*P < 0.05).

TABLES

Table S1. Lipidomic Profiling of Serum Triglycerides Species

Compared to WT-GFP mice					
LKO-GFP		LKO-NFE2L1		LKO-NHB2	
**TG species**	**-Log10**	**TG species**	**-Log10**	**TG species**	**-Log10**
TG(20:0_18:1_22:6)	3.804	TG(O-18:0_16:0_18:1)	3.089	TG(20:0_18:1_22:6)	4.452
TG(18:1_18:2_22:6)	3.766	TG(16:0_16:0_18:0)	2.480	TG(19:1_18:2_22:6)	3.790
TG(16:0_18:2_20:5)	3.584	TG(16:0_18:2_18:3)	2.316	TG(18:1_20:1_22:6)	3.759
TG(19:1_18:2_22:6)	3.536	TG(O-20:0_16:0_18:1)	2.110	TG(14:0_16:0_18:0)	3.523
TG(18:1_19:1_22:6)	3.460	TG(O-24:2_16:0_18:1)	2.055	TG(18:1_20:4_24:5)	3.449
d5-TG(40:9_18:0)	3.177	TG(10:0_14:0_16:0)	2.041	TG(16:0_16:0_18:0)	3.275
TG(18:1_20:1_22:6)	3.013	TG(14:0_16:0_18:0)	1.929	TG(15:0_18:1_22:6)	3.166
TG(16:1_18:1_22:1)	2.986	TG(16:0_18:2_20:5)	1.912	TG(17:0_18:1_22:6)	3.026
TG(14:0_15:0_22:6)	2.890	TG(15:0_18:1_22:6)	1.872	TG(16:1_18:3_18:3)	2.897
TG(17:0_18:1_22:6)	2.817	TG(16:0_18:2_22:6)	1.769	d5-TG(40:9_18:0)	2.668
TG(19:0_18:1_18:2)	2.803	TG(O-24:2_16:0_18:0)	1.718	TG(16:0_22:0_22:6)	2.448
TG(16:0_22:0_22:6)	2.750	TG(O-16:0_16:0_18:1)	1.679	TG(17:0_18:1_24:6)	2.431
TG(18:1_19:1_18:2)	2.739	TG(28:1_9:0)+OO:(s)	1.560	TG(16:0_20:0_22:6)	2.385
TG(18:1_22:1_18:2)	2.570	TG(O-20:1_16:0_18:1)	1.545	TG(16:0_24:5_22:6)	2.382
TG(22:0_18:1_18:2)	2.564	TG(15:0_18:2_22:6)	1.534	TG(15:0_16:1_22:6)	2.361
TG(21:0_18:1_18:2)	2.547	TG(O-16:0_16:0_18:2)	1.495	TG(18:1_20:3_22:6)	2.349
TG(17:1_18:2_22:6)	2.527	TG(18:2_22:6_22:6)	1.481	TG(16:0_18:2_22:6)	2.277
TG(16:0_24:1_22:6)	2.518	TG(18:2_22:6_24:6)	1.344	TG(14:0_15:0_22:6)	2.269
TG(18:1_22:1_22:6)	2.514	TG(18:2_18:2_22:6)	1.338	TG(14:0_18:1_18:3)	2.219
TG(15:0_16:1_22:6)	2.448	TG(16:0_17:0_18:0)	1.335	TG(17:1_18:1_22:6)	2.213
TG(18:1_18:1_18:2)	2.439	TG(16:1_18:3_18:3)	1.319	TG(14:0_18:1_18:2)	2.156

TG(15:0_18:1_22:6)	2.420	TG(14:0_18:1_18:3)	1.302	TG(16:1_18:1_22:1)	2.110
TG(17:0_18:1_24:6)	2.397			TG(19:0_18:1_18:2)	2.099
TG(16:0_18:1_23:1)	2.364			TG(16:0_24:1_22:6)	2.079
TG(17:1_18:1_22:6)	2.313			TG(16:0_18:1_22:5)	2.071
TG(16:0_21:0_22:6)	2.312			TG(16:0_21:0_22:6)	2.069
d5-TG(36:4_18:2)	2.281			TG(16:0_18:1_22:6)	2.050
TG(15:0_18:2_22:6)	2.229			TG(16:0_18:2_20:5)	2.015
TG(16:0_24:5_22:6)	2.174			TG(15:0_18:2_22:6)	2.005
TG(16:0_24:0_22:6)	2.170			TG(18:0_18:1_22:6)	1.972
TG(16:0_20:5_22:6)	2.166			TG(12:0_18:1_18:3)	1.922
TG(16:0_18:1_22:6)	2.165			TG(18:1_22:6_24:6)	1.908
TG(16:0_18:2_22:6)	2.157			TG(16:0_18:2_18:3)	1.898
TG(16:1_22:6_22:6)	2.151			TG(17:1_18:2_22:6)	1.862
TG(18:1_20:1_20:3)	2.034			TG(16:0_20:5_22:6)	1.853
TG(18:1_22:6_22:6)	1.896			TG(18:2_22:6_24:6)	1.825
TG(17:1_18:1_18:2)	1.868			TG(18:1_22:5_22:6)	1.810
TG(22:1_18:2_22:6)	1.849			TG(18:1_22:6_22:6)	1.750
TG(18:0_18:1_22:6)	1.849			TG(18:2_22:5_22:6)	1.735
TG(18:0_18:1_18:2)	1.809			TG(16:0_24:0_22:6)	1.627
TG(16:0_18:1_24:1)	1.806			TG(17:0_18:1_22:5)	1.616
TG(18:2_18:2_22:6)	1.804			TG(18:1_24:5_22:6)	1.601
TG(18:2_18:2_20:5)	1.797			TG(17:0_18:1_20:4)	1.559
TG(16:1_18:3_18:3)	1.754			TG(18:1_18:2_22:6)	1.543
TG(14:0_16:0_18:0)	1.750			TG(18:1_20:1_20:3)	1.541
TG(16:0_18:1_22:5)	1.742			TG(14:0_18:2_18:3)	1.491
TG(16:0_18:2_18:2)	1.735			TG(14:0_16:0_18:3)	1.473

TG(O-15:2_3:0_18:2)	1.732			TG(27:1_9:0)+OO:(s)	1.460
TG(16:0_16:0_18:0)	1.732			TG(21:0_18:1_18:3)	1.431
TG(22:7_16:0_20:5)	1.714			TG(18:1_22:1_18:2)	1.413
TG(O-18:0_18:1_18:2)	1.701			TG(18:1_20:4_22:6)	1.396
TG(18:2_22:5_22:6)	1.689			TG(28:0_20:5)	1.362
TG(15:0_18:1_18:2)	1.685			d5-TG(36:6_20:2)	1.362
TG(17:0_18:1_20:4)	1.671			TG(10:0_14:0_18:1)	1.352
TG(17:0_18:1_18:2)	1.654			TG(15:0_16:0_18:0)	1.342
TG(16:0_20:0_22:6)	1.647			TG(16:0_18:1_18:2)	1.335
TG(18:1_20:4_22:6)	1.611			TG(15:0_18:1_18:2)	1.310
TG(18:1_16:0_18:3)	1.513				
TG(14:0_18:1_18:2)	1.491				
TG(16:0_18:1_22:1)	1.468				
TG(12:0_18:1_18:3)	1.468				
TG(12:0_18:1_22:6)	1.448				
TG(18:1_20:3_22:6)	1.436				
TG(18:1_20:1_18:2)	1.422				
TG(17:0_18:1_20:1)	1.414				
TG(14:0_18:2_22:6)	1.413				
TG(14:0_18:1_18:3)	1.412				
TG(18:2_18:3_20:5)	1.395				
TG(16:0_18:2_18:3)	1.363				
TG(O-15:2_3:0_22:6)	1.361				
TG(19:0_18:1_20:4)	1.361				

Note: The table includes only TG species meeting statistical significance threshold [(-Log10(adjusted p value)>1.3, equivalent to $p<0.05$]

Table S2. Lipidomic Profiling of FA species within Serum Triglycerides Pool

Compared to WT-GFP mice					
LKO-GFP		LKO-NFE2L1		LKO-NHB2	
FA species	-Log10	FA species	-Log10	FA species	-Log10
(22:6)	2.537	(O-18:0)	2.930	(22:5)	2.744
(23:1)	2.364	(O-20:0)	2.110	(24:5)	2.735
(18:2)	2.226	(O-24:2)	2.051	(20:3)	2.714
(22:1)	2.218	(20:5)	1.580	(24:6)	2.692
(22:5)	2.196	(O-20:1)	1.545	(22:6)	2.527
(22:0)	2.129	(O-16:0)	1.531	(18:3)	2.012
(20:3)	2.104			(9:0+OO:(s))	1.978
(24:5)	2.007			(22:0)	1.680
(20:5)	1.971			(20:5)	1.481
(24:1)	1.743			(27:1)	1.460
(3:0)	1.726			(18:2)	1.444
(O-15:2)	1.726			(28:0)	1.362
(22:7)	1.714			(18:1)	1.295
(19:0)	1.614				
(24:6)	1.565				
(21:0)	1.429				
(18:1)	1.384				
(19:1)	1.360				

Note: The table includes only FA species meeting statistical significance threshold [(-Log10(adjusted p value)>1.3, equivalent to p<0.05]

Table S3. Key resources

Antibodies	Source	Identifier
Anti-Histone H3	Cell Signaling Technology	Cat# 9715
Anti-SCAP	Abcam	Cat# ab153933
Anti-NFE2L1	Cell Signaling Technology	Cat# 8052
Anti-PDI	Cell Signaling Technology	Cat# 3501
Anti-V5	Thermo scientific	Cat# R961-25
Anti-HA	Cell Signaling Technology	Cat# 3724
Anti-AKT	Cell Signaling Technology	Cat# 4691
Anti-pAKT	Cell Signaling Technology	Cat# 9271
Anti-INSIG1	Abcam	Cat# ab70784
Anti-SREBP1	Abcam	Cat# ab28481
Anti-CD68	Proteintech	Cat# 25747-1-AP
Anti-LDLR	Abcam	Cat# ab52818
Anti-beta Tubulin HRP	Cell Signaling Technology	Cat# ab21058
Chemicals and Reagents		
Bortezomib (PS-341)	Selleckchem	Cat# S1013
Epoxomycin	Millipore	Cat# 324800
Methyl-b-cyclodextrin	Sigma Aldrich	Cat# C4555
TRIzol	ThermoFisher Scientific	Cat# 15596018
Cholesterol	Sigma Aldrich	Cat# C8667
DMEM	GIBCO	Cat# 11965
Cosmic Calf Serum	Hyclone	Cat# SH30087.03
Sandoz 58-035	Sigma-Aldrich	Cat# S9318-5MG
Fos-Choline-13	Anagrade	Cat# 85775-42-4
Methyl-b-cyclodextrin	Sigma Aldrich	Cat# C4555

Adenovirus expressing b-galactosidase	Vector Biolabs	Cat# 1080
Adenovirus expressing wild type mouse Nfe2l1	Vector Biolabs	Cat# 1080
AAV-GFP	Penn Vector Core	V7400S
AAV-NFE2L1-HA-WT	Penn Vector Core	V7401S
AAV-dNHB2-HA	Penn Vector Core	V7397S
Critical Commercial Assays		
Piccolo Lipid Panel	Abaxis	Cat# 07-P02-12A
Cholesterol Assay Kits	Cell Biolabs Inc.	Cat# STA-384
NE-PER™ Nuclear Extraction Reagents	Thermo Scientific	Cat# 78833
Cell Lines and Mice		
Hepa1-6 cells	ATCC	CRL-1830
db/db mice	The Jackson Laboratory	Jax: 000664
Nfe2l1-flox mouse	International Mouse Phenotyping Consortium (IMPC)	Generated previously from Ref (13)
Albumin-Cre mice	The Jackson Laboratory	Jax: 003574
Nfe2l1-Clec4f-Cre mouse	The Jackson Laboratory	Jax: 004781
Nfe2l1-LysM-Cre mouse	The Jackson Laboratory	Jax: 033296
Regular chow diet	Picolab Rodent diet 20	
High fat diet	Research Diets	Cat#D12492
MCD diet	Research Diets	Cat#A02082002BR
