# Supplementary material for: Cholesterol-responsive NFE2L1-INSIG1 interaction controls VLDL secretion and metabolic dysfunction–associated steatohepatitis pathogenesis in mice
Source: J Clin Invest. 2026 Jul 15;136(14):e197094. doi: 10.1172/JCI197094 (PMC13367976; doi:10.1172/JCI197094)

Full unedited blot for Figure 1A

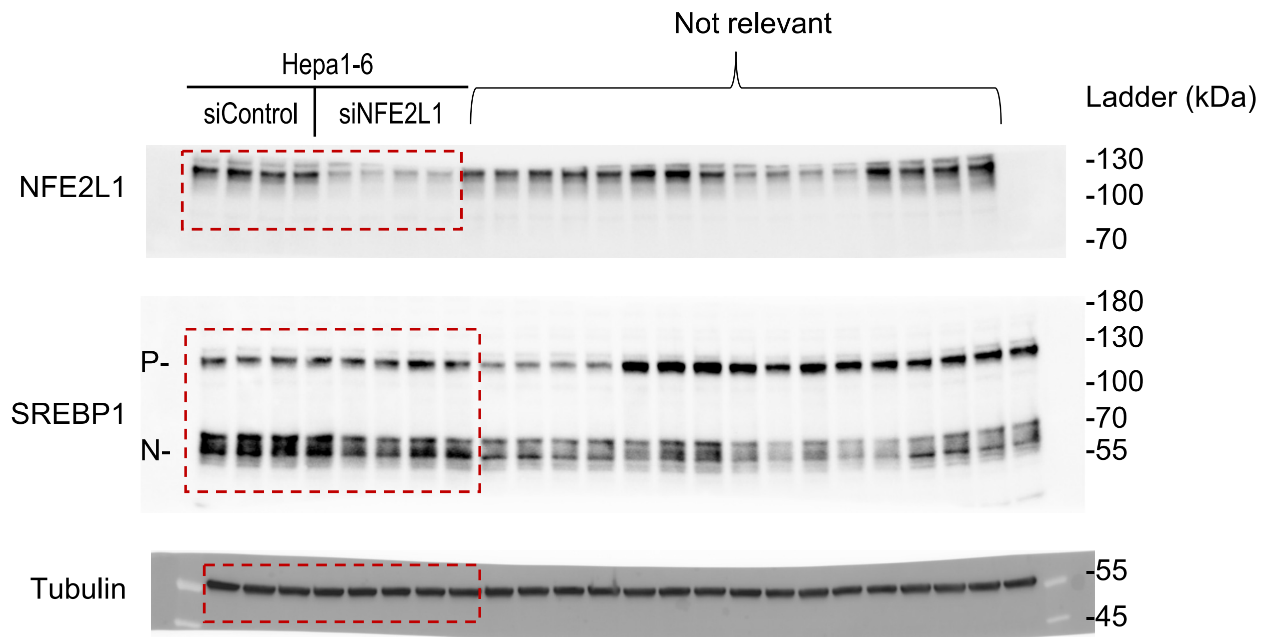

Full unedited blot for Figure 1C

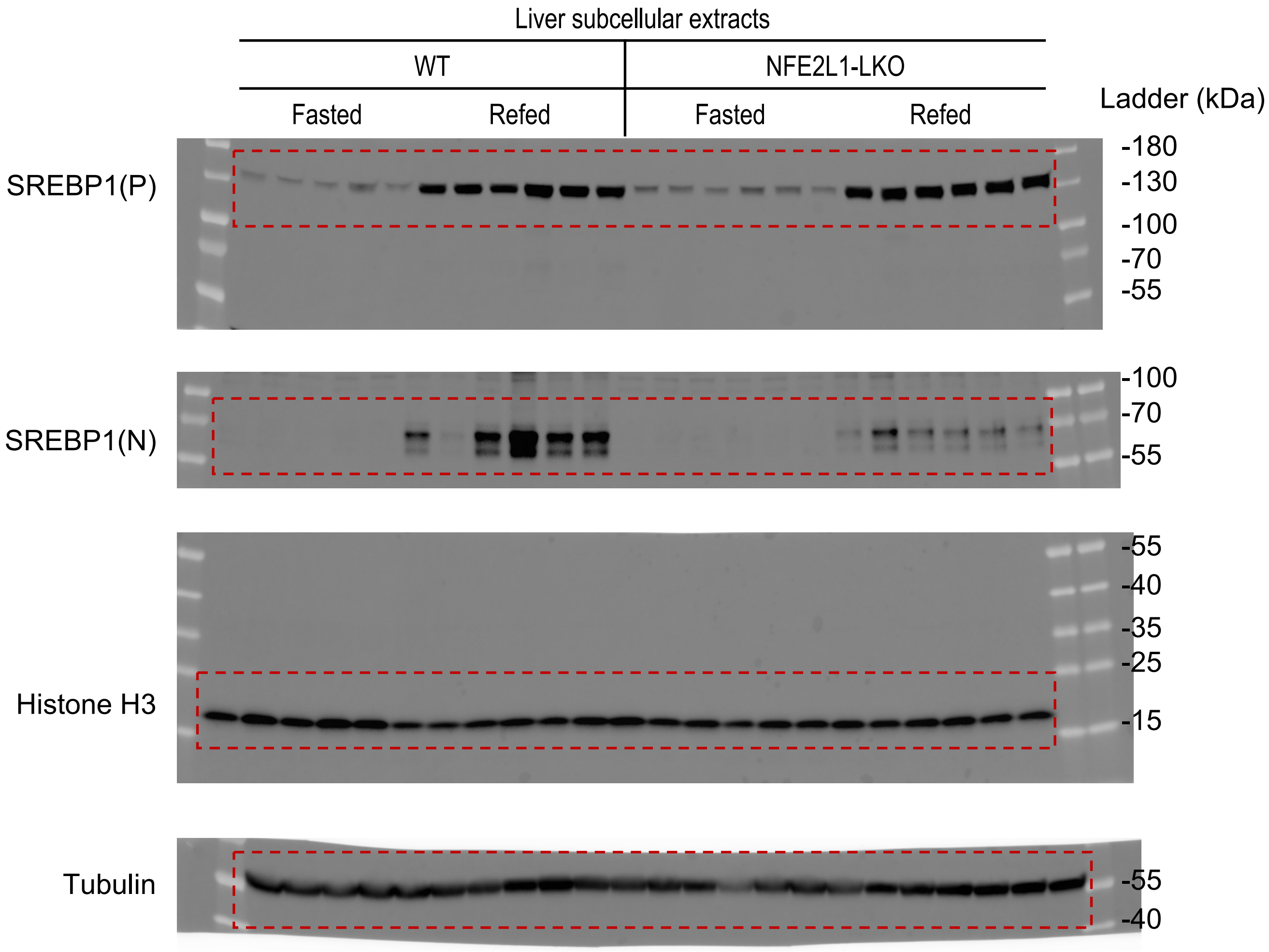

Full unedited blot for Figure 2A

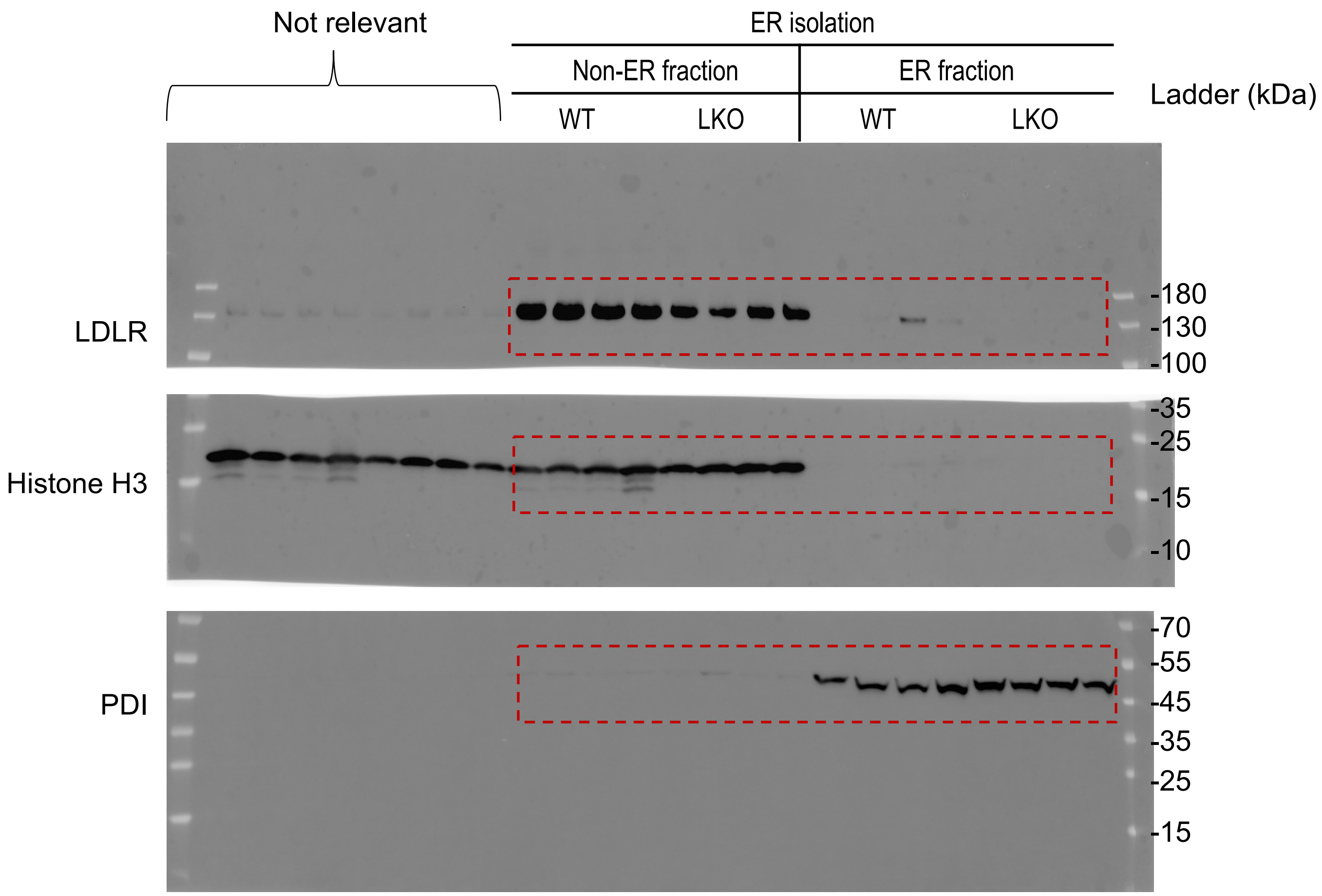

Full unedited blot for Figure 2D

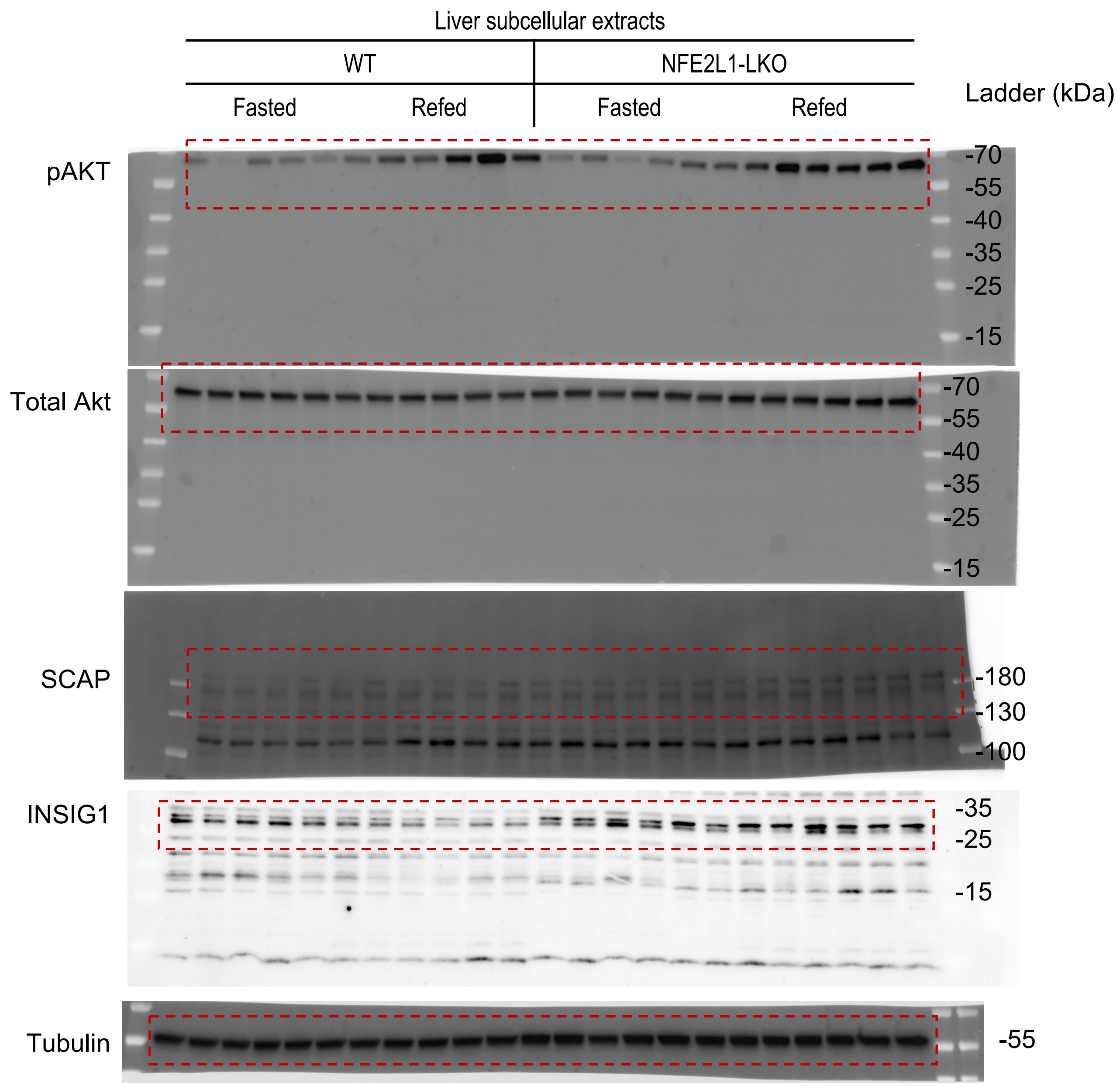

Full unedited blot for Figure 2I

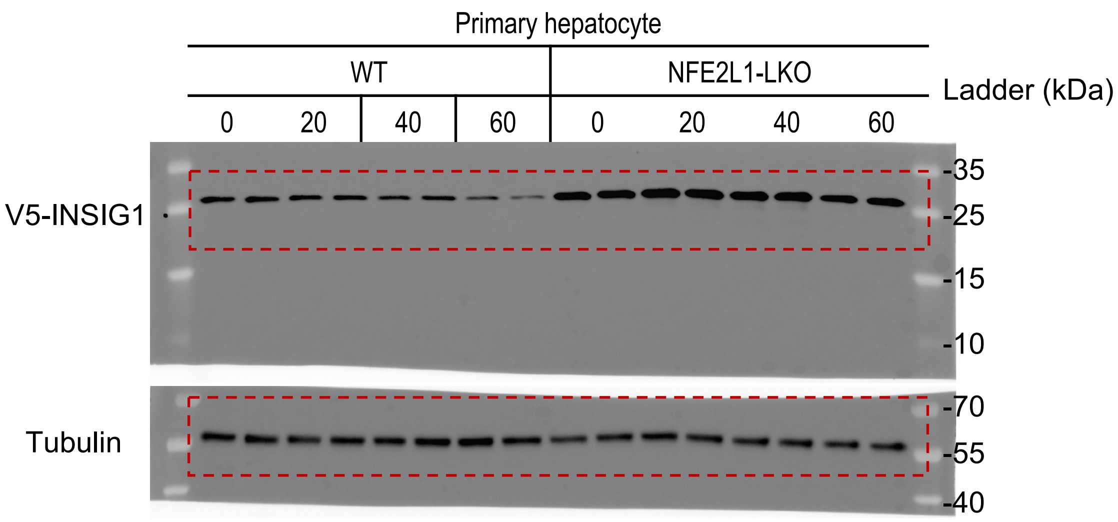

Figure 3A

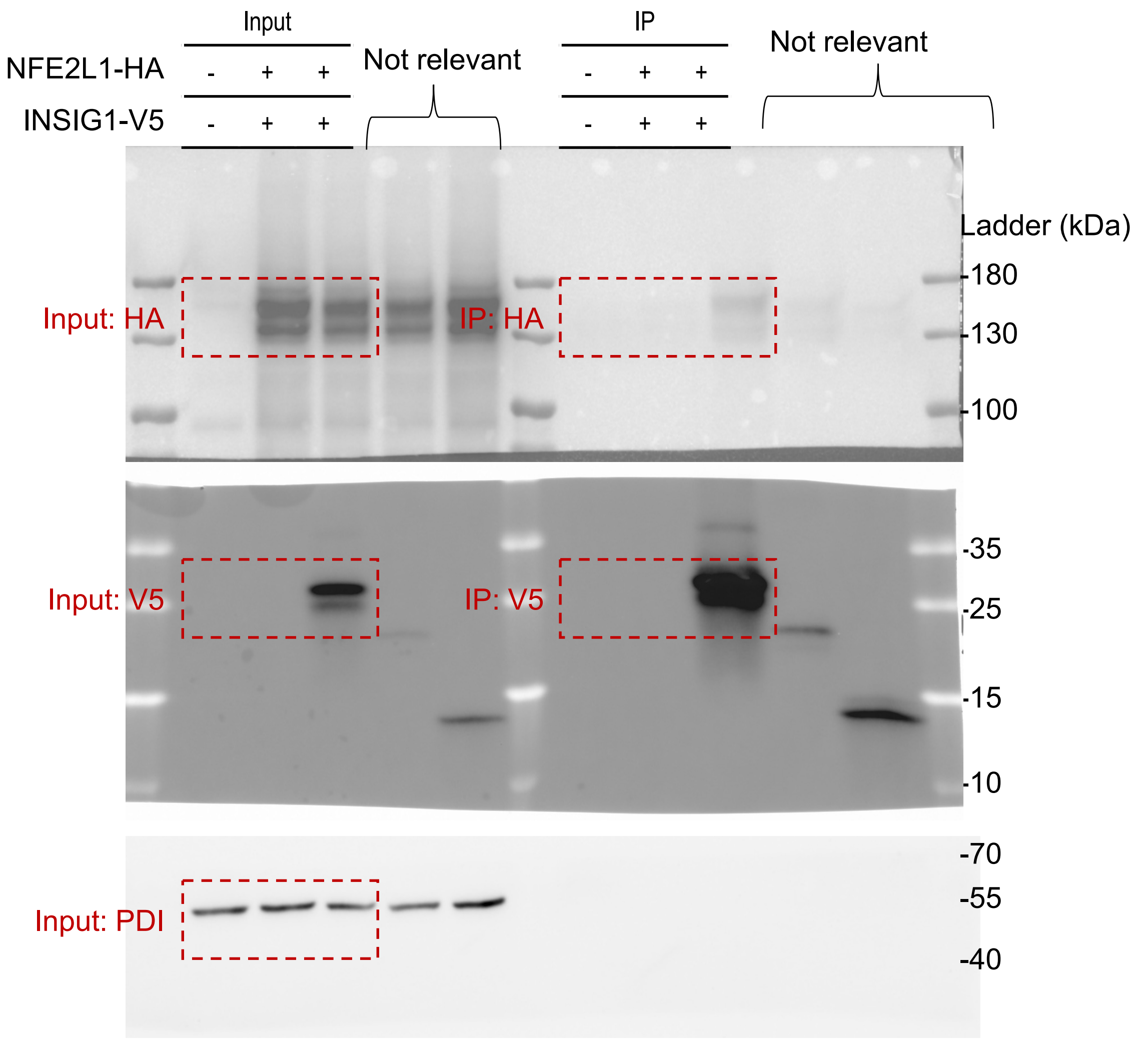

Input
IP
NFE2L1-HA - + + - + +
INSIG1-V5 - + + - + +
Not relevant
Not relevant
Ladder (kDa)
Input: HA
IP: HA
180
130
100
Input: V5
IP: V5
35
25
15
10
Input: PDI
70
55
40

Full unedited blot for Figure 3C

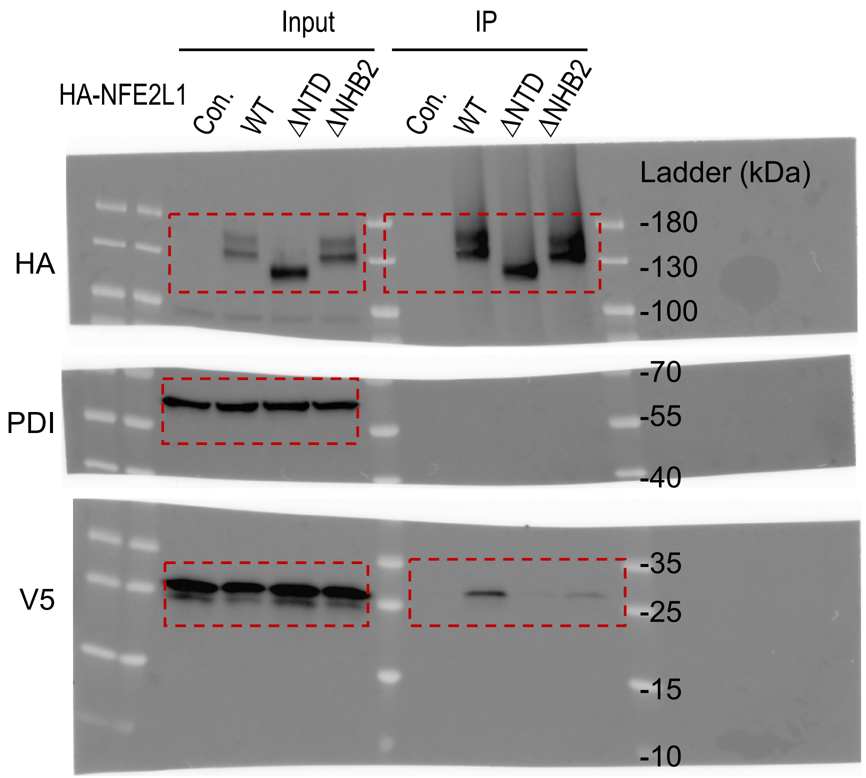

Full unedited blot for Figure 3F (IP by HA beads)

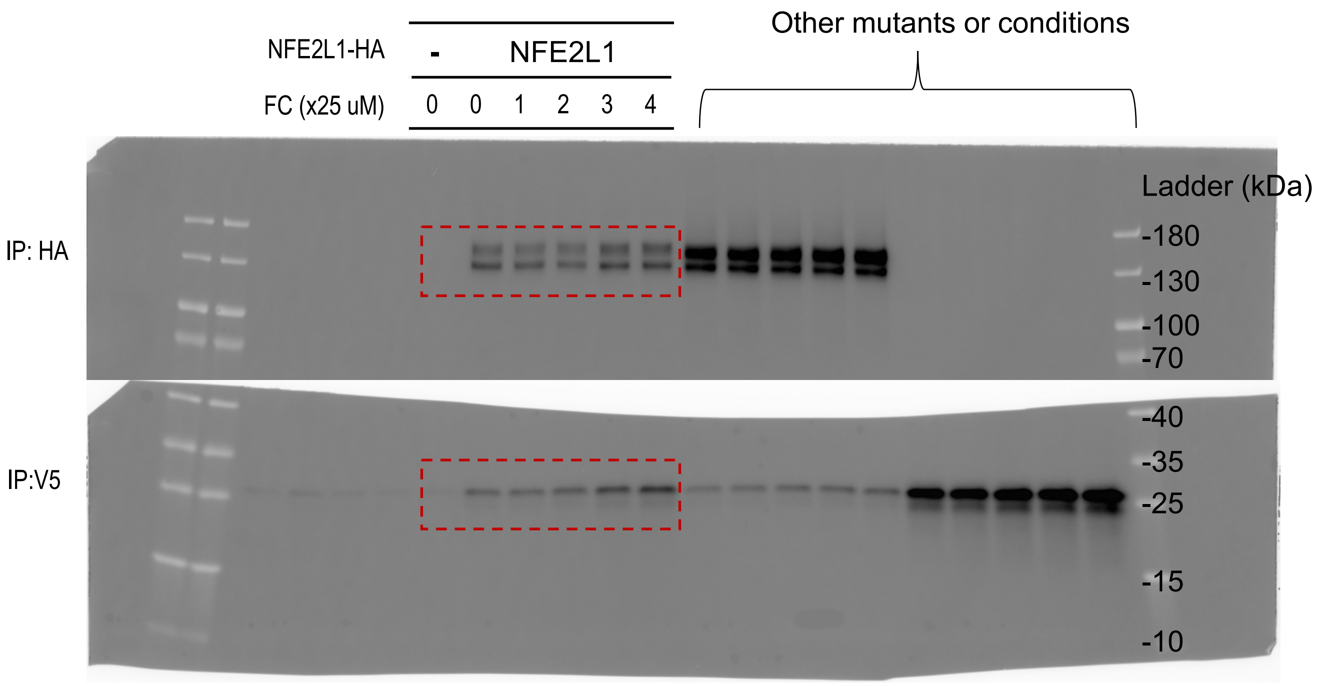

Full unedited blot for Figure 3F (whole cell input)

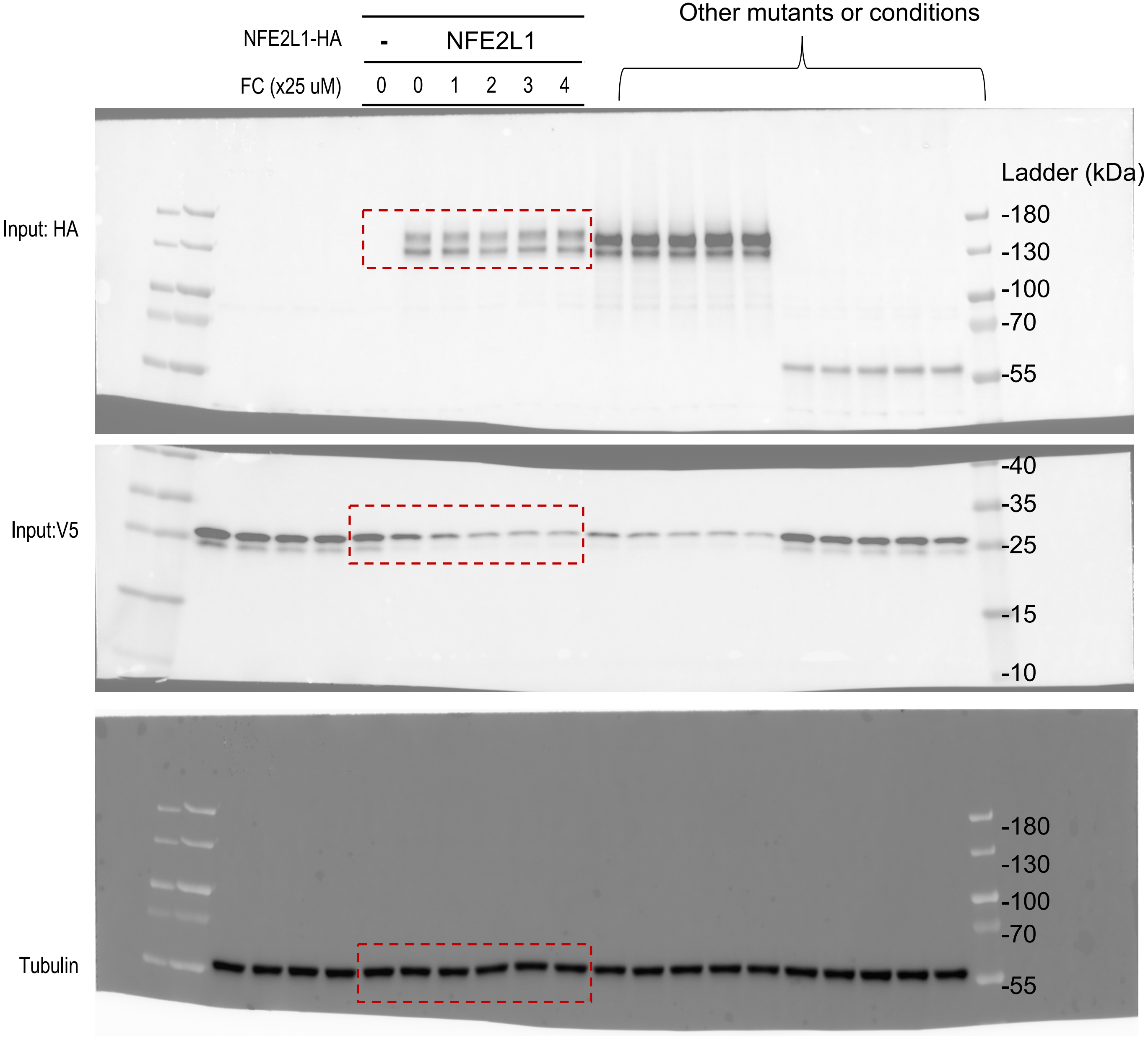

Full unedited blot for Figure 3H

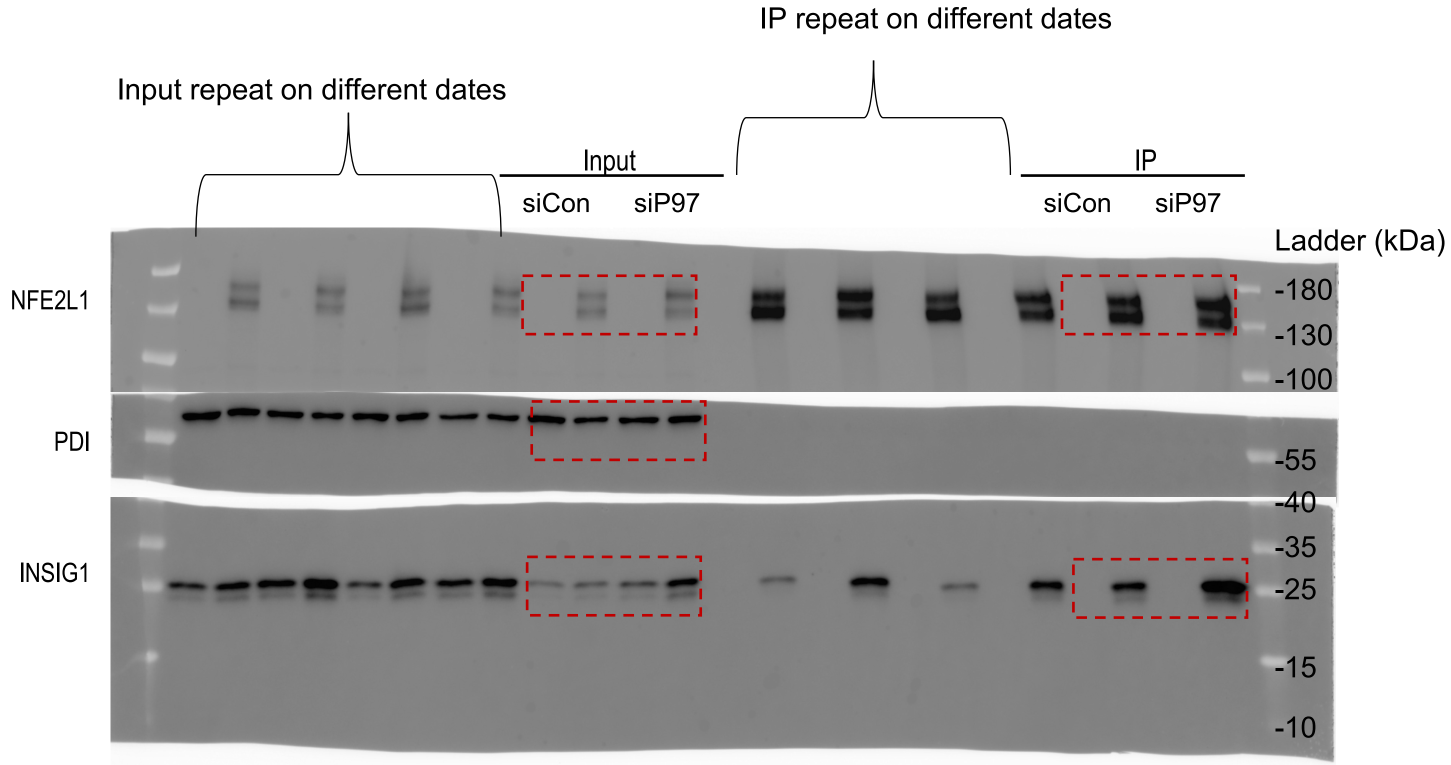

Full unedited blot for Figure 3I

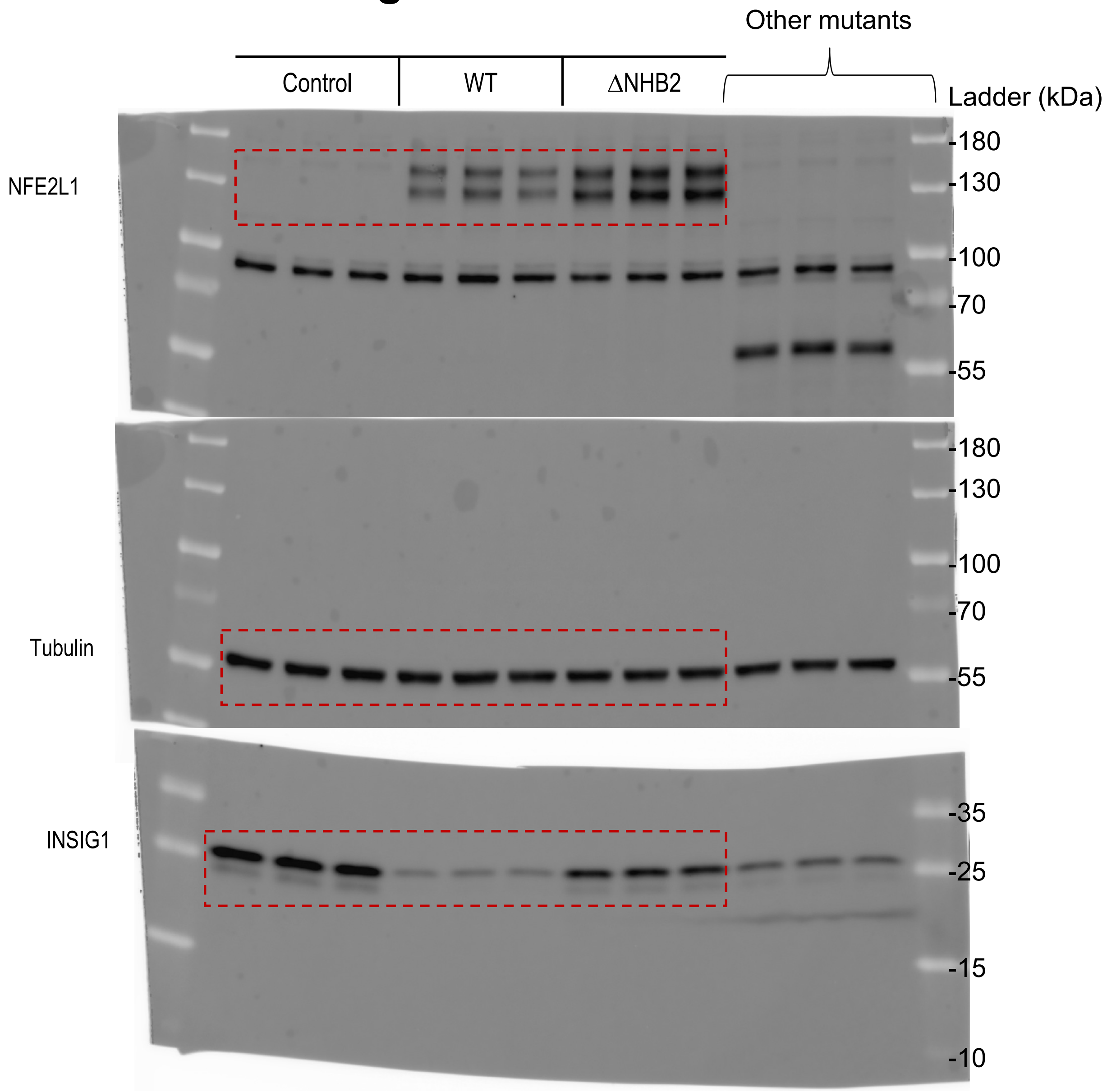

Full unedited blot for Figure 4A

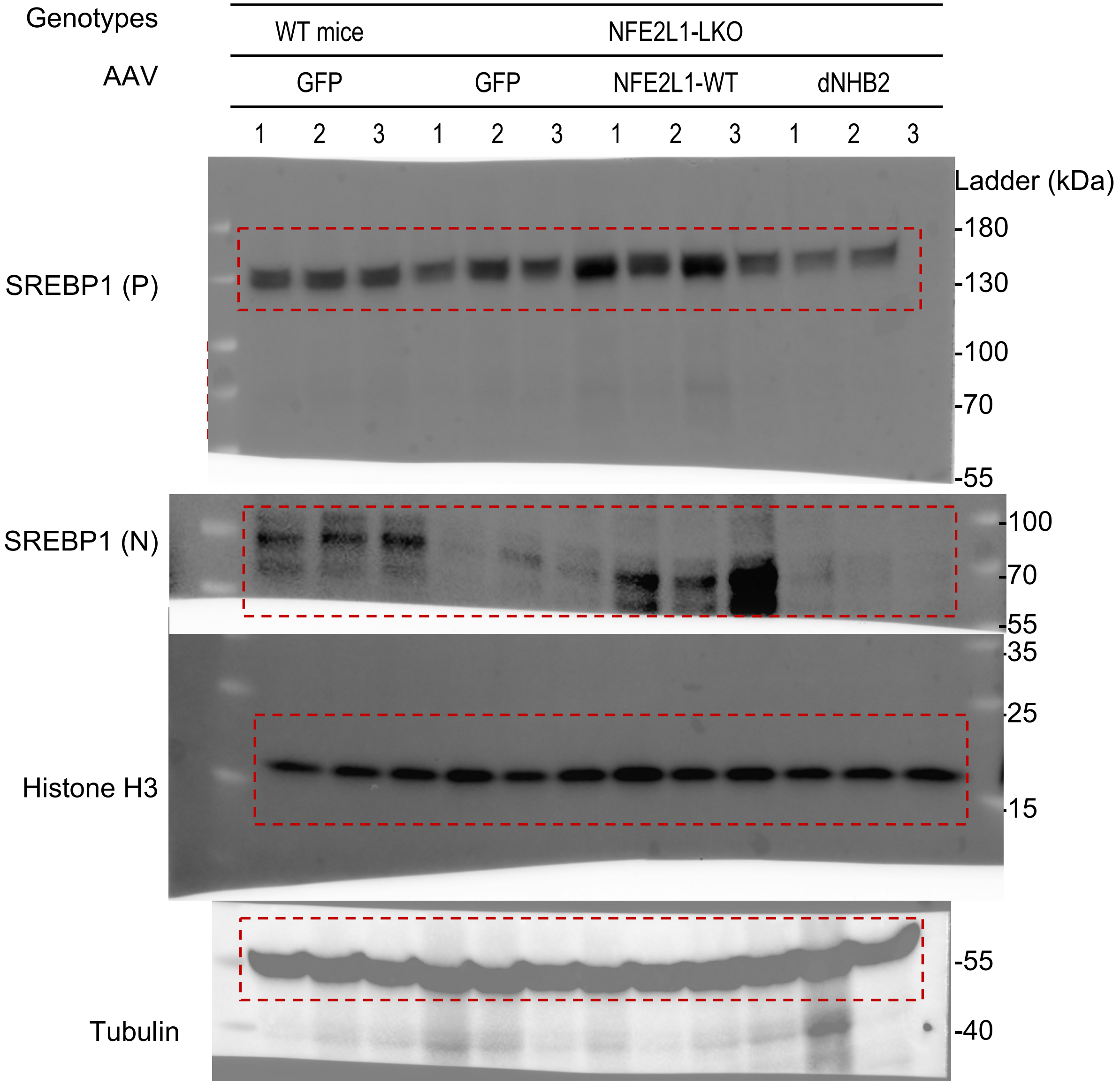

Full unedited blot for Figure 5B

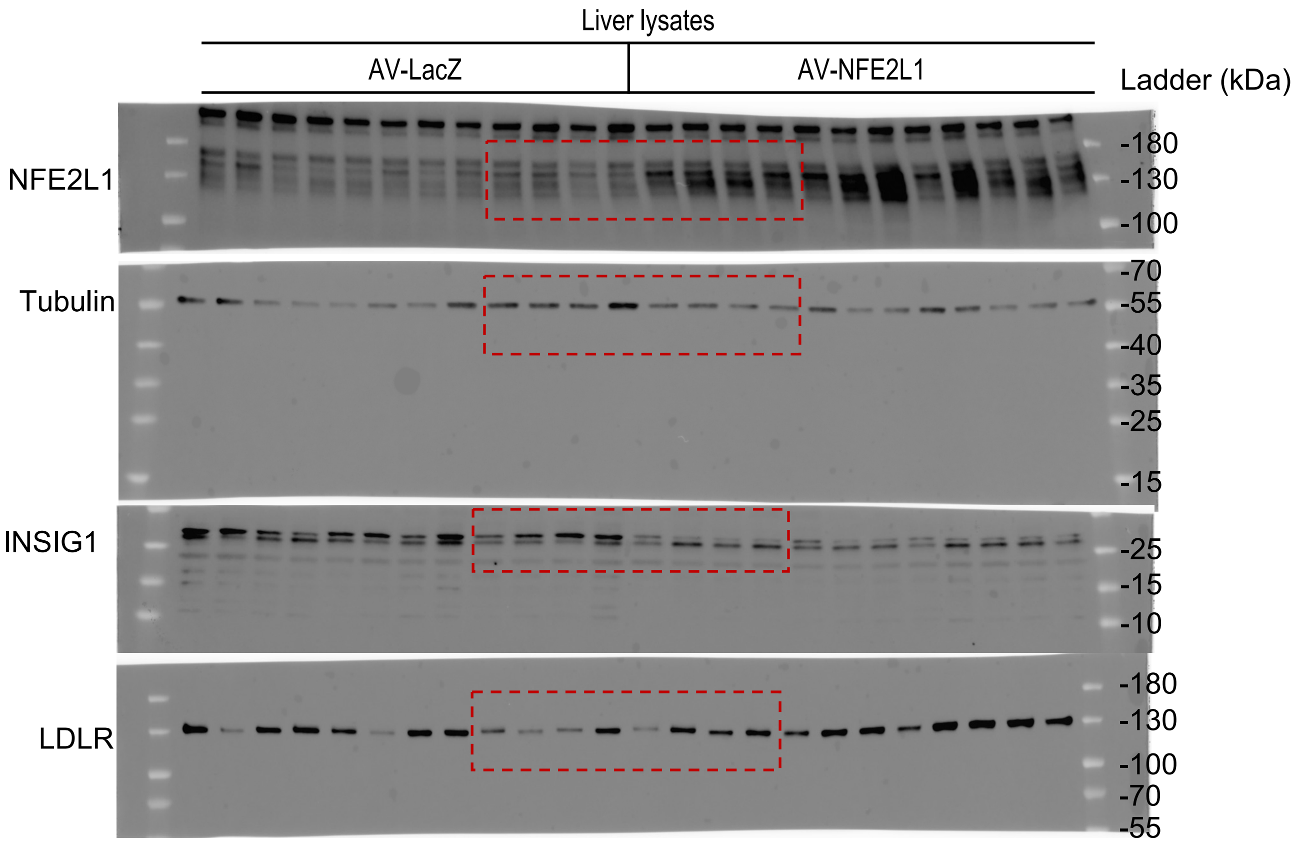

Full unedited blot for Figure S1E

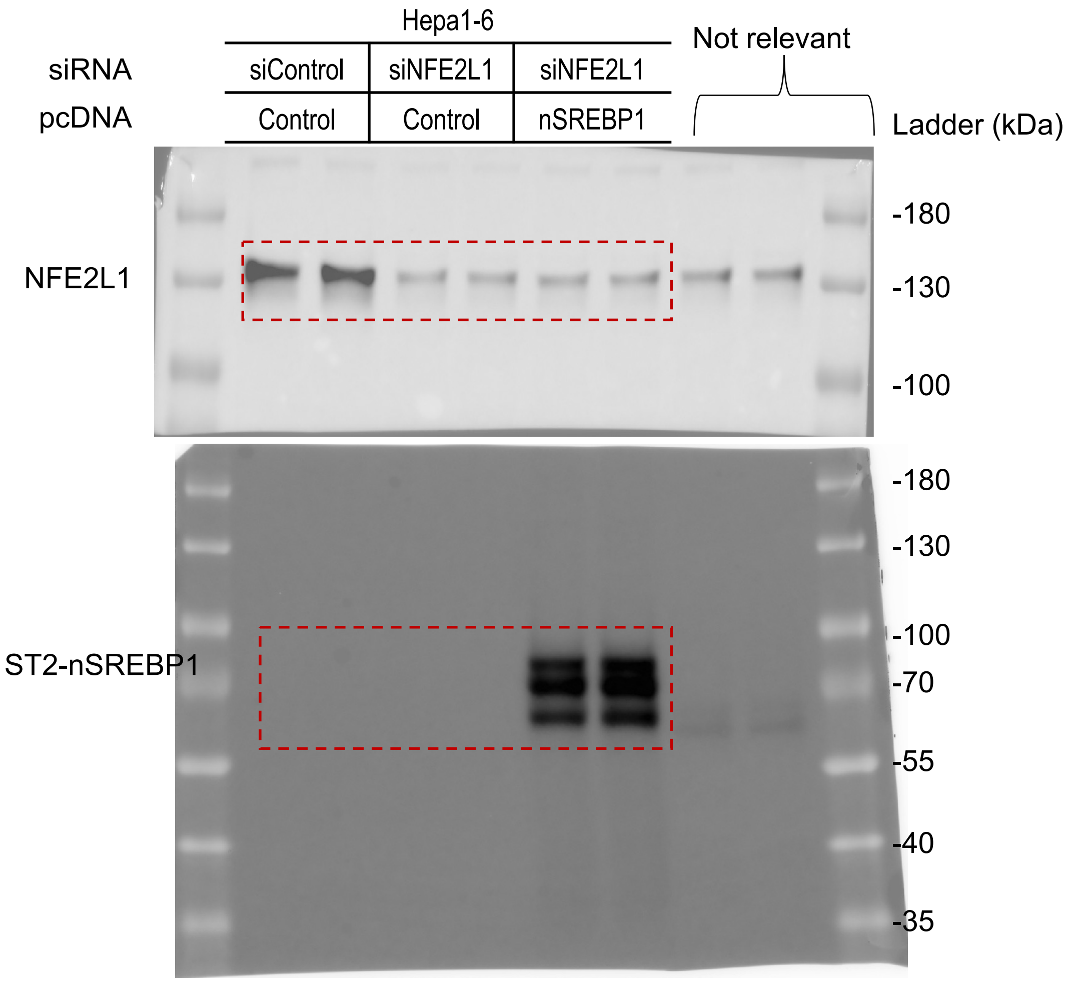

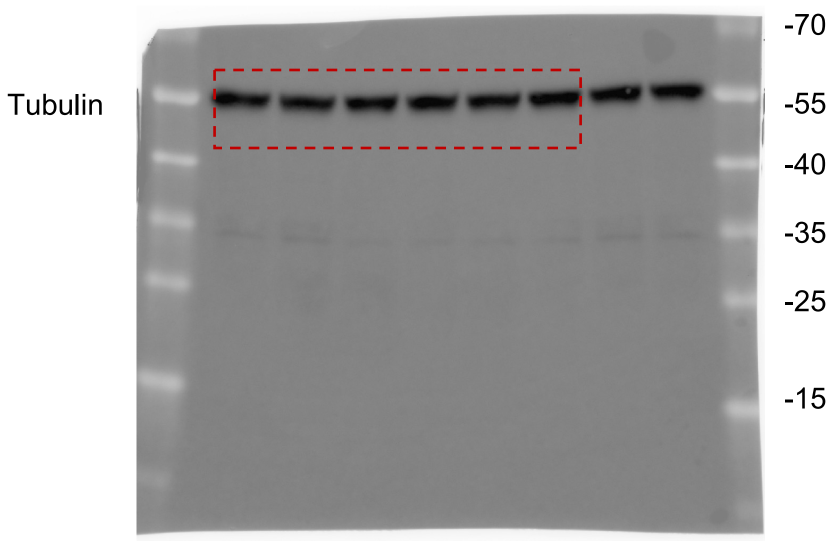

Full unedited blot for Figure S3A

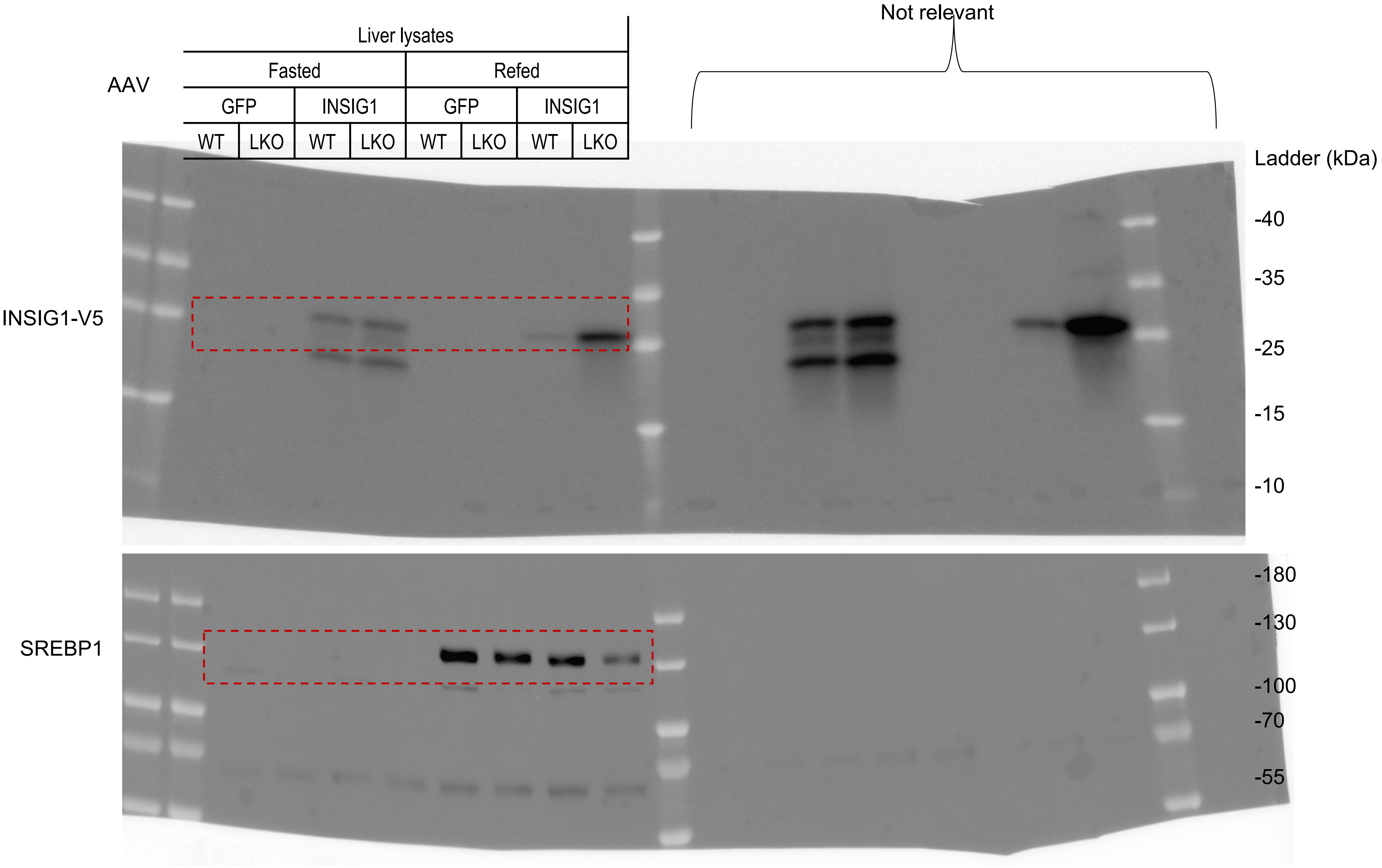

Full unedited blot for Figure S3C (1) Whole cell input

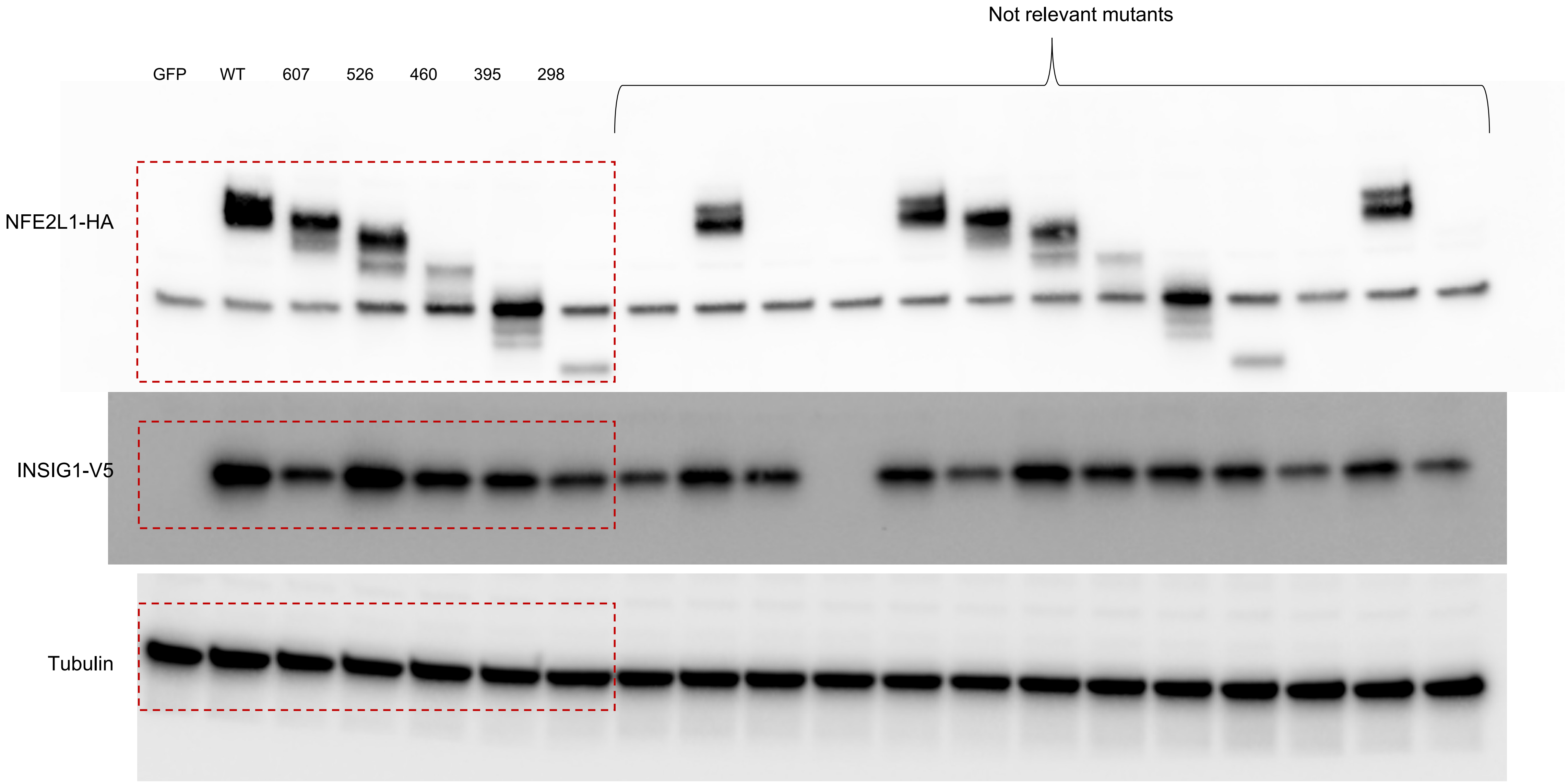

Full unedited blot for Figure S3C (2)Co-IP

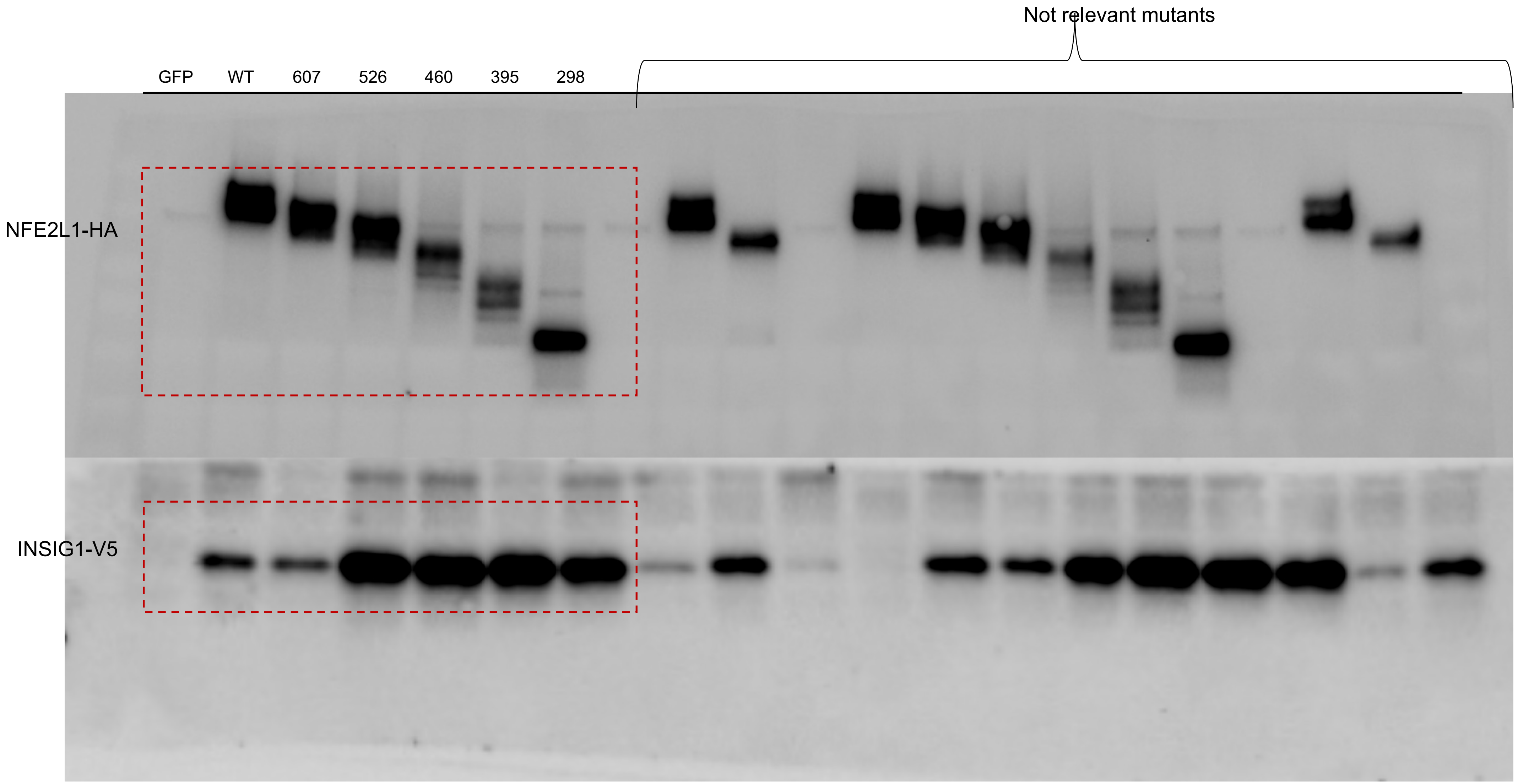

Full unedited blot for Figure S3E

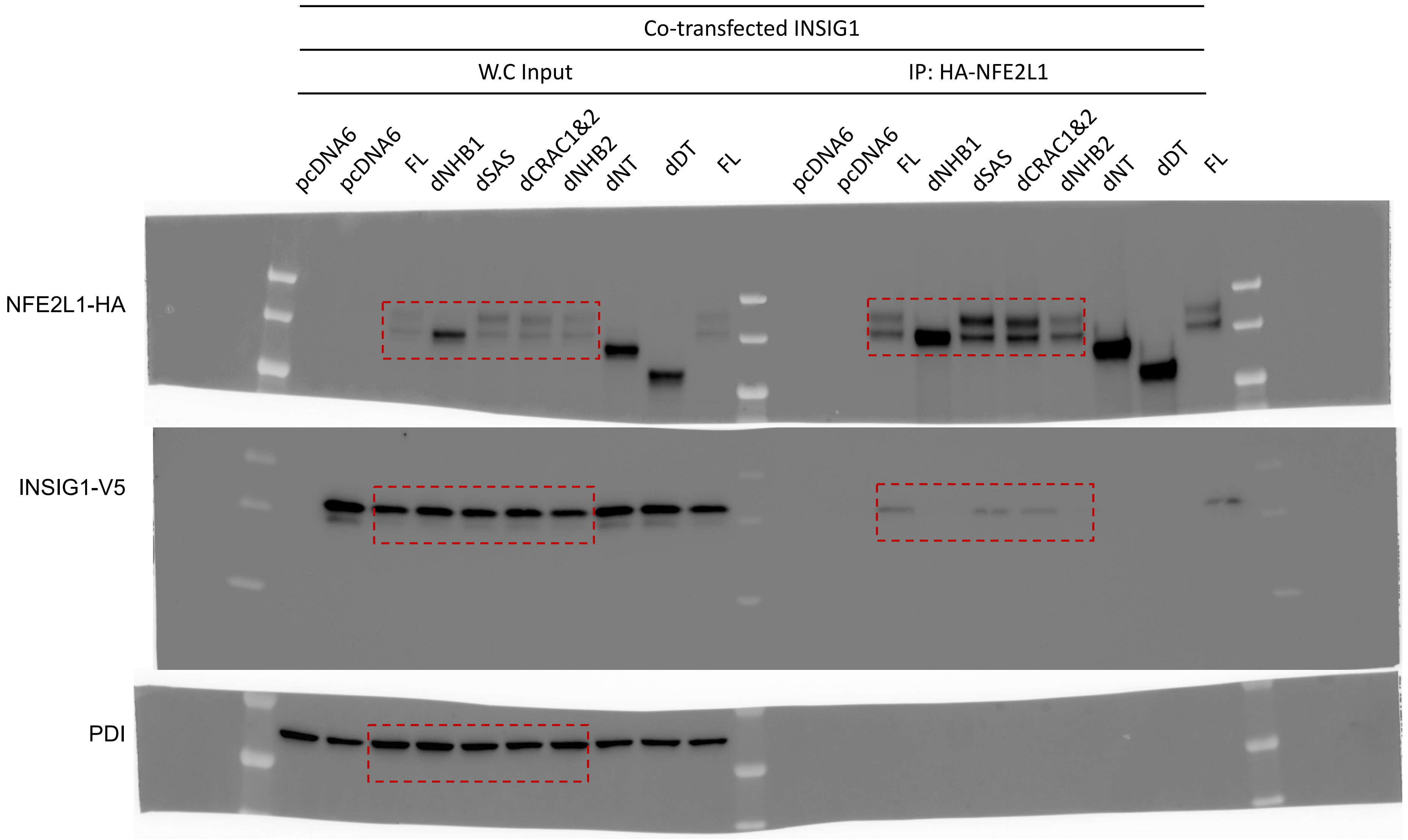

Full unedited blot for Figure S3F

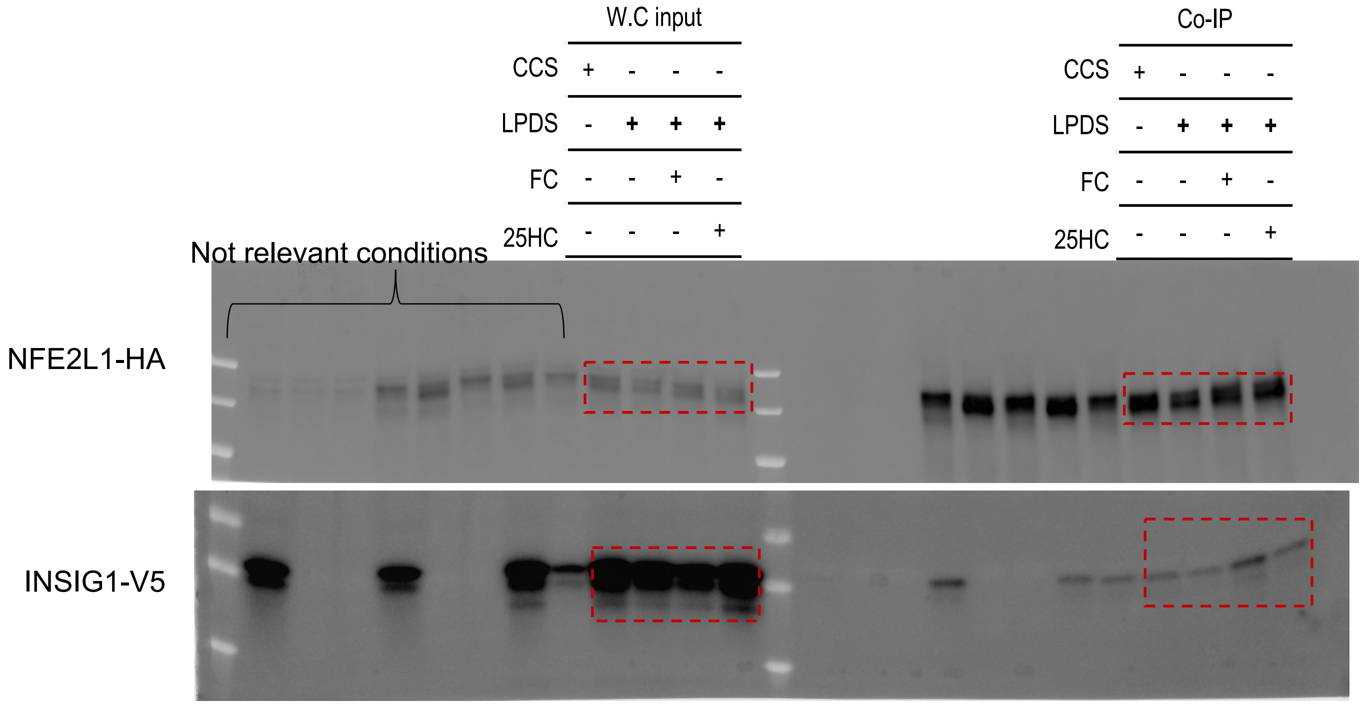

Full unedited blot for Figure S4B

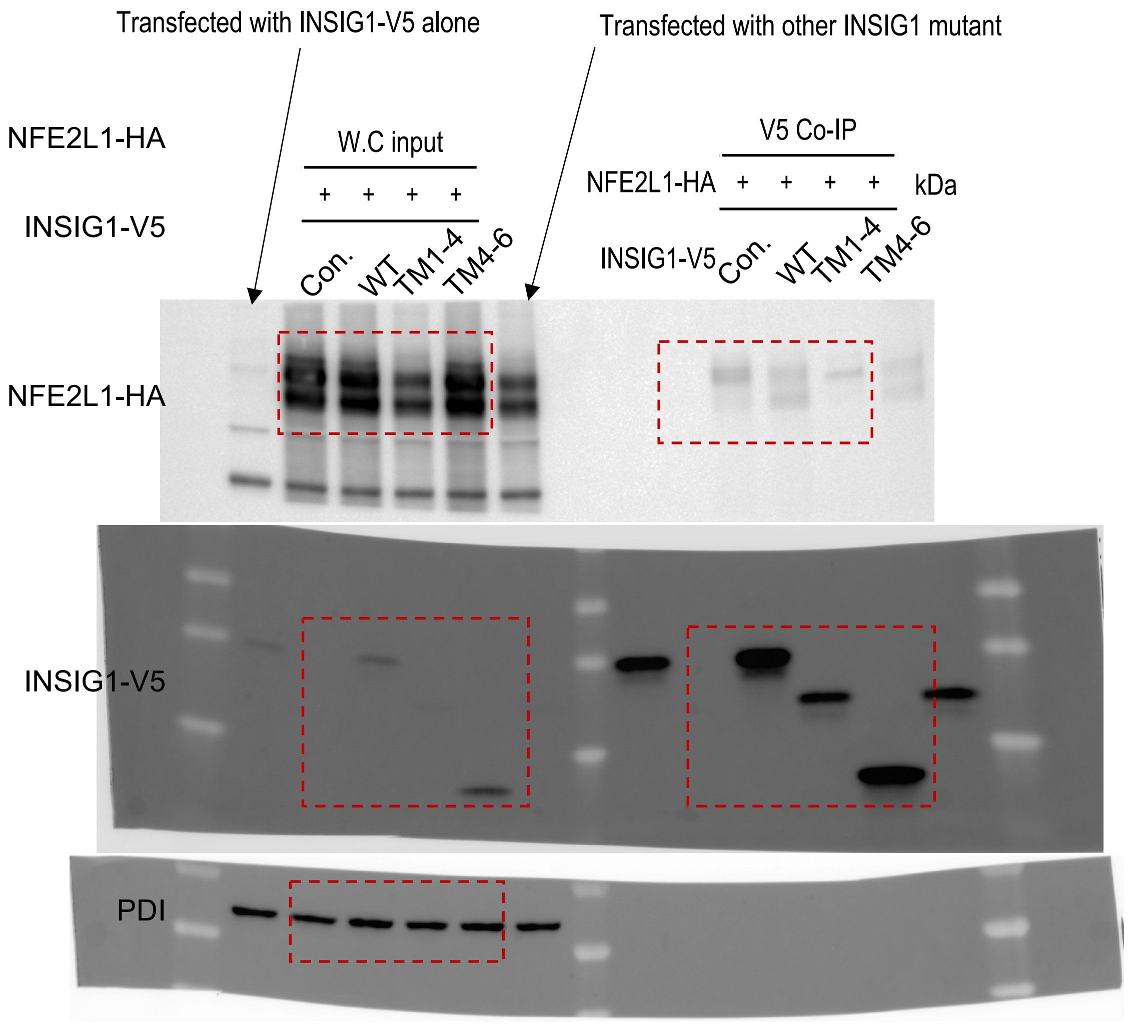

Full unedited blot for Figure S4C

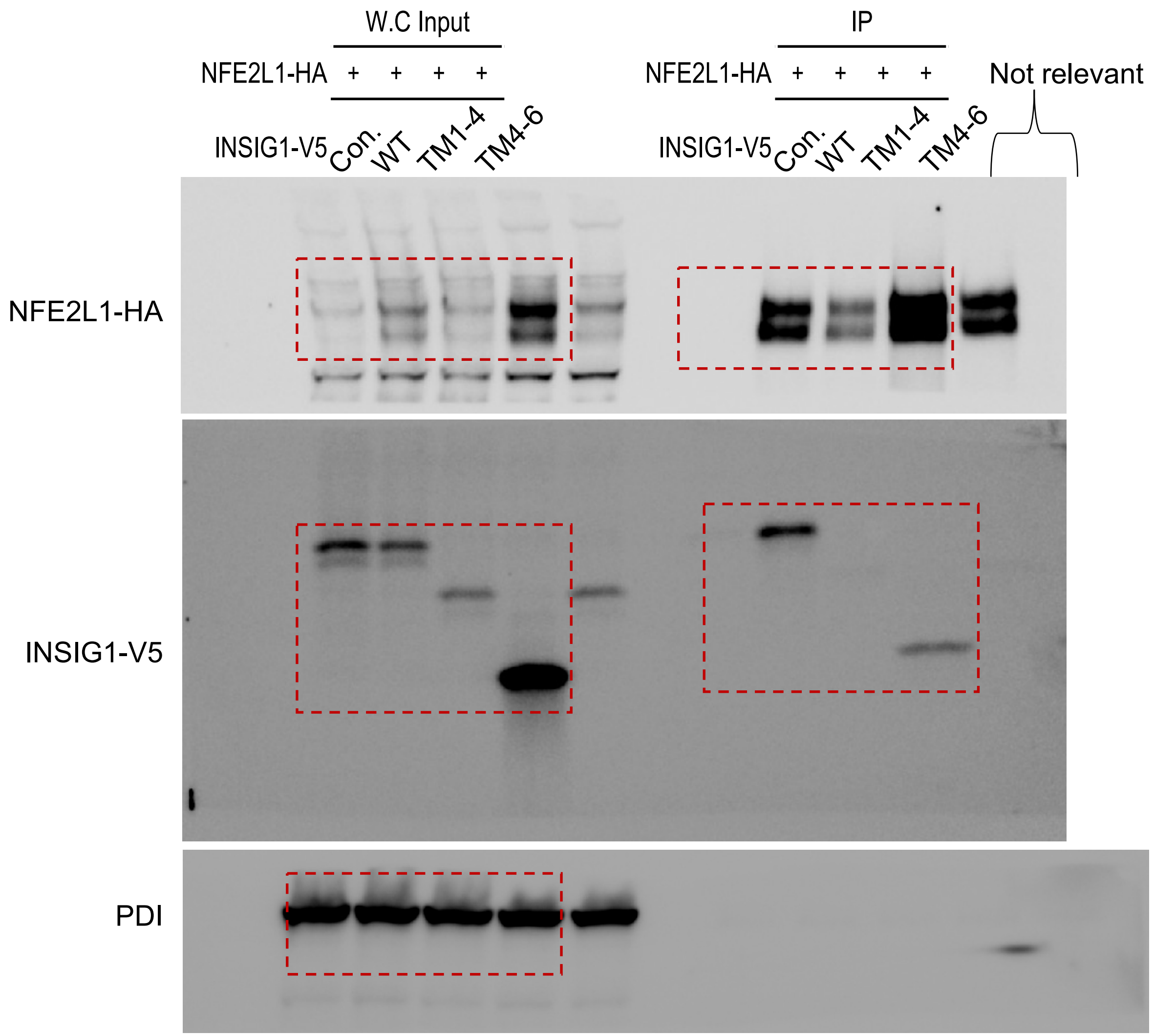

Full unedited blot for Figure S4D

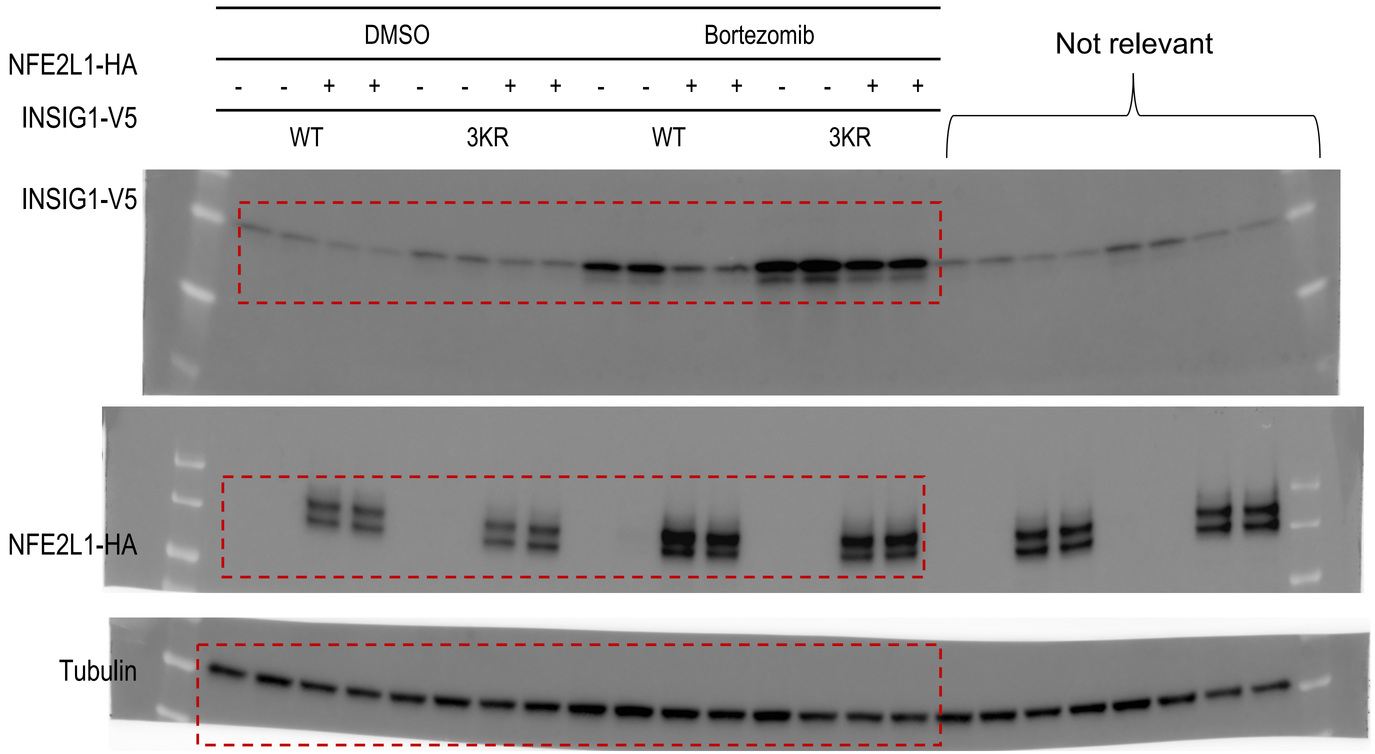

Full unedited blot for Figure S4E

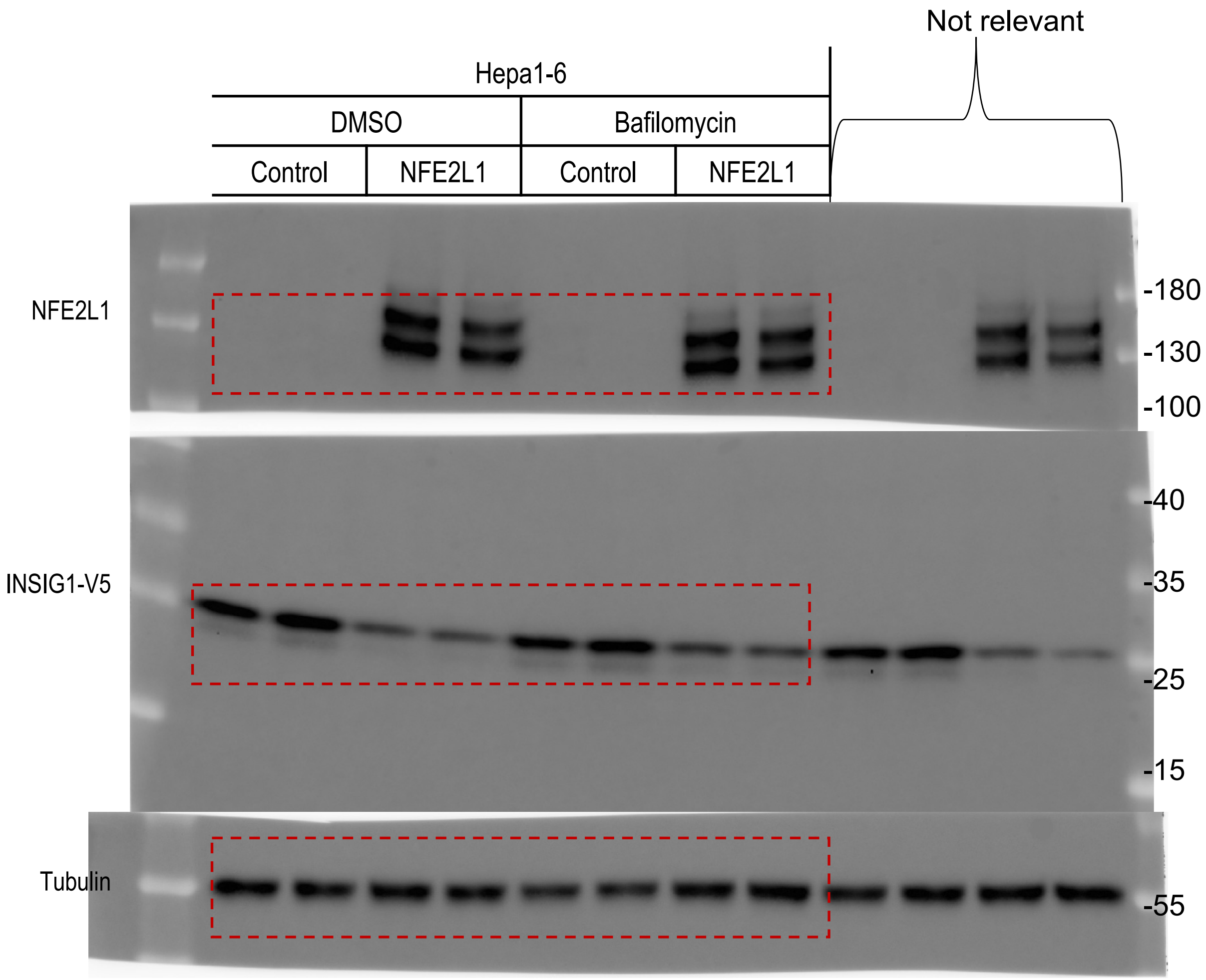

Supplement: Unedited blot and gel images [file jci-136-197094-s329.pdf]
